# Induction of CTH expression in response to amino acid starvation confers resistance to anti-LAT1 therapy in MDA-MB-231 cells

**DOI:** 10.1038/s41598-022-04987-5

**Published:** 2022-01-19

**Authors:** Takashi Yamaga, Junichi Suehiro, Youichiro Wada, Hiroyuki Sakurai

**Affiliations:** 1 Department of Pharmacology and Toxicology, Kyorin University School of Medicine, 6-20-2, Shinkawa, Mitaka, Tokyo 181-8611 Japan; 2 Isotope Science Center, The University of Tokyo, 2-11-16, Yayoi, Bunkyo-ku, Tokyo 113-0032 Japan

**Keywords:** Breast cancer, Cell growth

## Abstract

L type amino acid transporter 1 (LAT1) is an attractive molecular target for cancer therapy because of its overexpression in many cancer cells. JPH203, a selective LAT1 inhibitor, causes amino acid deprivation and suppresses cancer cell proliferation. However, several cancer cells showed resistance to amino acid deprivation. In this study, we aimed to elucidate the molecular mechanism of different sensitivity between 2 breast cancer cells to anti-LAT1 therapy. MDA-MB-231 cells were more resistant to growth suppression effect of JPH203 than T-47D cells (IC50 was 200 ± 12.5 μM for MDA-MB-231, and 5 ± 1.1 μM for T-47D cells; p < 0.05). Transcriptome and biochemical analysis were done in these cells in the presence/absence of JPH203. JPH203 induced intracellular amino acid deprivation stress in both cells, but it upregulated cystathionine γ lyase (CTH), an enzyme for synthesis of antioxidants, only in MDA-MB-231 cells. Moreover, siRNA-mediated *CTH* knockdown induced oxidative stress in response to JPH203 leading to decreased cell viability in MDA-MB-231 cells. These results suggest that activation of anti-oxidation pathways in response to amino acid deprivation confers resistance to anti-LAT1 therapy.

## Introduction

L-type amino acid transporter (LAT) family are membrane proteins that transport large neutral amino acids across the cell membrane, which consist of four members (LAT1, LAT2, LAT3 and LAT4)^1–5^. Among them, LAT1 which forms a functional heterodimer with 4F2 heavy chain (4F2hc) has been considered to be a promising target for cancer therapy^5,6^. Its expression levels are high in the fetal tissue and restricted to the barrier such as blood brain barrier or cells with rapid proliferation such as bone marrow in adults^7^. Many tumor cells express LAT1 and its levels of expression are correlated with the aggressiveness of the malignancy^8,9^. In adult non-tumor cells, large neutral amino acids are transported by LAT2^10,11^. This tumor selective expression pattern together with novel mode of action, i.e. restricting amino acids delivery specifically for cancer cells, attracted the attention of many investigators. Endou et al. developed JPH203, a specific competitive inhibitor for LAT1, which is now in clinical trial in Japan^12^.

Restricting essential amino acids uptake into the tumor cell suppresses its proliferation via inhibition of mammalian target of rapamycin (mTOR)-p70S6 Kinase (p70S6K) pathway^13–15^. If we assume that the sensitivity of mTOR to essential amino acids are similar across the cells, the potency of JPH203 for a particular cell would be predicted by the expression levels of LAT1. However, the potency of JPH203 on growth suppression was reported to be different among various cancer cell lines that express similar levels of LAT1 ^16^.

In addition to suppressing mTOR pathway, genetic inhibition or JPH203 treatment reported to activate amino acid stress response pathway, which consists of general control nonderepressible 2 (GCN2)- eukaryotic Initiation Factor 2 α (eIF2α)—activating transcription factor 4 (ATF4)^17^. When cells are depleted of amino acids, GCN2 is activated by phosphorylation and subsequently phosphorylates eIF2α, which leads to inhibition in general protein translation. Phosphorylated eIF2α promotes ATF4 translation in the face of inhibition of general protein translation^18^. Upregulation of ATF4 may facilitate apoptosis of cancer cells in some circumstances^19^, while it may also promote cancer cell survival and tumorigenesis^20^. One of the downstream molecules regulated by ATF4 is cystathionine γ lyase (CTH), a key enzyme required for synthesis of anti-oxidant molecules such as cysteine and reduced glutathione (GSH)^21^.

We hypothesized that the different degree of anti-proliferative effect of JPH203 among various cancer cells could be explained by the difference in activation of stress responsive pathway downstream of ATF4. For example, if some cells activated pro-apoptotic pathways following ATF4 induction, the proliferation or viability of these cells would be easily suppressed by JPH203. In contrast, if other cells facilitated expression of anti-oxidant molecules downstream of ATF4, these cells would be resistant to the anti-proliferative effect of JPH203.

## Results

### JPH203 differentially suppressed the proliferation of MDA-MB-231 and T-47D cells

**Figure 1:**
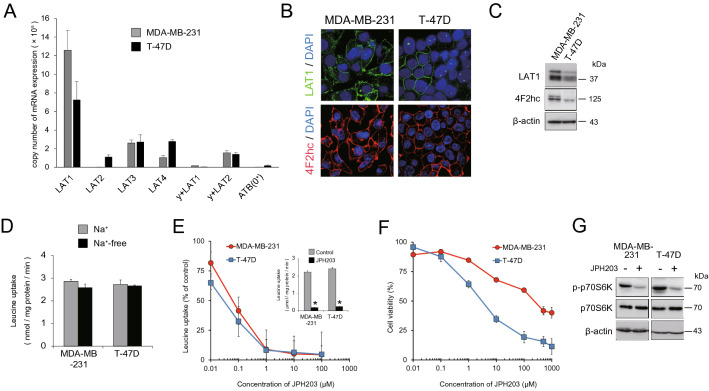
Expression of LAT family and effect of JPH203 in MDA-MB-231 and T-47D cells. (**A**) Copy number of leucine transporter mRNA in MDA-MB-231 and T-47D cells by qPCR. (**B**) Immunocytochemistry of LAT1 and 4F2hc in MDA-MB-231 and T-47D cells. Upper columns: LAT1 (green) and DAPI (blue; nuclear staining), lower columns: 4F2hc (red) and DAPI. Scale bars = 20 μm. (**C**) Western blot analysis of LAT1 and 4F2hc in MDA-MB-231 and T-47D cells. (**D**) Radio-labeled leucine uptake was measured in normal Na^+^-containing or Na^+^-free buffer. (**E**) Radio labeled l-leucine uptake was measured under condition of 0.01–100 μM JPH203. The inset showed leucine uptake at a concentration of 1 μM JPH203 in MDA-MB-231 and T-47D cells. *p < 0.05. (**F**) MDA-MB-231 and T-47D cells were incubated for 4 days in the presence of indicated concentration of JPH203, and cell viability was assessed by MTT colorimetric assay. (**G**) MDA-MB-231 and T-47D cells were incubated for 12 h in the absence or presence of 100 μM (MDA-MB-231) and 5 μM (T-47D) of JPH203. The cell lysates were subjected to western blot analysis for phospho-p70S6K, a target molecule downstream of mTOR.

Two different breast cancer cell lines were used for the experiments; MDA-MB-231 cells derived from triple negative (i.e., no expression of estrogen receptor, progesterone receptor, or HER2) breast cancer, generally classified as more aggressive, treatment resistant cancer, and T-47D cells derived from ductal carcinoma of the breast with estrogen receptor, progesterone receptor, and HER2 expression. We confirmed that *LAT1* was the most abundantly expressed leucine transporter both in MDA-MB-231 and T-47D cells by qPCR (Fig. 1A). Its plasma membrane expression was apparent as shown in Fig. 1B. Both in mRNA and protein level, the amount of LAT1 in MDA-MB-231 cells were about 1.5-fold more than that in T-47D cells (Fig. 1A,C).

Both of these cells took up comparable amount of leucine in Na^+^-free buffer compared with that in Na^+^ containing buffer (Fig. 1D) and contribution of Na^+^-dependent leucine uptake was minimal, consistent with low expression of Na^+^-dependent leucine transporters such as y^+^LAT1(SLC7A7), y^+^LAT2(SLC7A6), or ATB(0^+^) (SLC6A14) (Fig. 1A). JPH203 has been shown to act as a highly specific competitive inhibitor of LAT1^12^. IC50 of JPH203 for leucine uptake inhibition (IC50_uptake_) were 0.06 ± 0.02 μM for MDA-MB-231 cells and 0.03 ± 0.01 μM for T-47D cells (p = 0.070; Fig. 1E) and leucine uptake of both of these cells was almost completely inhibited by 1 μM of JPH203 (Fig. 1E, inset). These functional data together with expression data suggest that uptake of large neutral amino acids including leucine into these cells was almost entirely dependent on LAT1_._

However, proliferation of T-47D cells was suppressed in the presence of lower concentrations of JPH203 than that for MDA-MB-231 cells; IC50_proliferation_ of JPH203 for T-47D cells was 5 ± 1.1 μM while that for MDA-MB-231 cells was 200 ± 12.5 μM (p < 0.05; Fig. 1F). Hereafter, we describe relative lack of efficacy of JPH203 in suppressing cell proliferation in MDA-MB-231 cells as JPH203 “resistance”.

Consistent with previous reports, inhibition of leucine and other branched chain amino acids uptake by JPH203 decreased the activation of mTORC1 pathway and the degree of inhibition was similar between these cells (Fig. 1G). In addition, a molecule in the amino acid nutritional stress pathway CHAC1^22^, which degrades antioxidant GSH, was upregulated in response to JPH203 in both cells (Supplementary Fig. 1), suggesting that some of the reactions to amino acids starvation were commonly observed in both of these cells.

### JPH203 treatment induced CTH in MDA-MB-231 cells but not in T-47D cells

**Figure 2:**
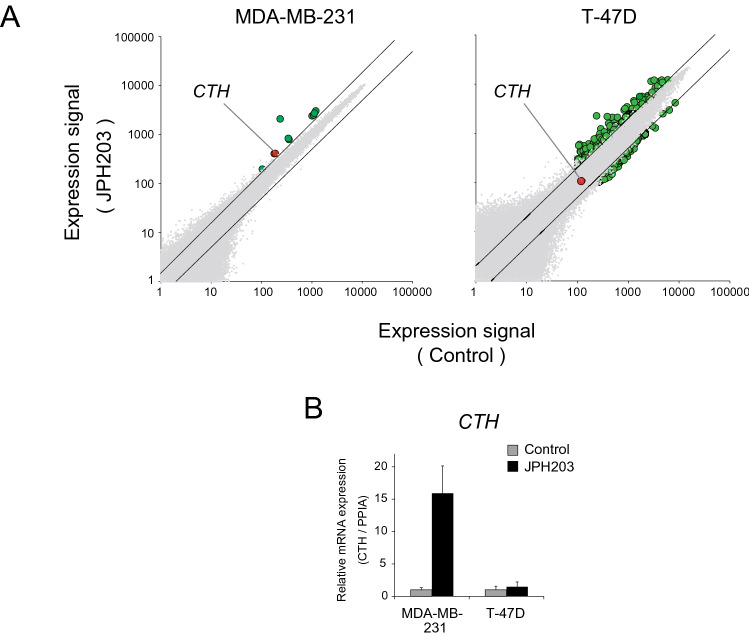
Transcriptome analysis of MDA-MB-231 and T-47D cells in the presence/absence of JPH203. (**A**) DNA microarray was performed in the presence of 100 μM JPH203 for 24 h in MDA-MB-231 and T-47D cells. A gene was regarded as differentially expressed if the signal intensity of JPH203 treated condition/signal intensity in control condition was ≤ twofold or ≥ twofold (green dots, *CTH* was designated by red dots). (**B**) *CTH* mRNA expression in the presence/absence of 100 μM JPH203 for 12 h in MDA-MB-231 and T-47D cells by qPCR.

Consistent with the relative resistance to JPH203 treatment, the transcriptome of MDA-MB-231 cells was not much affected by JPH203 (Fig. 2A); only 6 genes were differentially expressed in the presence of JPH203 (Table 1). On the other hand, 516 genes were differentially expressed in T-47D cells (Fig. 2A).

**Table 1:** Differentially expressed genes in MDA-MB-231 cells by JPH203 treatment.

Gene	Function	Ratio : JPH203/control	
		MDA-MB-231	T-47D
*ASNS*	Asparagine biosynthetic process, cellular response to glucose starvation	8.57	4.29
*PSAT1*	l-Serine biosynthetic process	2.64	1.74
*CYP1B1*	Metabolism of various endogenous substrates	2.46	1.09
*CTH*	Cysteine biosynthetic process, sulfur amino acid catabolic process	2.46	0.66
*NNMT*	NAD biosynthesis via nicotinamide riboside salvage pathway	2.38	0.50
*KIAA1199*	Hyaluronan biosynthetic process	2.30	5.28

Of these 6 genes upregulated by JPH203 treatment in MDA-MB-231 cells, 2 genes, *cystathionine γ lyase* (*CTH*) and *nicotinamide N-methyltransferase* (*NNMT*) were upregulated only in MDA-MB-231 cells (Table 1). Interestingly, both of these enzymes act in an anti-oxidative pathway that generates cysteine from methionine^23–25^. After confirming that *CTH* mRNA was upregulated in response to JPH203 only in MDA-MB-231 cells by qPCR (Fig. 2B), we decided to focus on CTH, a more downstream enzyme in the process.

### CTH was likely to be induced through GCN2-ATF4 pathway in MDA-MB-231 cells

**Figure 3:**
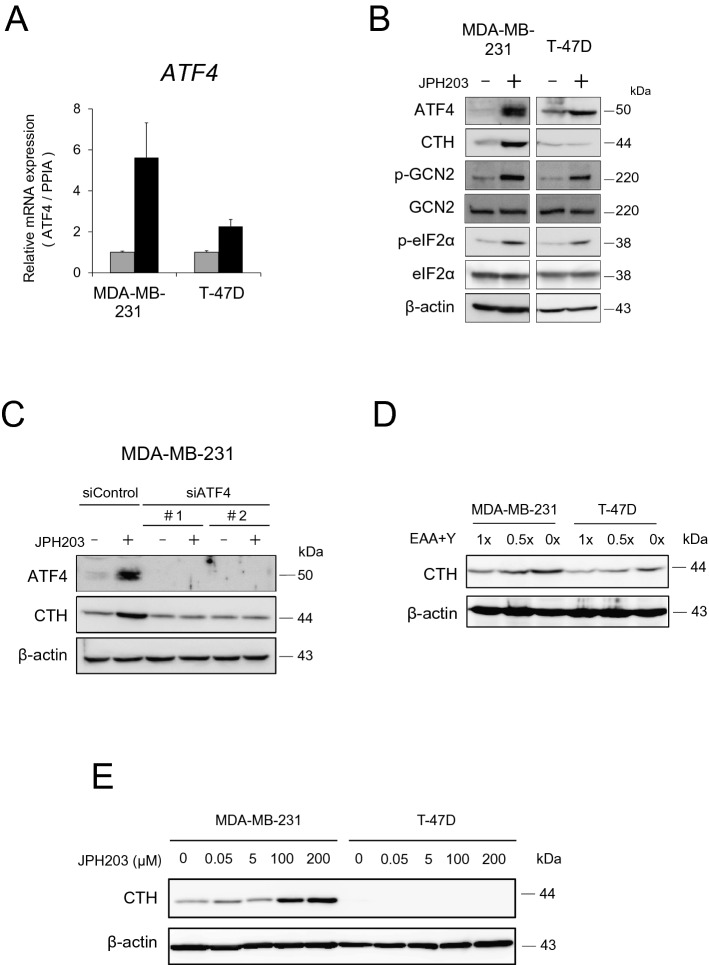
Activation of amino acid starvation stress pathway in cells in response to JPH203 or LAT1 substrate amino acids restriction. (**A**) *ATF4* mRNA expression in the presence/absence of 100 μM JPH203 for 12 h in MDA-MB-231 and T-47D cells by qPCR. (**B**) MDA-MB-231 and T-47D cells were incubated for 12 h in the absence or presence of 100 μM (MDA-MB-231) and 5 μM (T-47D) of JPH203. The cell lysates were subjected to western blot analysis for ATF4, CTH, phospho-GCN2, phospho-eIF2α. (**C**) MDA-MB-231 cells were transfected with control (scrambled) or *ATF4* siRNA. These transfected cells were incubated in the presence/absence of 100 μM of JPH203 for 12 h. The cell lysates were subjected to western blot and probed for CTH and β-actin (loading control). (**D**) After MDA-MB-231 and T-47D cells were incubated for 72 h in amino acid restriction media, the cell lysates were subjected to western blot analysis and probed for CTH and β-actin (loading control). LAT1 substrate amino acids, which are eight essential amino acids (EAA; l-isoleucine, l-phenylalanine, l-tryptophan, l-valine, l-histidine, l-leucine and l-methionine) and l-tyrosine, were removed from normal RPMI (1.0) to 50% (0.5), or free (0). (**E**) MDA-MB-231 and T-47D cells were treated with JPH203 at different concentrations (0, 0.05, 5, 100 or 200 µM) for 48 h. The cell lysates were subjected to western blot analysis for determining CTH protein levels.

In order to elucidate the pathway leading to CTH upregulation, differentially expressed genes in T-47D cells in response to JPH203 treatment were analyzed. Amino acid restriction was known to induce cellular stress response known as GCN2-eIF2α-ATF4 pathway and genes related to this pathway were overrepresented among upregulated genes in T-47D cells in response to JPH203 (Table 2)^17,26,27^. Although *ATF4* was not differentially expressed in MDA-MB-231 cells in microarray experiments, it did show upregulation in response to JPH203 treatment in these cells by qPCR (Fig. 3A). Consistent with the result of transcriptome analysis, *ATF4* gene was also upregulated in T-47D cells in response to JPH203 by qPCR (Fig. 3A).

**Table 2:** GO enrichment analysis of differentially expressed genes by JPH203 treatment in T-47D cells.

	GO term (biological process)	p-value	Gene
GO:0031325	Positive regulation of cellular metabolic process	0.004	*FASN, ACACA*
GO:0006520	Cellular amino acid metabolic process	0.009	*ATF4, SDSL*
GO:0000082	G1/S transition of mitotic cell cycle	0.019	*MNAT1, CDKN3*
GO:0006281	DNA repair	0.024	*PARPBP, FANCL*
GO:0036499	PERK-mediated unfolded protein response	0.024	*ATF4, DDIT3*
GO:0006974	Cellular response to DNA damage stimulus	0.028	*DDIT3, YAP1*
GO:2001237	Negative regulation of extrinsic apoptotic signaling pathway	0.043	*CTTN, YAP1*
GO:0034976	Response to endoplasmic reticulum stress	0.068	*ATF4, DDIT3*

Because activities of GCN2 and eIF2α were regulated by phosphorylation rather than transcription, their protein level expression and phosphorylation levels were examined. Both in MDA-MB-231 cells and T-47D cells, JPH203 treatment increased phosphorylation of GCN2 and eIF2α without apparent changes in total protein expression levels (Fig. 3B). As expected from qPCR data, ATF4 protein expression was increased in both of these cells by JPH203, but CTH protein expression was increased only in MDA-MB-231 cells in response to JPH203 (Fig. 3B). This increased expression of CTH was abolished by knocking down *ATF4* (Fig. 3C), suggesting that ATF4 is at least one of the regulators for CTH expression.

To confirm that CTH is induced by amino acid depletion but not by unknown action of JPH203 per se, both cells were incubated in the medium depleted of LAT1 substrate amino acids, which are essential amino acids and tyrosine. As shown in Fig. 3D, CTH expression was higher at baseline and increased by the treatment much stronger in MDA-MB-231 cells than in T-47D cells. Along the same line, more than 100 μM of JPH203 did not increase CTH expression in T-47D cells while those doses of JPH203 clearly upregulate CTH expression in MDA-MB-231 cells (Fig. 3E). If there were unknown side effect of JPH203 that stimulates CTH expression, T-47D cells would have increased CTH in response to higher doses of JPH203. Although this is not a direct proof, it is very difficult to argue that the observed upregulation of CTH was due to some unknown side effect (i.e. not mediated by amino acid depletion) of JPH203.

### GCN2, ATF4 or CTH knockdown enhances growth inhibitory effect of JPH203 in MDA-MB-231 cells

**Figure 4:**
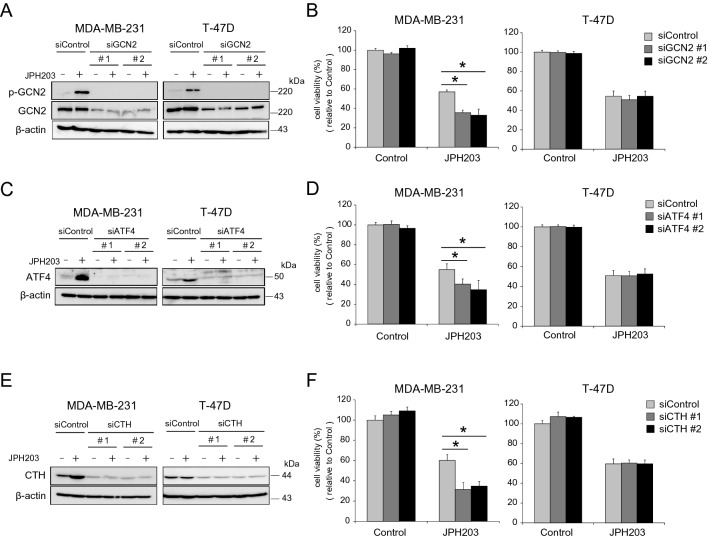
Effect of JPH203 in *GCN2*, *ATF4*, or *CTH* knockdown cells. (**A**,**C**,**E**) MDA-MB-231 and T-47D cells were transfected with control (scrambled) or *GCN2* (**A**), *ATF4* (**C**), *CTH* (**E**) siRNA. These transfected cells were incubated in the presence/absence of 100 μM (MDA-MB-231) or 5 μM (T-47D) of JPH203 for 12 h. The cell lysates were subjected to western blot and probed for each targeted molecule and βactin (loading control). (**B**,**D**,**F**) These knockdown cells were cultured in the absence or presence of 100 μM (MDA-MB-231) or 5 μM (T-47D) of JPH203 for 48 h and cell viability were assessed by MTT assay. *p < 0.05.

Previous reports showed that CTH contributed to cancer cell survival against anti-tumor therapy via enhancing antioxidant activities of the cell^25^. To examine whether amino acid starvation stress-mediated CTH induction plays a role in resisting to growth inhibition by JPH203, we performed MTT cell proliferation/viability assay using *GCN2*, *ATF4* or *CTH* knockdown cells. Two independent siRNA oligos targeted to *GCN2*, *ATF4* and *CTH* gene (si*GCN2* #1, si*GCN2* #2, si*ATF4* #1, si*ATF4* #2, si*CTH* #1 and si*CTH* #2) significantly suppressed protein expression of each gene in both MDA-MB-231 and T-47D cells (Fig. 4A,C,E). si*GCN2* #1 or si*GCN2* #2 oligos significantly decreased cell viability by 17.6% and by 26.1% in JPH203-treated MDA-MB-231, respectively (Fig. 4B). Also, si*ATF4* #1 or si*ATF4* #2 oligos inhibited cell growth by 15.2% and by 17% in MDA-MB-231, respectively (Fig. 4D). *CTH* knockdown (si*CTH1* #1 or si*CTH2* #2) prominently augmented JPH203-mediated growth inhibition by 33.8% and by 34.5% in MDA-MB-231, respectively (Figs. 4F). In contrast, *GCN2*, *ATF4* or *CTH* knockdown did not affect the growth-inhibitory effect of JPH203 in T-47D cells (Fig. 4B,D,F).

### JPH203-mediated CTH induction mitigated reactive oxygen species (ROS) release caused by amino acid starvation stress

**Figure 5:**
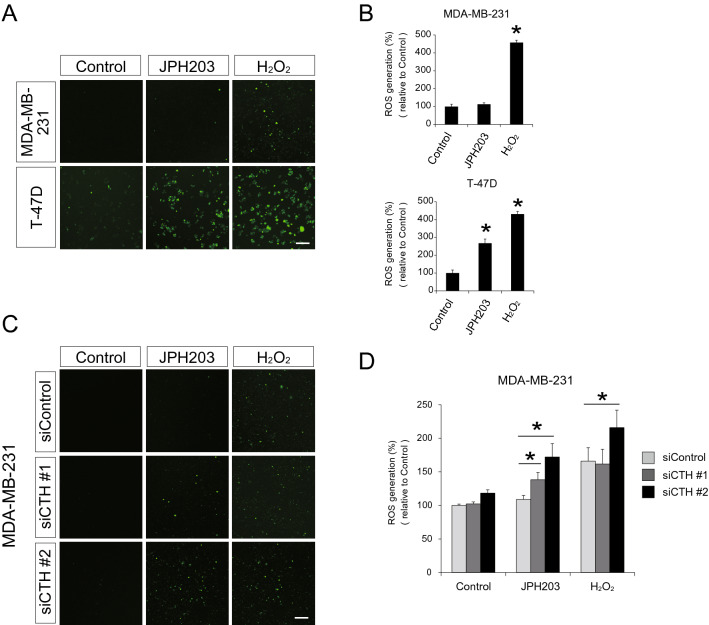
Cellular reactive oxygen spices (ROS) levels in response to JPH203 treatment. (**A**) Cell-permeable fluorogenic dye, DCFH-DA, was used to observe cellular ROS level after treatment with 100 μM JPH203 for 48 h in MDA-MB-231 and T-47D cells. Hydrogen peroxide treatment for 20 min was used as positive control. Scale bar = 100 μm. (**B**) Fluorescence intensity of (**A**) was measured by micro plate-reader. *p < 0.05. (**C**) MDA-MB-231 cells were transfected with control (scrambled) or *CTH* siRNA followed by incubation in the absence or presence of 100 μM JPH203 for 48 h. Cellular ROS levels were measured with DCFA-DA. Hydrogen peroxide treatment for 20 min was used as positive control. Scale bar = 100 μm. (**D**) Fluorescence intensity of (**C**) was measured by micro plate-reader. *p < 0.05.

We next sought to examine whether JPH203-mediated CTH induction attenuated ROS levels derived from amino acid starvation stress. To that end, we employed cell-permeable DCFH-DA, which is a fluorogenic dye to indicate cellular ROS levels, in JPH203-treated cells. ROS production was significantly increased by 2.5-fold in JPH203-treated T-47D cells, while it was not altered in JPH203-treated MDA-MB-231 cells (Fig. 5A,B). Exogenous H_2_O_2_ led to increased ROS levels by 4.5-fold and by 4.2-fold compared with control in both MDA-MB-231 and T-47D cells, respectively.

In *CTH* knockdown MDA-MB-231 cells, JPH203 treatment increased the amount of ROS by 29% (si*CTH* #1) and 63% (si*CTH* #2), respectively (Fig. 5C,D). Thus, CTH upregulation in response to JPH203 treatment is likely to function as enhancing anti-oxidant capacity of MDA-MB-231 cells.

### CTH overexpression supported cell survival under JPH203-induced amino acid starvation in T-47D cells

**Figure 6:**
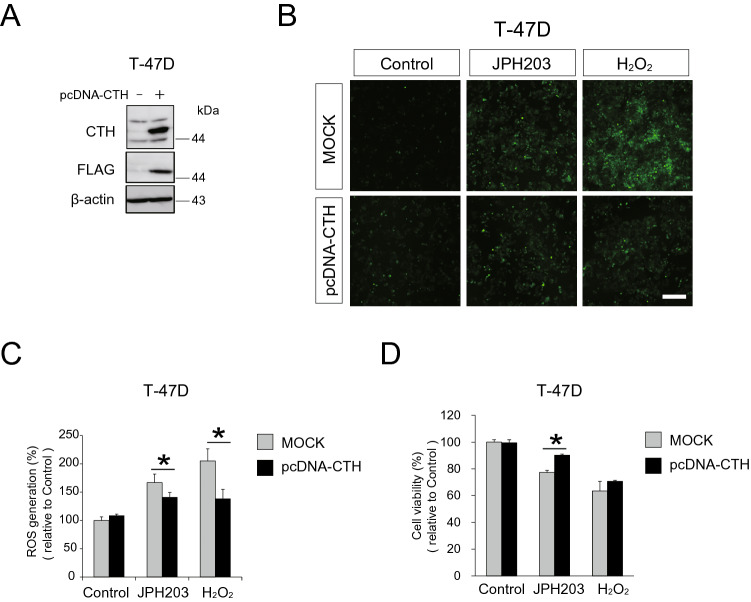
ROS generation and cell viability in response to JPH203 in CTH overexpressed T-47D cells. (**A**) 1 μg of pcDNA3.1-*CTH* vector or mock vector were transiently transfected into T-47D cells for 24 h. CTH expression and FLAG were confirmed by western blot. (**B**) After mock or CTH overexpressed T-47D cells were incubated in the absence or presence of 5 μM JPH203 for 48 h, DCFH-DA was applied to observe generation of ROS. Scale bar = 100 μm. (**C**) Quantification of intracellular ROS level of (**B**) by microplate reader. *p < 0.05. (**D**) Cell viability of mock or CTH overexpressed T-47D after incubation in the absence or presence of 5 μM JPH203 for 48 h was evaluated by MTT assay. *p < 0.05.

To investigate whether CTH overexpression could attenuate an anti-proliferative effect by JPH203, T-47D cells were transiently transfected with pcDNA3.1-*CTH* plasmid (Fig. 6A). In its usual culture condition in the absence of JPH203 or H_2_O_2_, either mock-transfected or *CTH*-transfected T-47D cells did not produce much ROS. Increased ROS levels observed in the presence of JPH203 or H_2_O_2_ were attenuated in CTH overexpressed cells by 25% or by 66% respectively (Fig. 6B,C). CTH overexpression significantly increased T-47D cell viability by 13% in JPH203 and by 8% in H_2_O_2_, respectively (Fig. 6D). Collectively, these findings suggested that increased levels of CTH could reduce ROS levels caused by amino acid deprivation stress and give the cell an ability to survive such a stress.

### MDA-MB-231 cells were primed for anti-oxidant production at baseline

**Figure 7:**
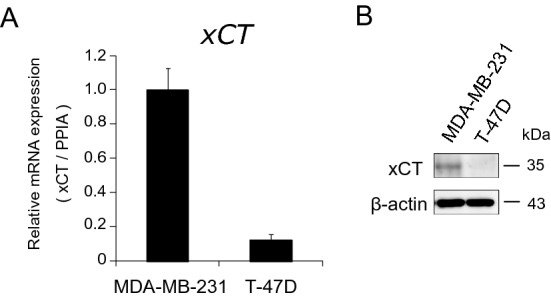
xCT expression in MDA-MB-231 and T-47D cells. (**A**) Non-stimulated basal *xCT* mRNA expression in MDA-MB-231 and T-47D cells by qPCR. (**B**) Non-stimulated basal xCT protein expression in MDA-MB-231 and T-47D cells by western blot analysis.

Noting that expression levels of CTH was higher in MDA-MB-231 cells than in T-47D cells at baseline (Fig. 3B,D), we looked at microarray data for anti-oxidant related molecules. Not only *CTH*, but also cystine/glutamic acid transporter (*xCT*) was expressed more abundantly in MDA-MB-231 cells compared with T-47D cells (Fig. 7). These results suggest that machinery for producing antioxidant GSH and cysteine was more active even at baseline in MDA-MB-231 cells.

## Discussion

In this study, we compared 2 breast cancer cell lines, MDA-MB-231 and T-47D cells. Leucine uptake was almost exclusively mediated by LAT1 in both cells and JPH203 inhibited leucine uptake of these cells at IC50_uptake_ of 0.06 ± 0.02 μM for MDA-MB-231 cells and 0.03 ± 0.01 μM for T-47D cells, respectively (p = 0.07). However, MDA-MB-231 cells exhibited “resistance” to anti-proliferative effect of JPH203; IC50_proliferation_ of 200 ± 12.5 μM for MDA-MB-231 cells and 5 ± 1.1 μM for T-47D cells, respectively (p < 0.05). The discrepancy in IC50 _uptake_ and IC50_proliferation_ for JPH203 has been reported previously, where JPH203’s IC50_uptake_ = 0.06 μM and IC50_proliferation_ = 4.1 μM in colon cancer cell line HT29 cells. They reasoned that there exist much more amino acids in the proliferation assay than in the uptake assay^12^. In addition, the time course for leucine uptake was 2 min while the cell proliferation assay took 4 days in our study. Because JPH203 was not an irreversible inhibitor, it is impossible to completely block substrates uptake all through a long incubation period, especially in the media rich in amino acids and in the presence of other leucine transporters such as LAT3 albeit small amount comparing to LAT1. In line with this, Cormerais et al. found that 90% reduction of LAT1 expression by itself does not lead to growth suppression, suggesting that more than IC90_uptake_ of JPH203 (in our case more than 1 μM in both cells; Fig. 1E) would require to attain significant growth suppression^17^. The proliferation of T-47D cells was effectively suppressed by 5 μM of JPH203, but that of MDA-MB-231 cells was not (Fig. 1F). Upon obtaining these data, we decided to clarify the reason why the proliferation of MDA-MB-231 cells was not suppressed to the similar degree as T-47D cells in the presence of more than IC50_uptake_ concentrations (i.e. more than 1 μM). The difference in the anti-proliferative effect of JPH203 among cancer cell lines has been reported elsewhere, but no underlying mechanism has been identified to date to the best of our knowledge^16,28–30^.

**Figure 8:**
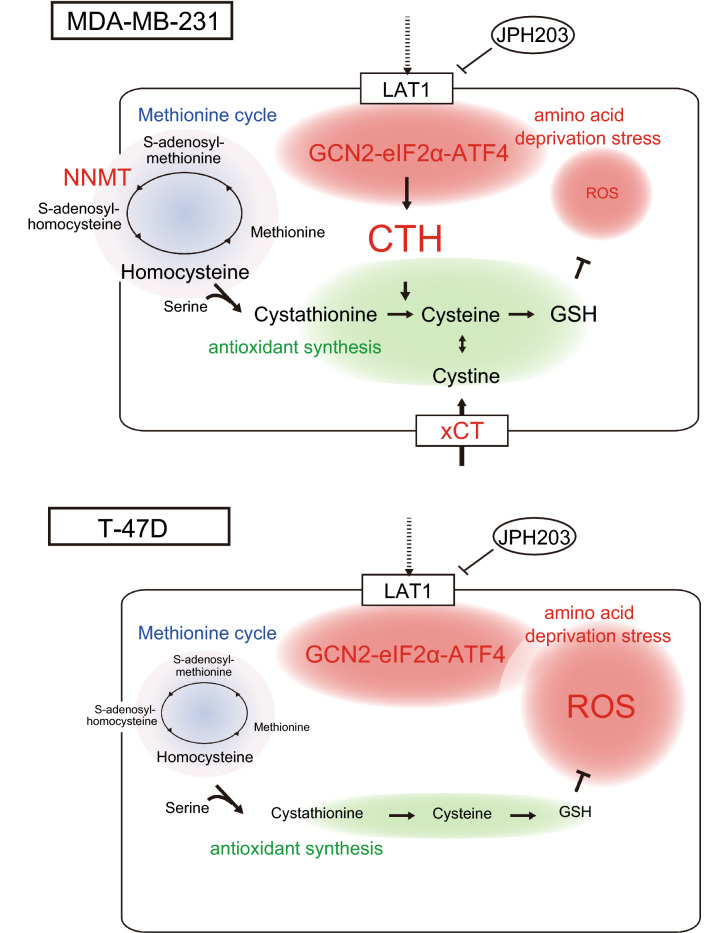
Schematic representation of JPH203-induced amino acid starvation stress pathway in MDA-MB-231 and T-47D cells.

The fact that mTOR-p70S6K pathway was equally suppressed and that a key cellular stress related transcription factor, ATF4, was upregulated in both cells indicates that JPH203 efficiently inhibited essential amino acids uptake into the cells and it induced cellular nutritional stress response to a similar degree between MDA-MB-231 and T-47D cells. Following ATF4 upregulation, these 2 cells upregulated different sets of downstream molecules; anti-oxidant molecules were increased only in MDA-MB-231 cells (Fig. 8). CTH is an enzyme which transforms cystathionine derived from methionine into cysteine and contributes to produce antioxidants such as glutathione and taurine^31^. Another gene upregulated in response to JPH203 was *NNMT*, which catalyzes transmethylation from S-adenosylmethionine to S-adenosylhomocysteine, which can be converted to homocysteine, then to cystathionine, a substrate for CTH^23,32^. When MDA-MB-231 cells were treated with 100 μM of JPH203, their viability was down from ~ 60% of vehicle treatment in control siRNA transfected cells to ~ 30% in *CTH* knockdown cells (Fig. 4F), which were almost the same levels of growth suppression observed with 100 μM of JPH203 treatment in wild type T-47D cells (Fig. 1F). Thus, it is likely that JPH203 resistance in MDA-MB-231 cells are largely mediated by upregulation of CTH in these cells.

Moreover, at baseline, a molecule that increases intracellular cysteine, cystine/glutamic acid transporter (xCT) was more abundantly expressed in MDA-MB-231 cells than T-47D cells (Fig. 7)^33^. Taken together, both at baseline and in response to JPH203, MDA-MB-231 cells appeared to be more prepared to handle ROS load, thereby better survive metabolic stress caused by amino acid depletion than T-47D cells. Given the fact that increased ROS load is the mechanism of inducing cell death in cytotoxic cancer chemotherapy or radiation therapy^34^, it is tempting to speculate that increased anti-oxidative capacity in MDA-MB-231 cells may explain, at least partially, their resistance to classic cancer therapies in addition to JPH203. Whether these findings are generally applicable to triple negative breast cancers is an interesting subject for future research. Regardless, as stated above, upregulation of CTH is likely to be the main mechanism of resistance to JPH203 treatment at least in MDA-MB-231 cells.

To address the question whether increased antioxidant capacity observed in MDA-MB-231 cells in response to JPH203 is due to amino acid depletion or unknown side effect of JPH203, the cells were cultured in the medium depleted of LAT1 substrate amino acids, essential amino acids plus tyrosine (Fig. 3D). This treatment induced upregulation of CTH in MDA-MB-231 cells as seen in JPH203 treatment. Even T-47D cells, which did not increase CTH in the presence of JPH203, slightly increased CTH when substrate amino acids were totally depleted. However, complete depletion of essential amino acids plus tyrosine was impossible with even more than 100 μM of JPH203, because JPH203 is a reversible competitive inhibitor in a culture condition rich in substrate amino acids for much longer periods (4 days) than uptake assay (2 min). In addition, both of these cells expressed small amount of essential amino acid transporters that were not blocked by JPH203 (Fig. 1A).

Another result showing that treatment with more than 5 μM (up to 200 μM) of JPH203 did not increase CTH in T-47D cells (Fig. 3E) speaks against direct (i.e. not mediated by amino acid depletion) upregulation of CTH by JPH203. Taken together, it is likely that increased anti-oxidant capacity in MDA-MB-231 cells in response to JPH203 treatment was due to amino acid depletion, not a side effect of JPH203. In this regard, Cormerais et al.^17^ reported that amino acid stress response of ATF4 upregulation and CGN2 phosphorylation is observed in *LAT1* knockout cells. Both T-47D and MDA-MB-231 cells in our study showed the same stress response by JPH203 treatment (Fig. 3B). These results further support our interpretation that JPH203 induced cellular stress response in MDA-MB-231 cells by restricting amino acid uptake through LAT1.

Investigators including us often regard that inhibition of LAT1 causes tumor cell growth suppression through mTORC1 pathway inhibition or simply lack of essential amino acids, but this study implied that cellular anti-oxidant pathways in response to amino acid deprivation stress may be at least as important for tumor cell viability.

Although *ATF4* knockdown abolished CTH upregulation in MDA-MB-231 cells, it is not clear how *CTH* transcription was differently regulated between these 2 cells downstream of ATF4. Moreover, because we only compared 2 breast cancer cell lines, generalizing our findings to wide variety of cancer cells should be done with caution.

## Materials and methods

### Reagent

JPH203 ((S)-2-amino-3-(4-((5-amino-2-phenylbenzo[d]oxazol-7-yl)methoxy)-3,5-dichlorophenyl)-propanoic acid), was kindly provided by J-Pharma Co., Ltd.

### Cell culture

MDA-MB-231 and T-47D cells were purchased from American Type culture collection (ATCC, Manassas, VA, USA) and cultured in RPMI1640 medium. (nacalai tesque, Kyoto, Japan) supplemented with 10% fetal bovine serum (Biowest, VB, FRA), 100 units/mL penicillin, and 100 mg/mL streptomycin at 37 °C with 5% CO_2_.

### Immunocytochemistry

Cells were seeded on glass coverslips in 12-well plate at a density of 1 × 10^5^ cells/well. After 2-day culture, cells were fixed in cold methanol, permeabilized in buffer (0.1% BSA, 0.3% Triton X-100 in PBS), and blocked with goat serum dilution buffer (10% goat serum, 1% Triton X-100, 10 mM glycine in PBS, GSDB). Slides were incubated with LAT1 (1:100, Trans Genic Inc., Fukuoka, Japan) or 4F2hc (1:100, Santa Cruz Biotechnology, Dallas, TX, USA) diluted in GSDB buffer overnight. Cells were incubated with Alexa Fluor 594 conjugated anti-mouse IgG (1:100 Life Technologies, Carlsbad, CA, USA) and DAPI (1:500; Roche, Basel, Switzerland) diluted in GSDB buffer, and then mounted in fluoro-KEEPER Antifade Reagent (nacalai tesque, Kyoto, Japan). Fluoro-images were captured by Fluoview FV500 Laser confocal microscope (Olympus, Tokyo, Japan).

### RNA extraction and real-time quantitative PCR analysis

Cells were plated on 60 mm dishes at a density of 4 × 10^5^ cells/dish and cultured for 2 days. Cells were subsequently treated with 100 μM JPH203 for 12 h before harvesting total RNA. Total RNA was isolated using Isogen (Nippon Gene, Tokyo, Japan) according to the manufacturer's instruction. The first-strand complementary DNA (cDNA) were synthesized from 1 μg of total RNA using MuLV Reverse Transcriptase (Life Technologies, Carlsbad, CA, USA) with oligo dT primer. Real-time PCR was performed with Premix Ex Taq (Takara Bio Inc., Shiga, Japan) or SYBR Select Master Mix (Thermo Fisher Scientific, MA, USA) using 7300 Real-Time PCR system (Thermo Fisher Scientific, MA, USA). Designed primers and probes were shown in Table 3.

**Table 3:** Primer pairs and probes for real-time PCR.

Gene		Primer pairs and probes
*LAT1*	Forward	5′-GGA AGG GTG ATG TGT CCA ATC T-3′
	Reverse	5′-TTC AAG TAA TTC CAT CCT CCA TAG G-3′
	Probe	5′-FAM-CCC AAC TTC TCA TTT GAA GGC ACC AAA CT-TAMRA-3′
*LAT2*	Forward	5′-AAA TCT GGA GGT GAC TAC TCC TAT GTC-3′
	Reverse	5′-GTA GAT CAC CAG CAC AGC AAT CC-3′
	Probe	5′-FAM-TCT TCG GAG GAC TGG CTG GGT TCC-TAMRA-3′
*LAT3*	Forward	5′-CCC CAA CTC AGG GCA CTG T-3′
	Reverse	5′-GTA GCG TGG TCT GAT GGA TTT G-3′
	Probe	5′-FAM-CTC GGA GAT GCC AGG GAC GGG-TAMRA-3′
*LAT4*	Forward	5′-GCC CCT GGG TAT CGT CAT G-3′
	Reverse	5′-CGT ACG CAA TCA GCA AGC A-3′
	Probe	5′-FAM-CAG CGC CTG CTT CGC GGT TT-TAMRA-3′
*y* + *LAT1*	Forward	5′-GCC AAC TAC ATG GTA CAG CCT CTC-3′
	Reverse	5′-TGA AGG TTA AGA GAC AAA TGC AGG CA-3′
	Probe	5′-FAM- CCC TTA TGC TGC CAG CCG CCT GCT-TAMRA-3′
*y* + *LAT2*	Forward	5′-TTC AGA TGT CCT TAG CAG TGA TGC-3′
	Reverse	5′-CGA AGA ACA ACC TTG ATG AAG CAA AG-3′
	Probe	5′-FAM- CCC AAA GCA GGA CAG GGC AAC AGC AA-TAMRA-3′
*ATB(0*^+^*)*	Forward	5′-TCA ACA ATT TTA CCT GCA TCA ACG G-3′
	Reverse	5′-GTT GGA GCG CCA CTT TAT TCC AA-3′
	Probe	5′-FAM- AGC CAG GGC AGC TTC CCA GTG AAC AA-TAMRA-3′
*ATF4*	Forward	5′-ACA GCA AGG AGG ATG CCT TC-3′
	Reverse	5′-CAA CGT GGT CAG AAG GTC ATC-3′
*CTH*	Forward	5′-CAT GAG TTG GTG AAG CGT CAG-3′
	Reverse	5′-AGC TCT CGG CCA GAG TAA ATA-3′
*xCT*	Forward	5′-GCT TTC AAA TGC AGT GGC AGT-3′
	Reverse	5′-AGC AAA CAC ACC ACC GTT CA-3′
*CHAC1*	Forward	5′-CAG GGA GAC ACC TTC CAT CG-3′
	Reverse	5′-GGT ACT TCA GGG CCT TGC TT-3′
*PPIA*	Forward	5′-TGG TTC CCA GTT TTT CAT CTG C-3′
	Reverse	5′-CCA TGG CCT CCA CAA TAT TCA-3′

### Protein extraction and Western blot analysis

Cells were seeded in 6-well plates at a density of 2 × 10^5^ cells/well and cultured for 2 days. Cells were subsequently treated with 0, 0.05, 5, 100 or 200 µM JPH203 for 12 or 48 h as indicated in each figure legend, and dissolved in Lysis buffer (150 mM NaCl, 0.5 mM EDTA, 1% Triton X-100 and 50 mM Tris–HCl, pH 7.4) with cOmplete Mini (Roche, Basel, Switzerland) and PhosSTOP, phosphatase inhibitor (Roche, Basel, Switzerland). 10 μg of extracted protein was subjected to SDS-PAGE and transferred to Immobilon-P PVDF membrane (Millipore, KGaA, Darmstadt, Germany). The membrane was blocked with Bullet Blocking One (nacalai tesque, Kyoto, Japan) and incubated with anti-LAT1 (1:1000; Trans Genic Inc., Fukuoka, Japan), anti-4F2hc (1:2000; Santa Cruz Biotechnology, Dallas, TX, USA), anti-phospho-p70S6K (1:1000), anti-p70S6K (1:1000), anti-ATF4 (1:1500), anti-phospho-GCN2 (1:1000), anti-GCN2 (1:1000), anti-phospho-EIF2α (1:1000), anti-EIF2α (1:1000), anti-CTH (1:1500; all from Cell Signaling Technology, Danvers, TX, USA), anti-xCT (1:1000, Abcam, Cambridge, UK), and anti-β-actin (1:4000; Sigma-Aldrich, St. Louis, MO, USA) antibodies overnight at 4 °C. After washing, the membrane was incubated with horseradish peroxidase conjugated anti-rabbit IgG or anti-mouse IgG (Jackson Immuno Research Laboratories, West Grove, PA, USA) diluted with Tris buffered saline with Tween 20. Images were obtained using LAS-4000 mini (Fujifilm, Tokyo, Japan). Western blots presented in figures were representative results from three independent experiments.

### [^14^C] **l**-leucine uptake assay

Cells were seeded on 24-well plates at a density of 1 × 10^5^ cells/well for 2 days before experiment. Cells were subsequently incubated with Na^+^ containing buffer (125 mM NaCl, 4.8 mM KCl, 1.3 mM CaCl_2_, 1.2 mM MgSO_4_, 25 mM HEPES, 1.2 mM KH_2_PO_4_ and 5.6 mM glucose, pH 7.4) or Na^+^-free buffer (125 mM choline chloride, 4.8 mM KCl, 1.3 mM CaCl_2_, 1.2 mM MgSO_4_, 25 mM HEPES, 1.2 mM KH_2_PO_4_ and 5.6 mM glucose, pH 7.4) containing 19 µM leucine, 1 µM [^14^C] l-leucine (> 8.14 GBq/mmol, Moravek, CA, USA), and 0.01–100 μM JPH203 for 2 min at 37 °C. The cells were solubilized with 0.1 N NaOH and cell lysates were added to OptiPhase SuperMix (PerkinElmer, Waltham, MA, USA). The radioactivity was measured by a liquid scintillation counter; LSC-7400 (Hitachi-Aloka Medical, Tokyo, Japan).

### Cell proliferation/viability assay

Cells were precultured on 24-well plates at a density of 5 × 10^4^ cells/well for 24 h and then treated with 0.01–1000 μM JPH203 for 4 days or 48 h. MTT (3-(4,5-dimethylthiazol-2yl)-2, 5-diphenyltetrazolium bromide) (nacalai tesque, Kyoto, Japan) colorimetric assay was conducted on these cells. The 50% inhibitory concentration (IC50) of JPH203 was obtained from the dose–response curve.

### DNA microarray analysis

Cells were seeded on 60 mm dishes at a density of 1 × 10^5^ cells and treated with 100 μM JPH203 for 24 h. Total RNA was extracted with ISOGEN according to the manufacturer’s instruction. DNA microarray analysis was performed using U133 plus 2.0 DNA chip microarray (Affymetrix CA, USA). Data are available at the Gene Expression Omnibus (GEO) under accession: GSE173698.

### Amino acid restriction culture

Cells were seeded on 6-well plates at a density of 1 × 10^5^ cells/well and cultured for 24 h in RPMI1640 medium supplemented with 10% fetal bovine serum, 100 units/mL penicillin, and 100 units/mL streptomycin. Before amino acid restriction, cells were washed thoroughly with 2 mL PBS 3 times, and then cultured in RPMI1640-based amino acid restriction culture medium supplemented with small molecule-free dialyzed FBS (Thermo Fisher Scientific, MA, USA), 100 units/mL penicillin, 100 mg/mL streptomycin, and vitamin solution for RPMI1640 (Sigma-Aldrich, St. Louis, MO, USA) for 72 h. Cells were harvested by Lysis buffer with cOmplete Mini and PhosSTOP, phosphatase inhibitor described in “Protein extraction and Western blot analysis” section. The composition of RPMI1640-based amino acid restriction culture media was shown in Supplementary Table S1.

### siRNA-mediated knockdown

Cells were seeded on 24-well plates at a density of 5 × 10^4^ cells/well, and precultured in RPMI1640 medium supplemented with 10% FBS without antibiotics for 24 h before siRNA transfection. Cells were transfected with two independent Silencer select siRNA oligos; si*ATF4* (#1 = s1702, #2 = s1703), si*GCN2* (#1 = s54067, #2 = s54068), si*CTH* (#1 = s3710, #2 = s3712 all from Thermo Fisher Scientific, MA, USA), si*Control* #1(ON-TARGETplus Non-targeting siRNA #2; Dharmacon, Chicago, IL, USA) using Lipofectamine RNAi max Reagent (Thermo Fisher Scientific, MA, USA), and incubated for 8 h. These cells were incubated in the presence or absence of JPH203 for 48 h and their proliferation/viability was assessed by MTT assay. 100 or 5 μM of JPH203 was added to MDA-MB-231 or T-47D cells, respectively.

### Intracellular ROS detection

Cells were plated on 24-well plates at a density of 7.5 × 10^4^ cells/well, precultured for 24 h, and treated with 100 µM JPH203 for 48 h. Cells were washed 3 times with PBS, and treated with 10 µM 2, 7-Dichlorodihydrofluorescein-diacetate (DCFH-DA) (Sigma-Aldrich, St. Louis, MO, USA) for 30 min. Fluorescence intensity was measured using microplate-reader, and photographed by fluorescence microscope. For a positive control of ROS generation, cells were treated with 500 µM H_2_O_2_ for 20 min after DCFH-DA loading.

### pcDNA3.1-CTH plasmid construction

The PCR products of *CTH* fused with 3 × FLAG tags were cloned into the pcDNA3.1(−) vector and transformed into *E. coli* JM109. Sequences of primer sets were shown as follows: *CTH* forward; 5′-CCG GAA TTC ATG CAG GAA AAA GAC GCC TC-3′, *CTH* reverse; 5′-GCC GGT ACC CTA GCT GTG ACT TCC ACT TG-3′. T-47D cells were seeded on 24 well plates at a density of 7.5 × 10^4^ cells/well for 24 h, and transfected with 1 μg of cDNA3.1-*CTH* or pcDNA3.1 (MOCK) for 24 h using Lipofectamine 3000 (Thermo Fisher Scientific, MA, USA) according to the manufacturer’s instruction.

### Statistical analysis

Experiments were repeated 3 times and the data are expressed as mean ± standard error. Statistical analysis was performed using Student’s t -test (unpaired, two-tailed) and p-value of 0.05 was chosen as the threshold for statistical significance.

### Original blot data

As per the policy of the journal, original blot images of composite figures were presented in Supplementary Fig. 2.

## Supplementary Information

Supplementary Figures.

Supplementary Table S1.
