# Supplementary material for: Induction of CTH expression in response to amino acid starvation confers resistance to anti-LAT1 therapy in MDA-MB-231 cells
Source: Sci Rep. 2022 Jan 19;12:1021. doi: 10.1038/s41598-022-04987-5 (PMC8770514; doi:10.1038/s41598-022-04987-5)

Supplementary materials for

Induction of CTH expression in response to amino acid starvation confers resistance to anti-LAT1 therapy in MDA-MB-231 cells

Takashi Yamaga, Junichi Suehiro, Youichiro Wada, Hiroyuki Sakurai

Supplementary Figure 1 (P2)

Expression of CHAC1 in MDA-MB-231 and T-47D cells in the presence or absence of JPH203. qPCR data was normalized to PPIA.

Supplementary Figure 2 (P3-41)

Original images of western blots used in main figures. Cropped parts are designated by black rectangles.

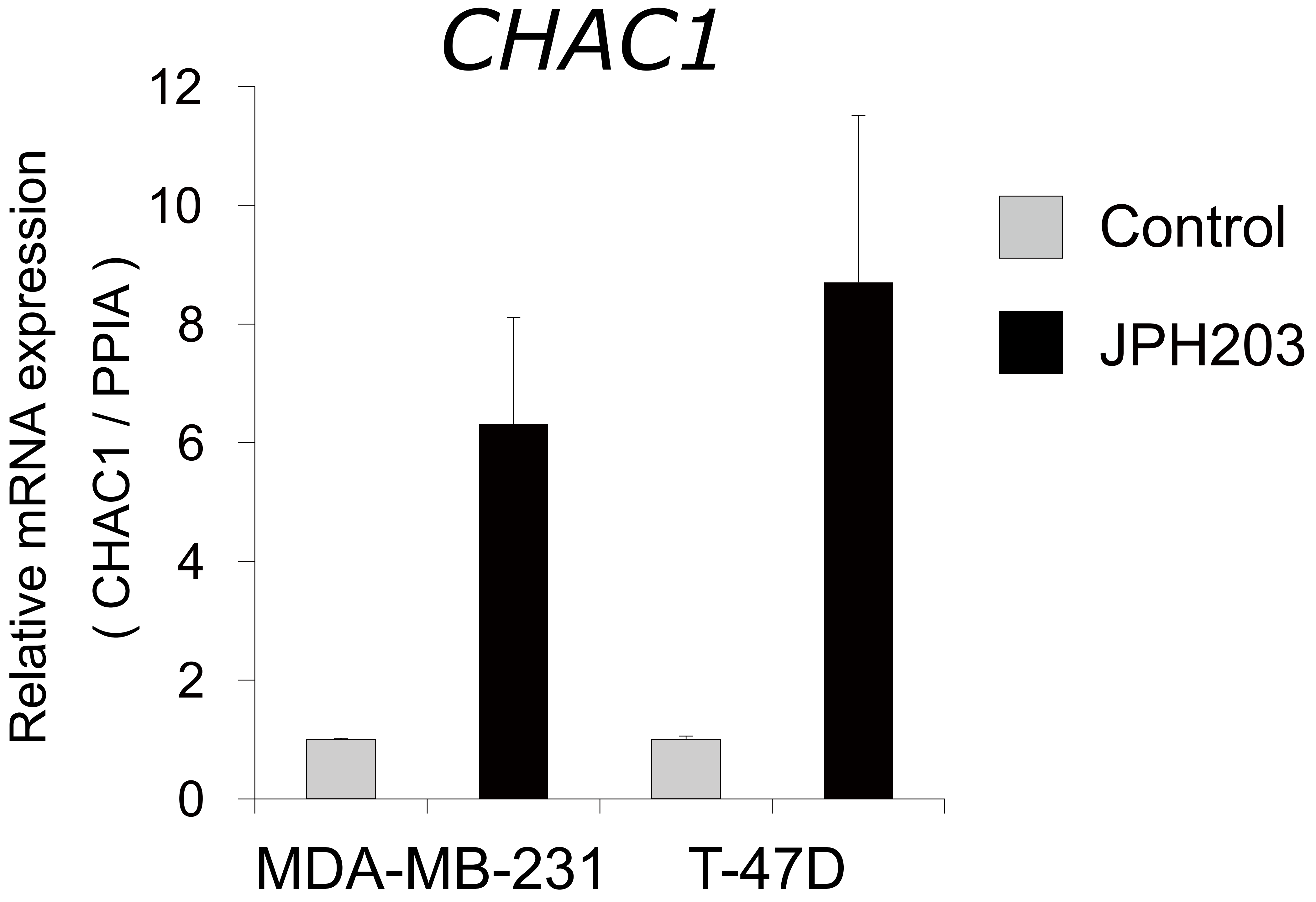

Supplementary Figure 1

Figure 1C (β-actin)

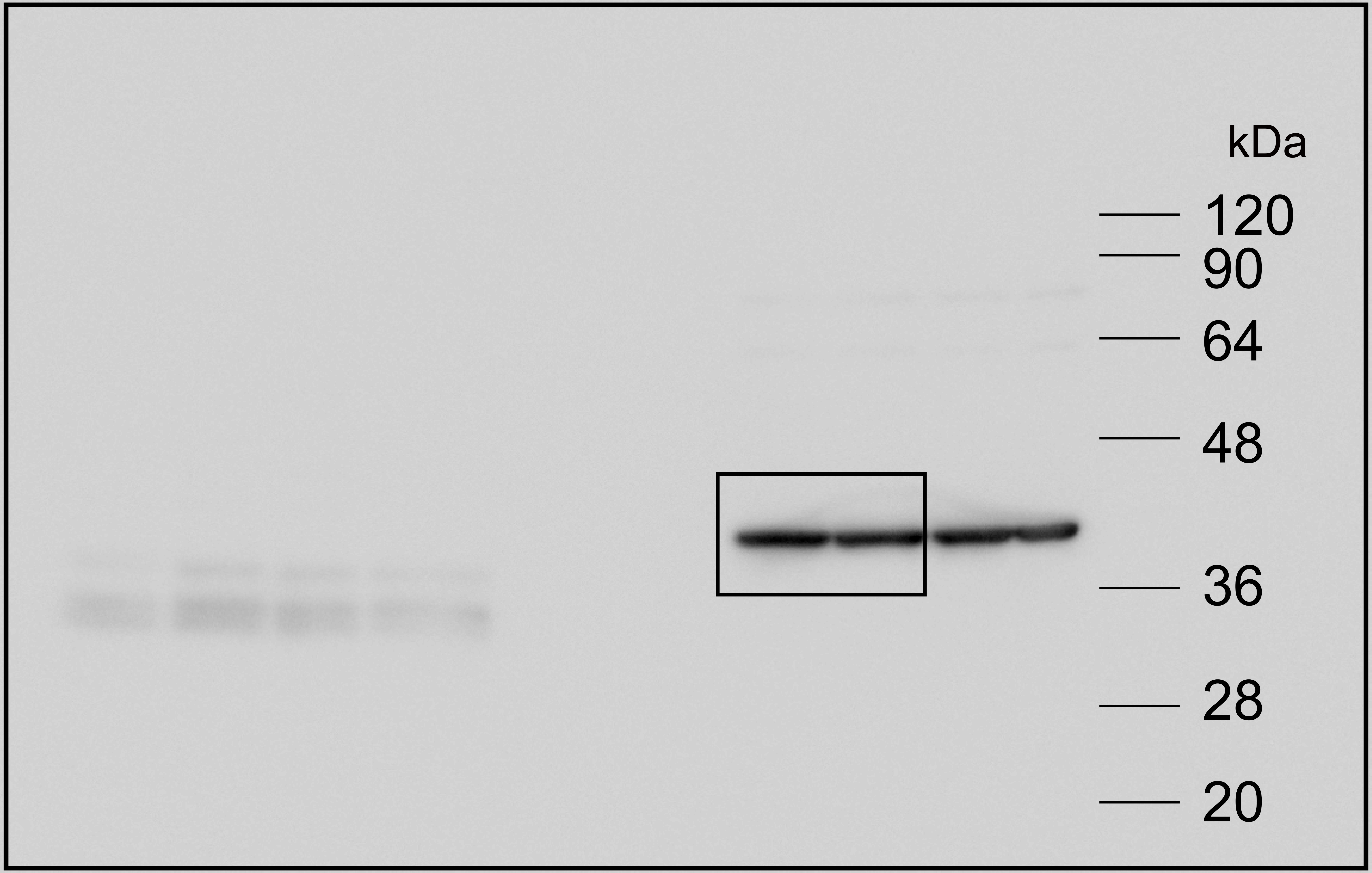

Figure 1C (LAT1)

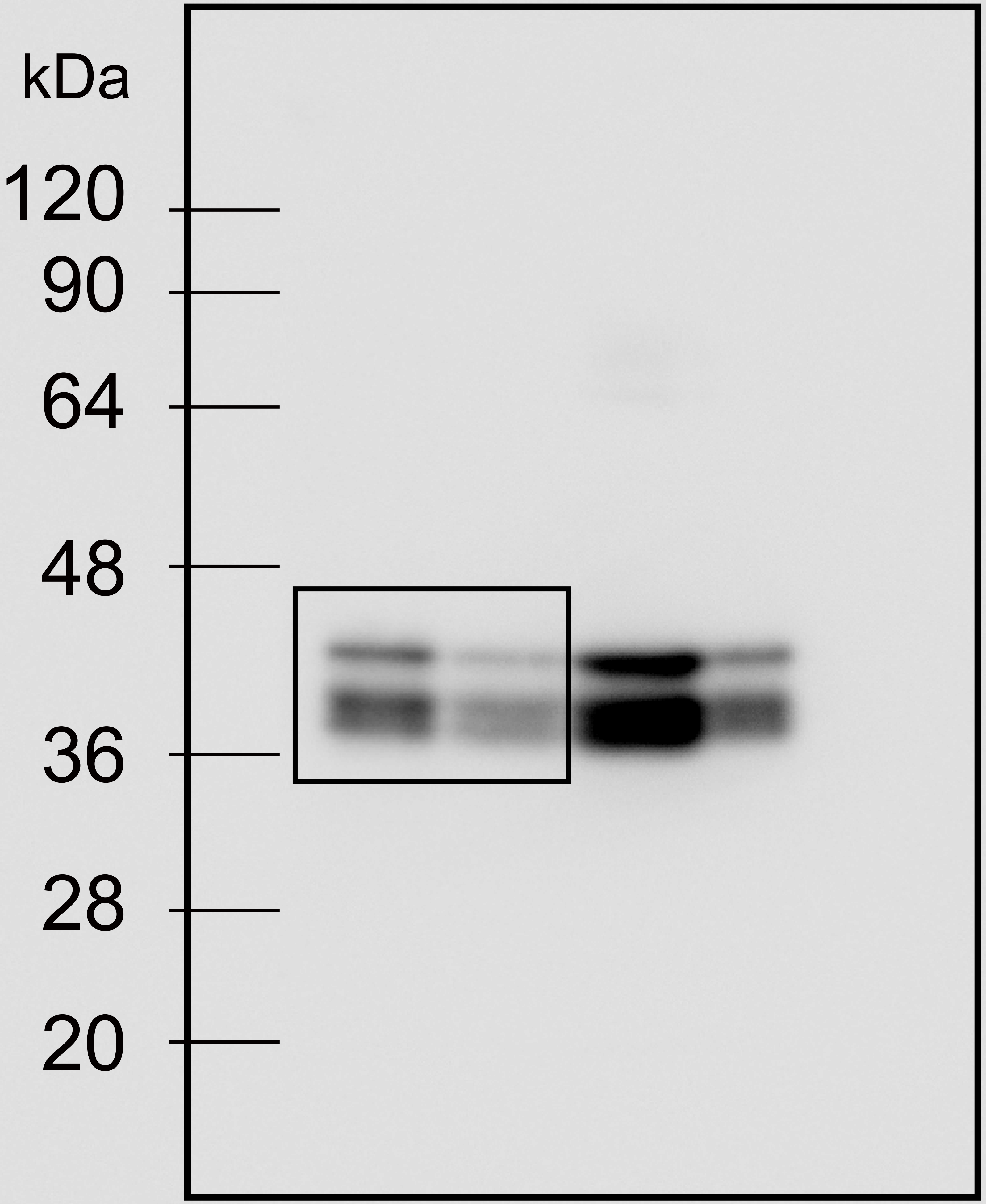

Figure 1C (4F2hc)

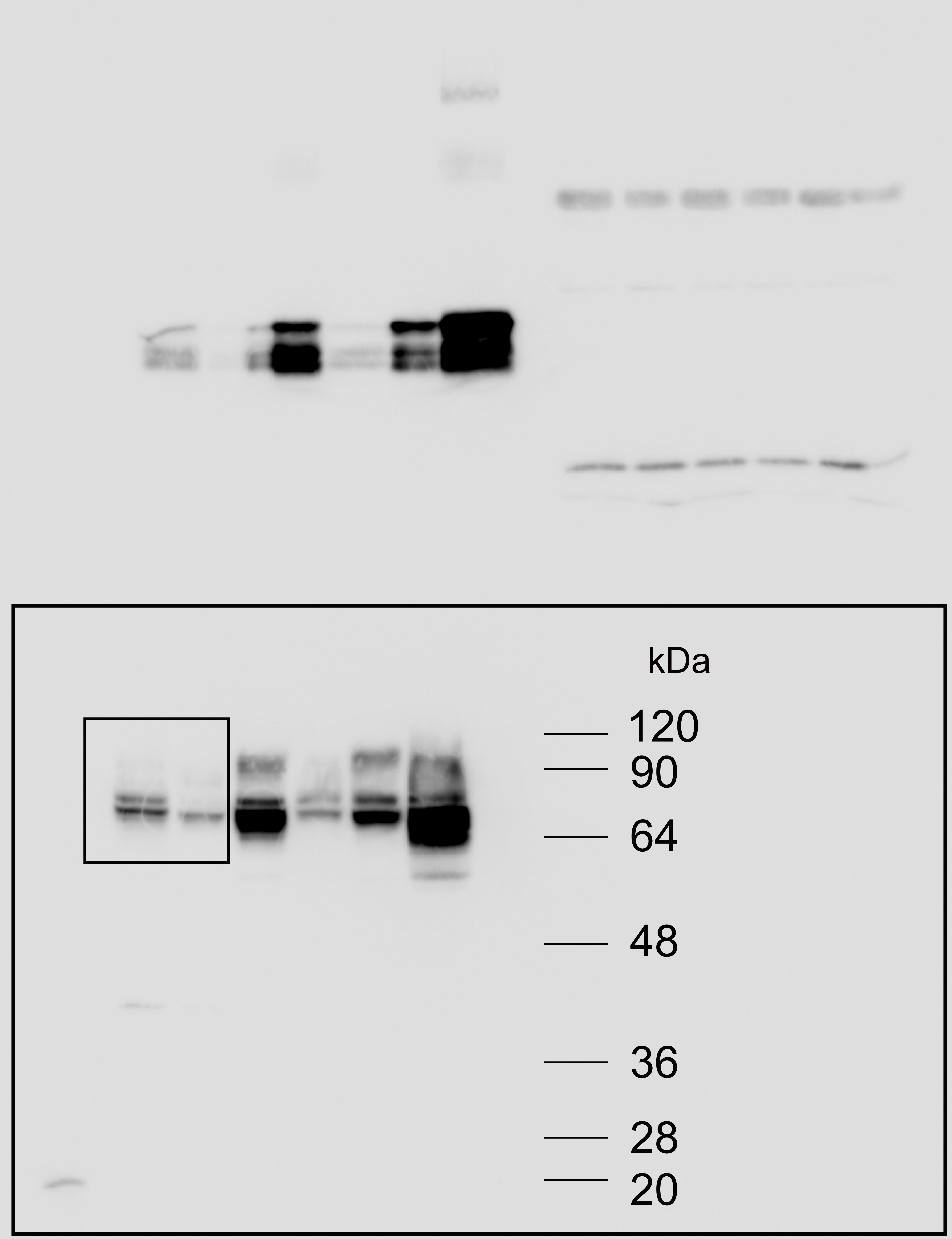

Figure 1G (β-actin)
Left: MDA-MB-231
Right: T-47D

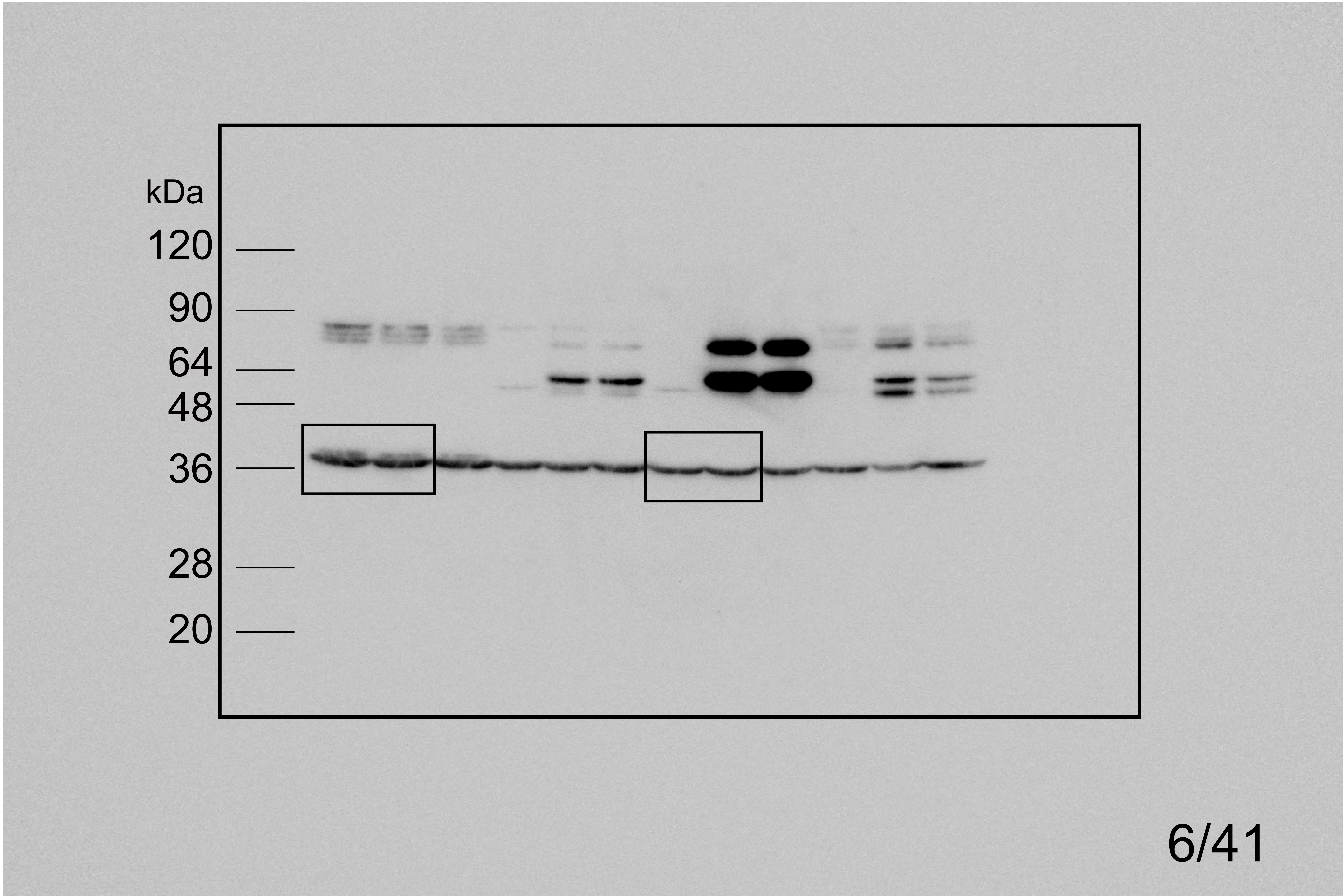

Figure 1G (p70S6K)
Left: MDA-MB-231
Right: T-47D

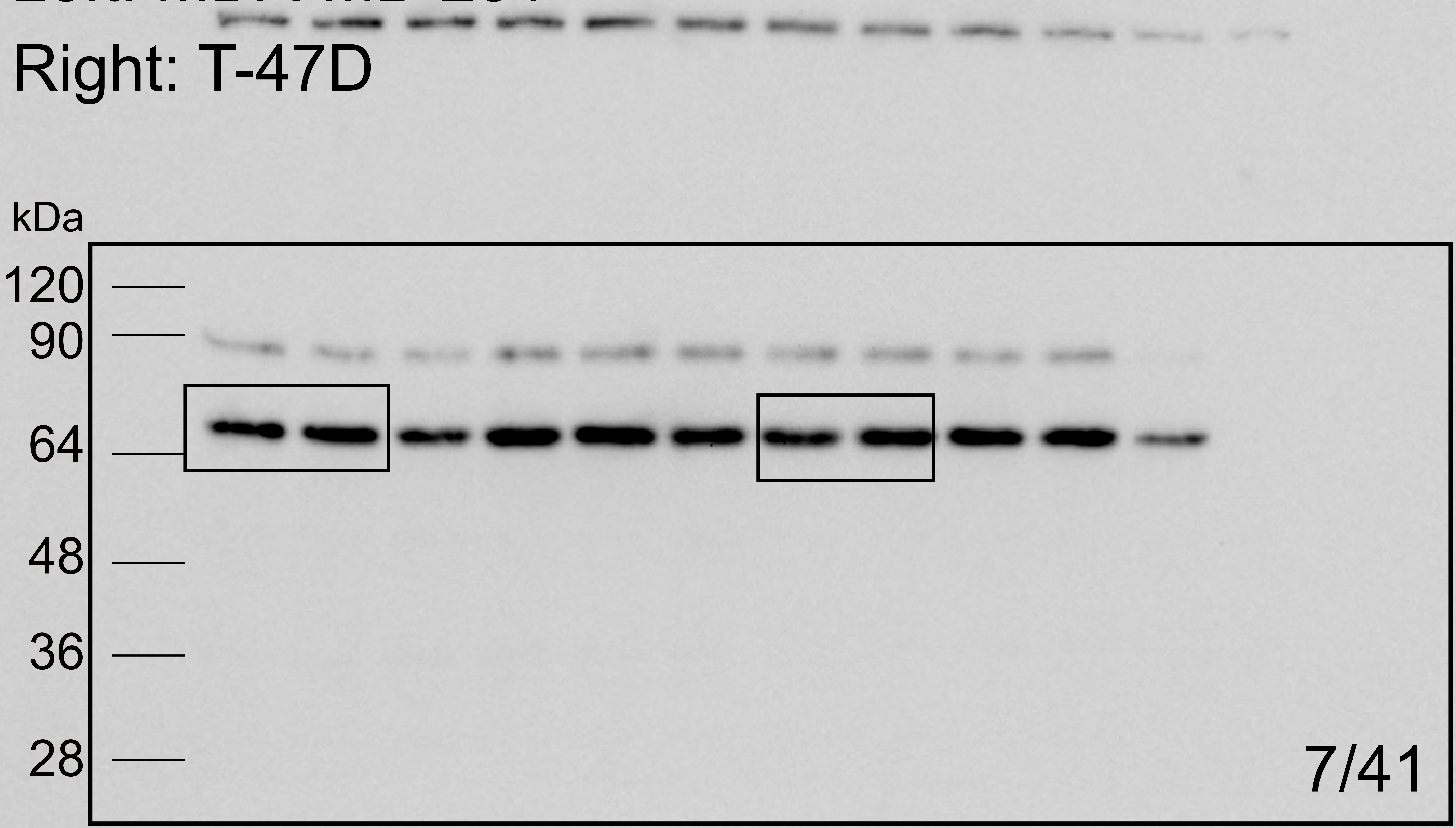

Figure 1G (p-p70S6K)
Left: MDA-MB-231
Right: T-47D

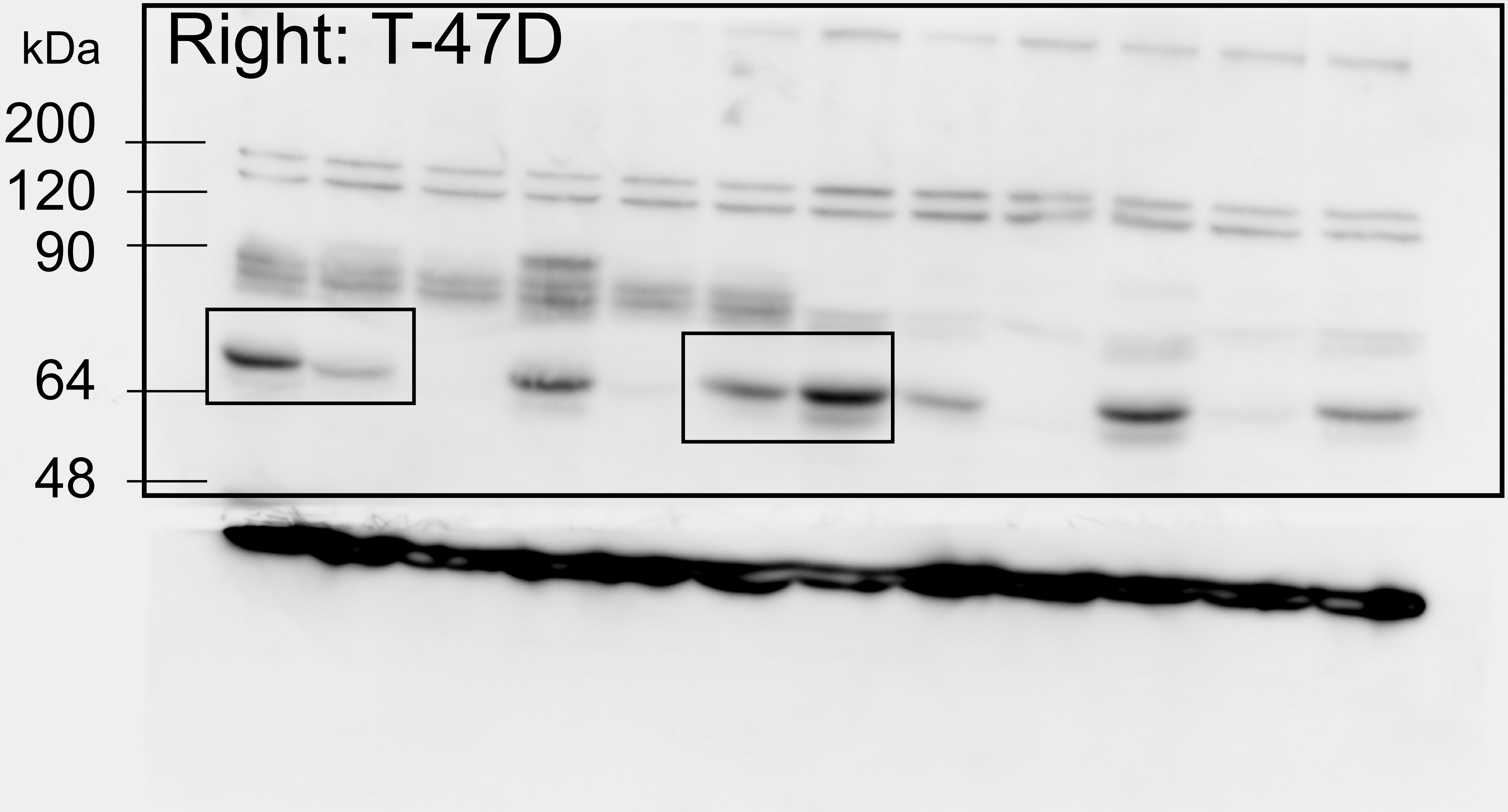

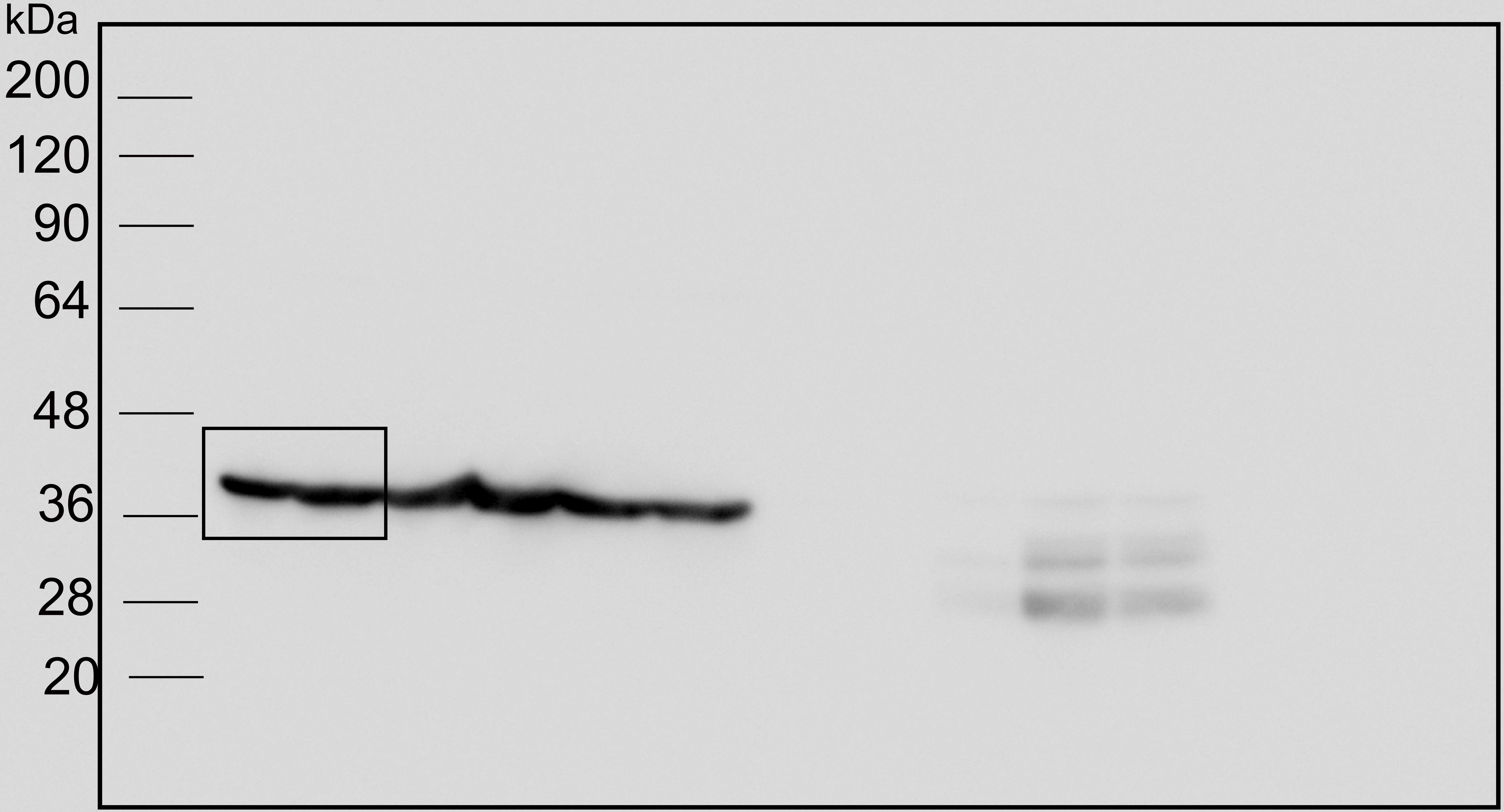

Figure 3B (β-actin)
MDA-MB-231

Figure 3B (β-actin)

T-47D

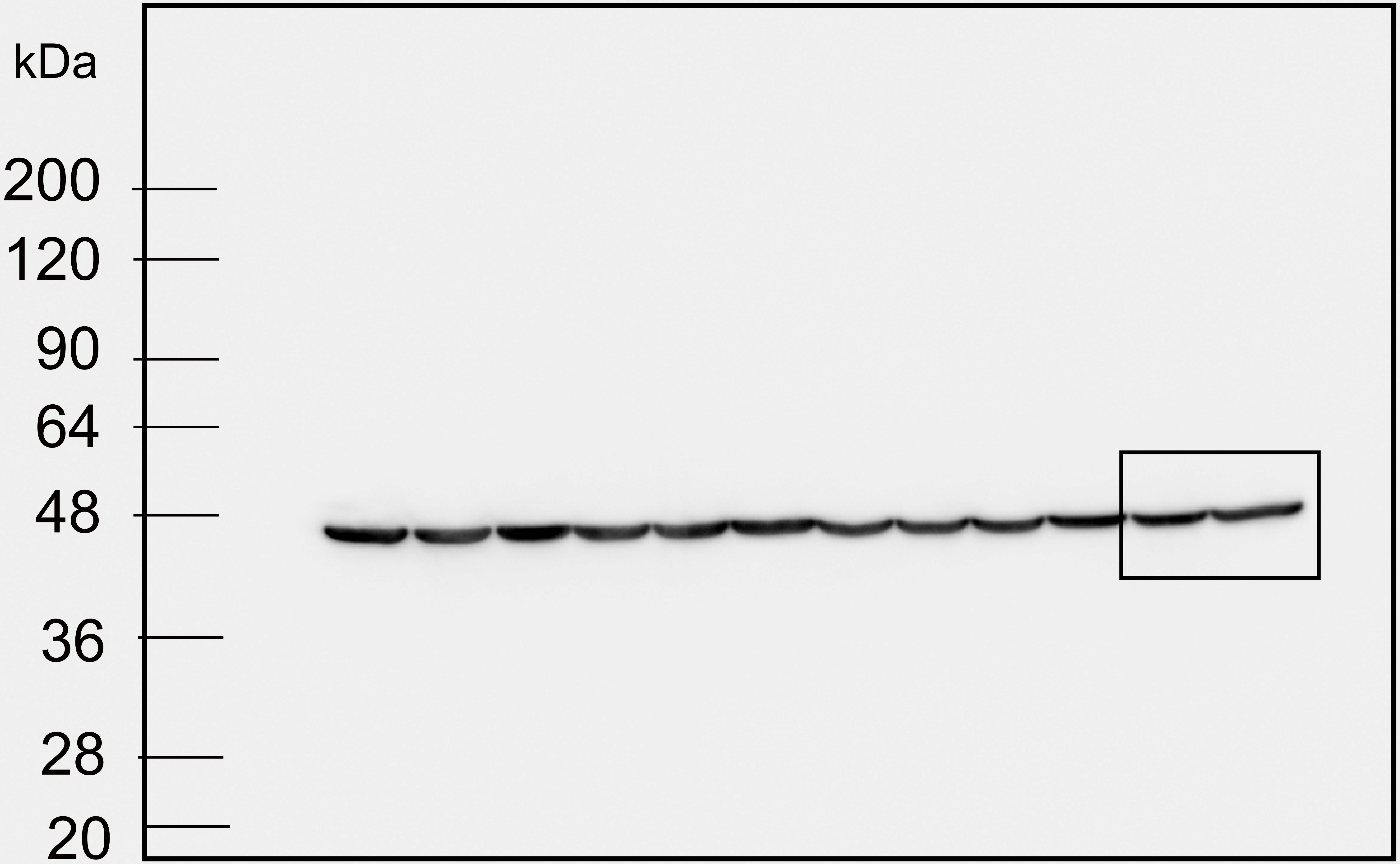

Figure 3B (ATF4)

MDA-MB-231 short exposure

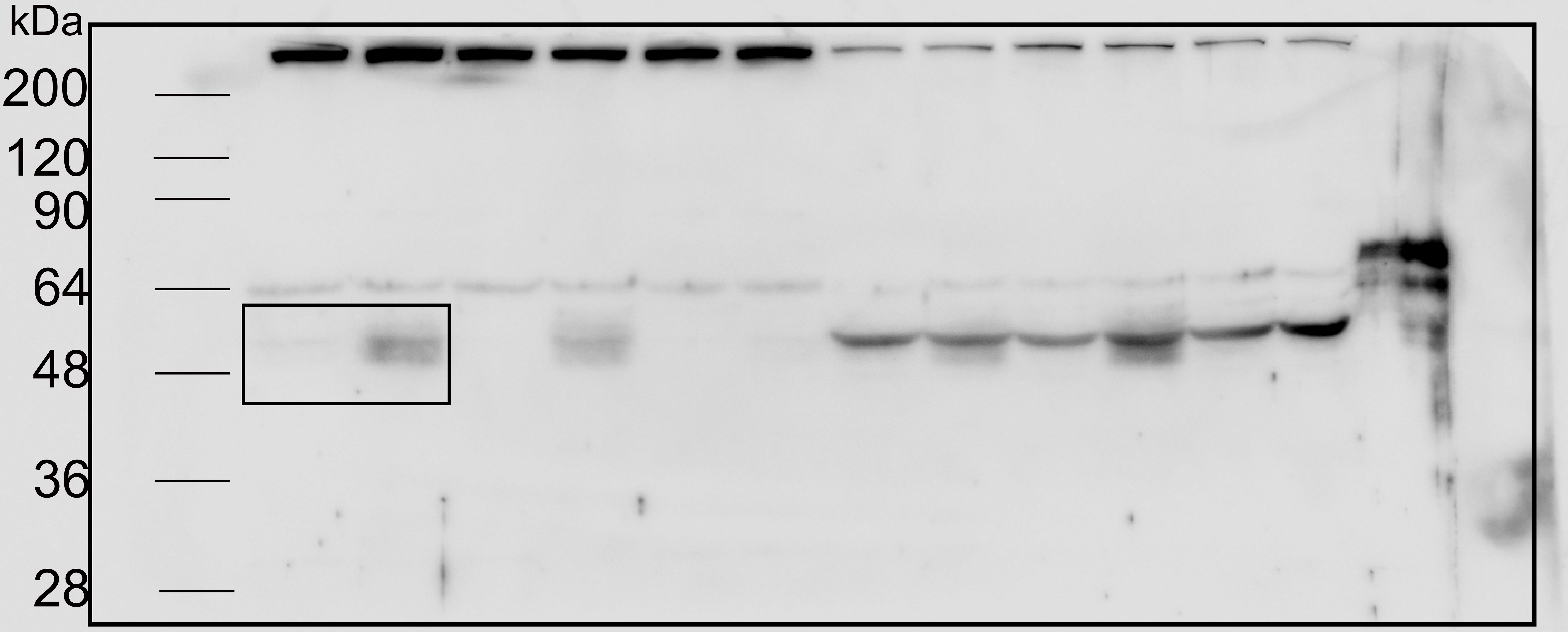

Figure 3B (ATF4)

MDA-MB-231 long exposure

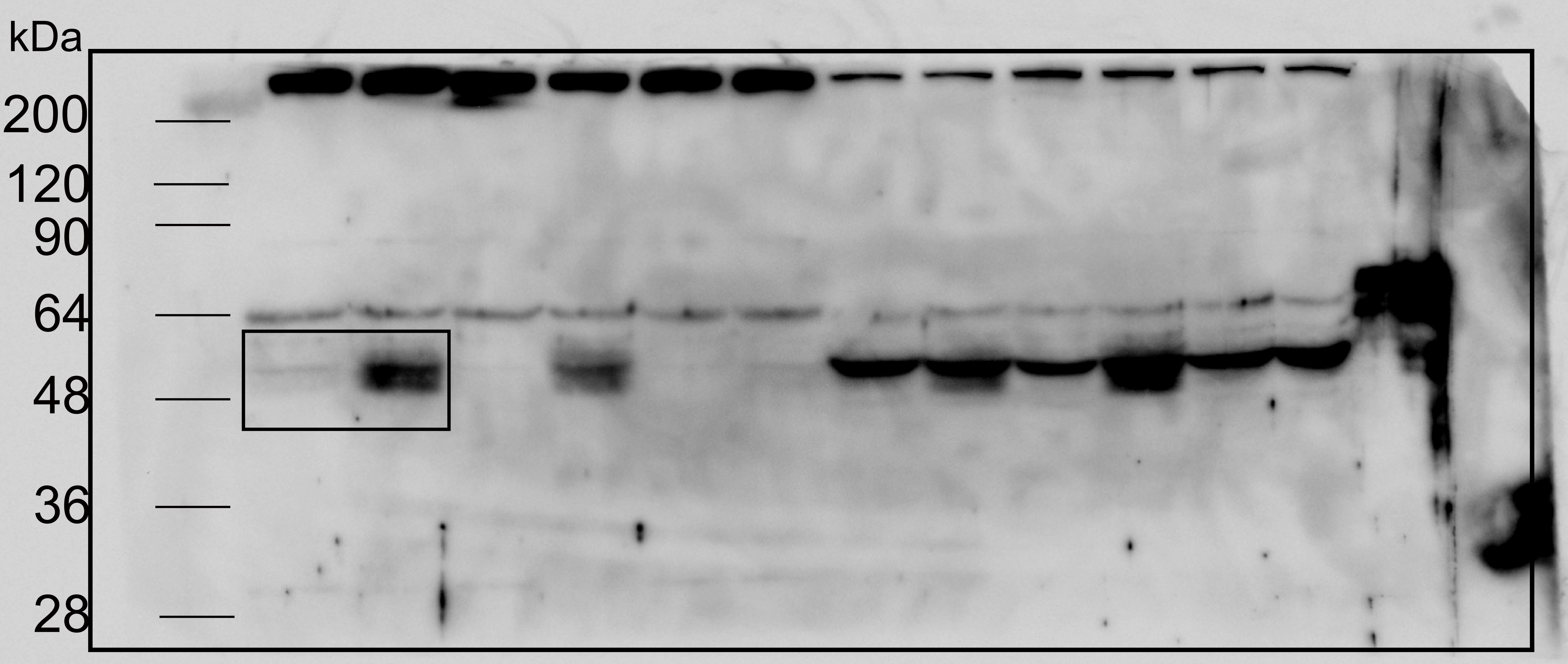

Figure 3B (ATF4)

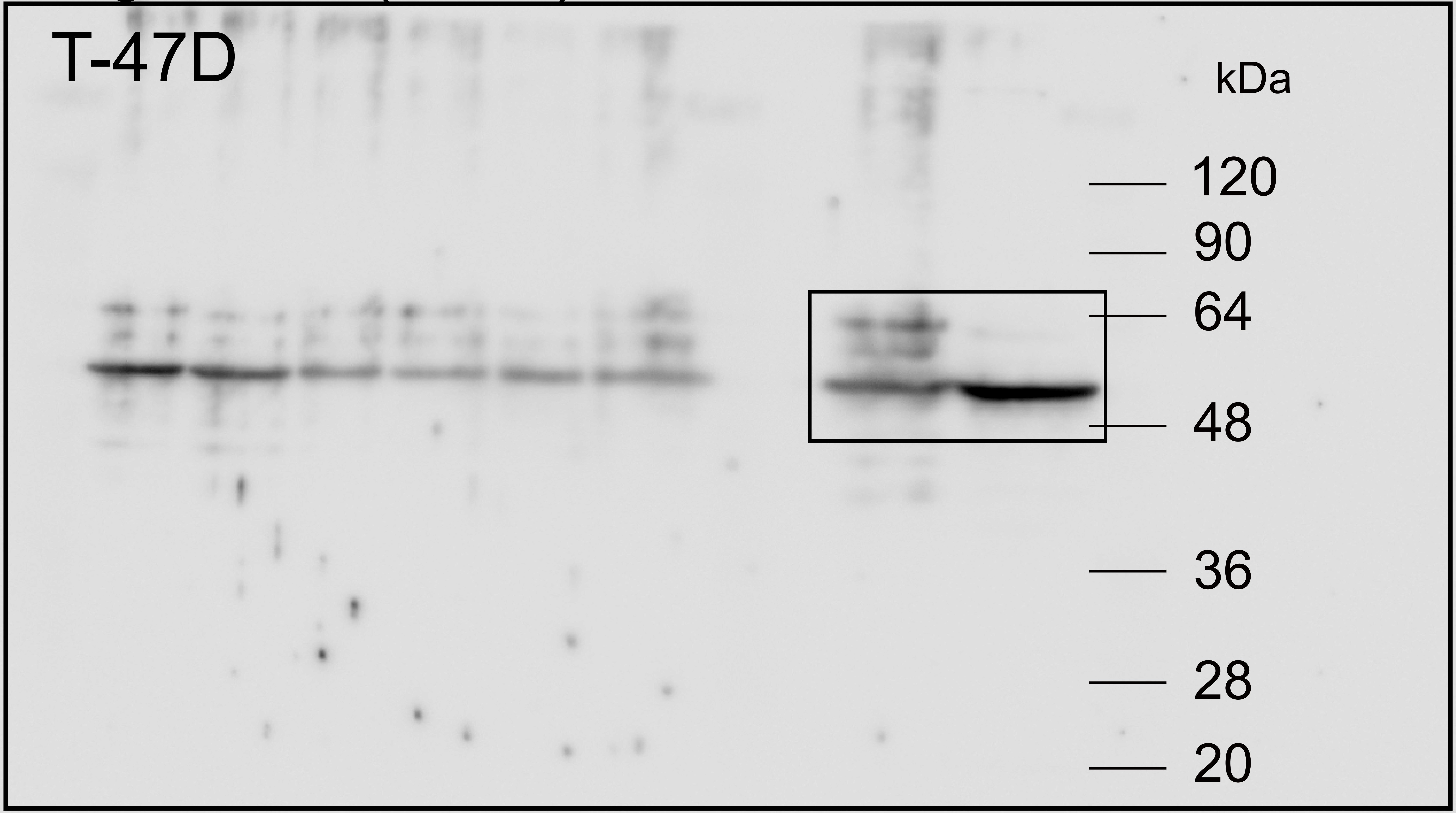

Figure 3B (CTH)
Left: MDA-MB-231
Right: T-47D

short exposure

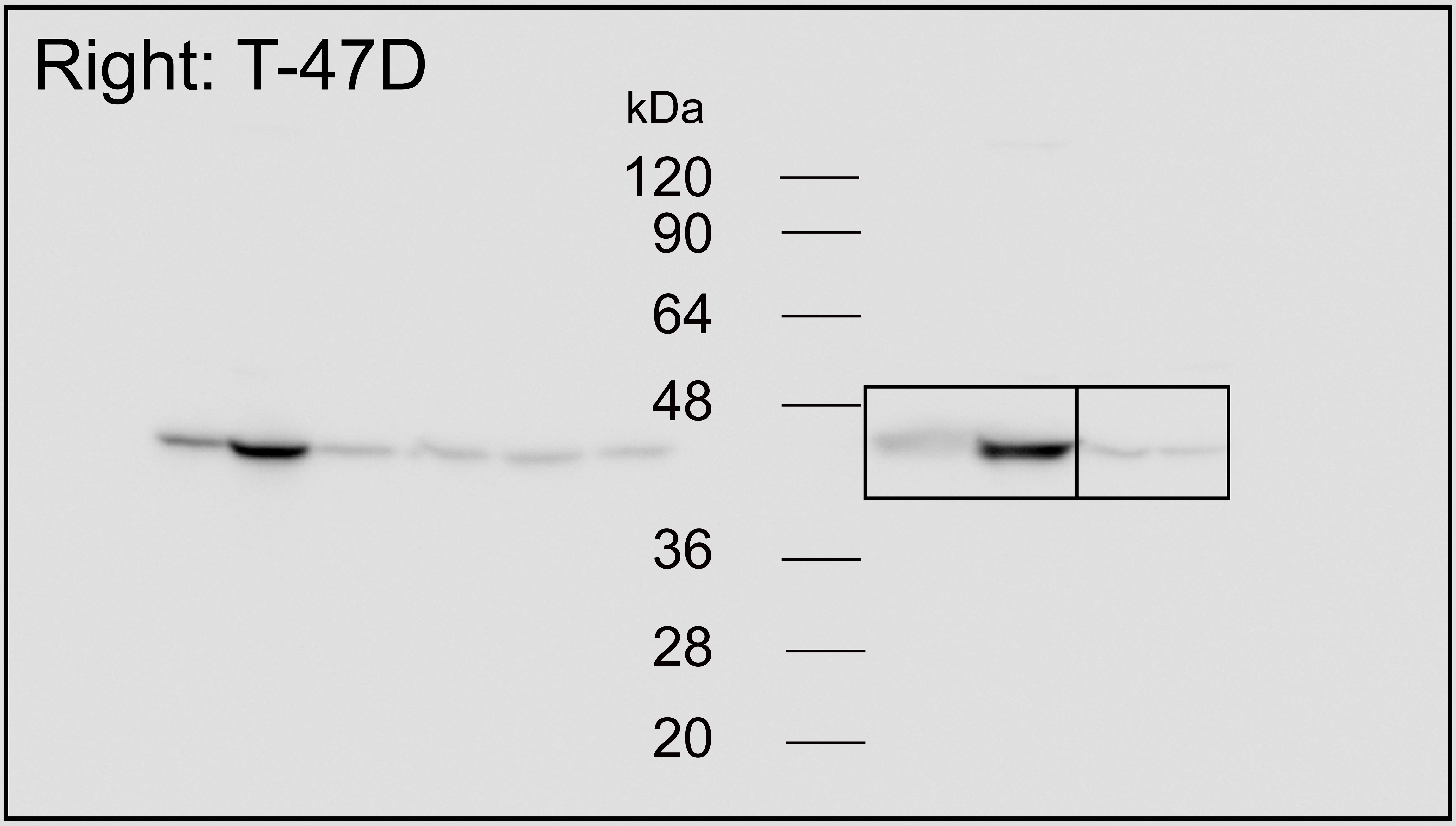

Figure 3B (CTH)
Left: MDA-MB-231
Right: T-47D

long exposure

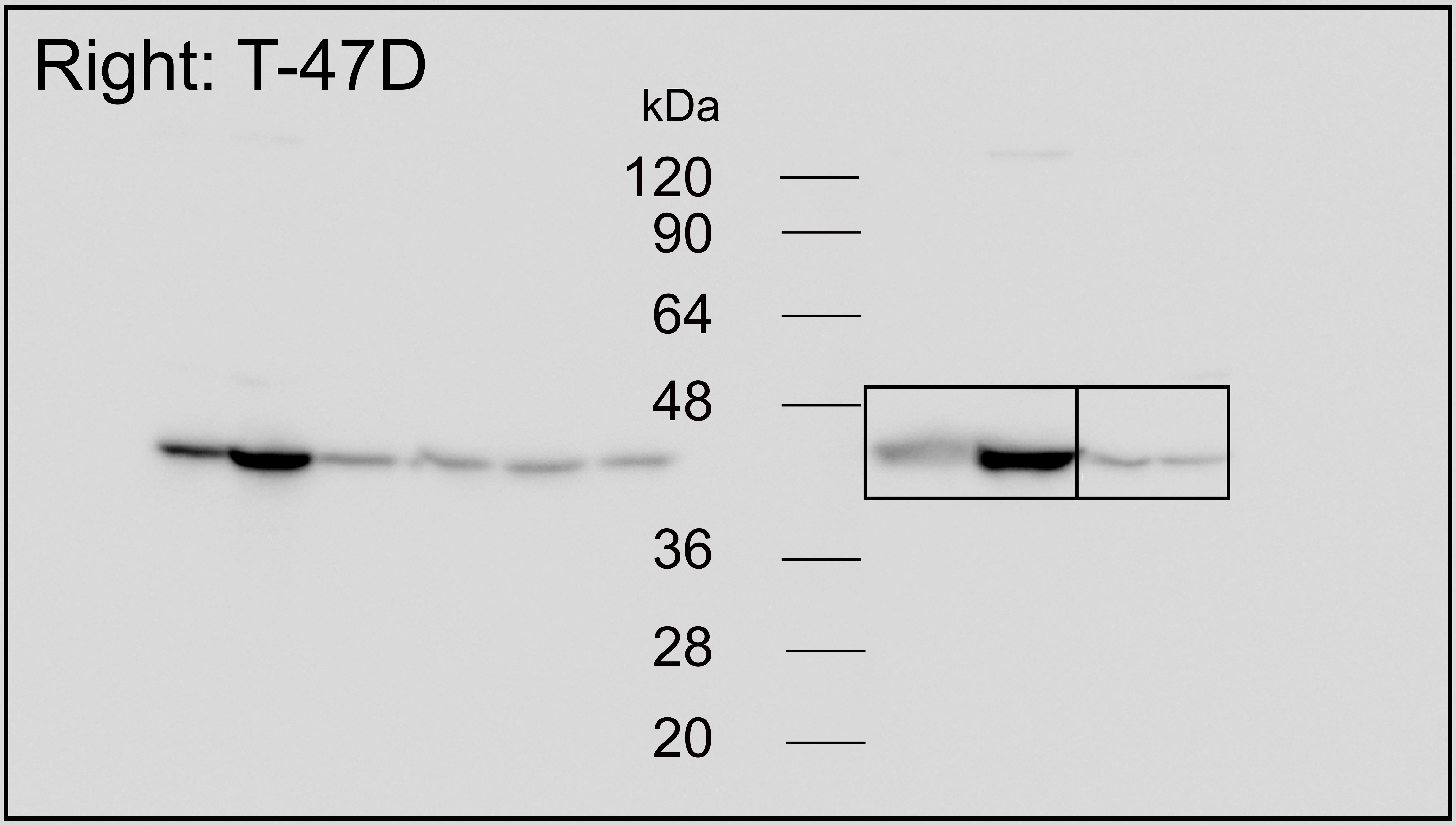

Figure 3B (p-GCN2)
Left: MDA-MB-231
Right: T-47D

short exposure

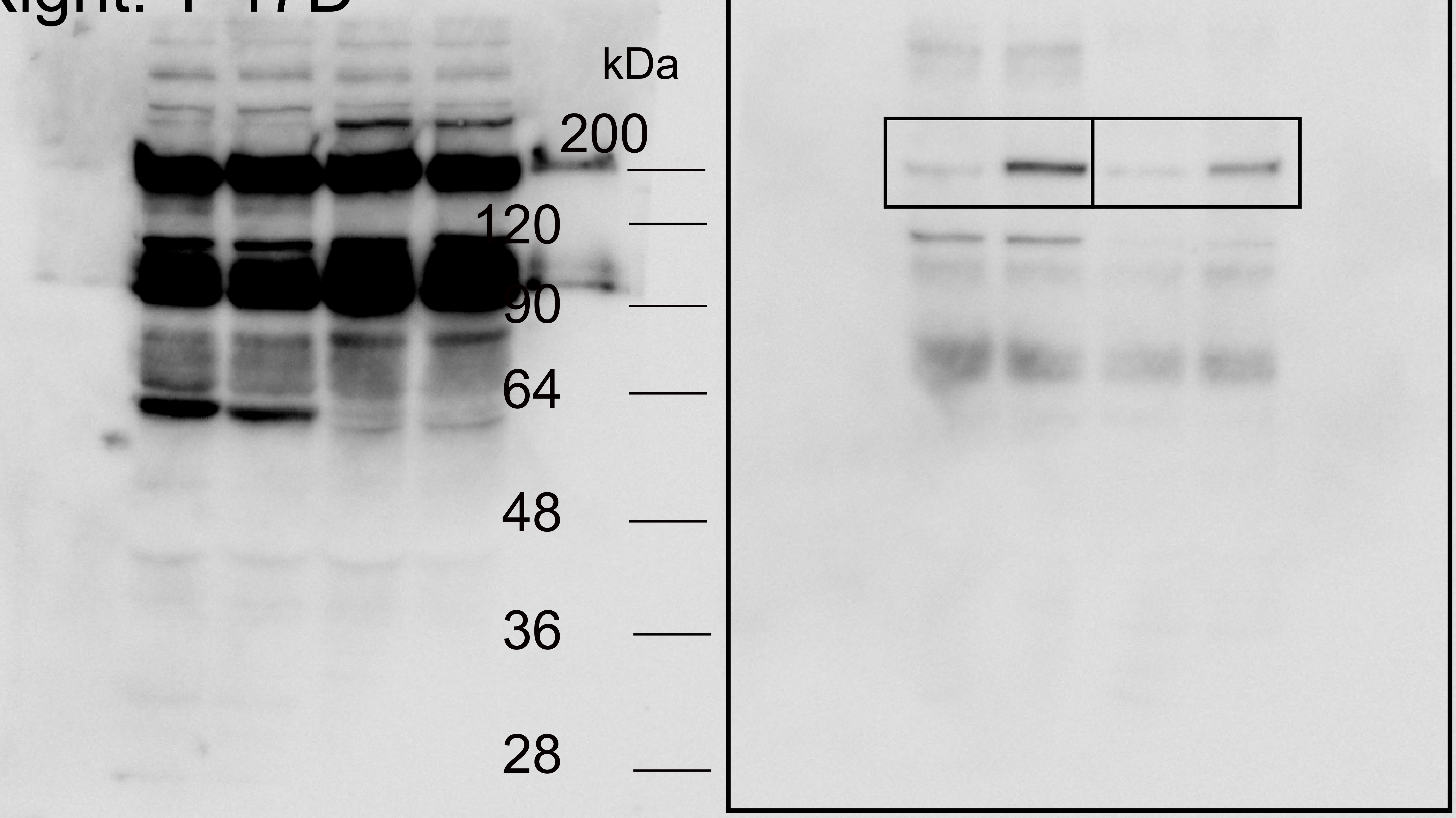

Figure 3B (p-GCN2)
Left: MDA-MB-231
Right: T-47D

long exposure

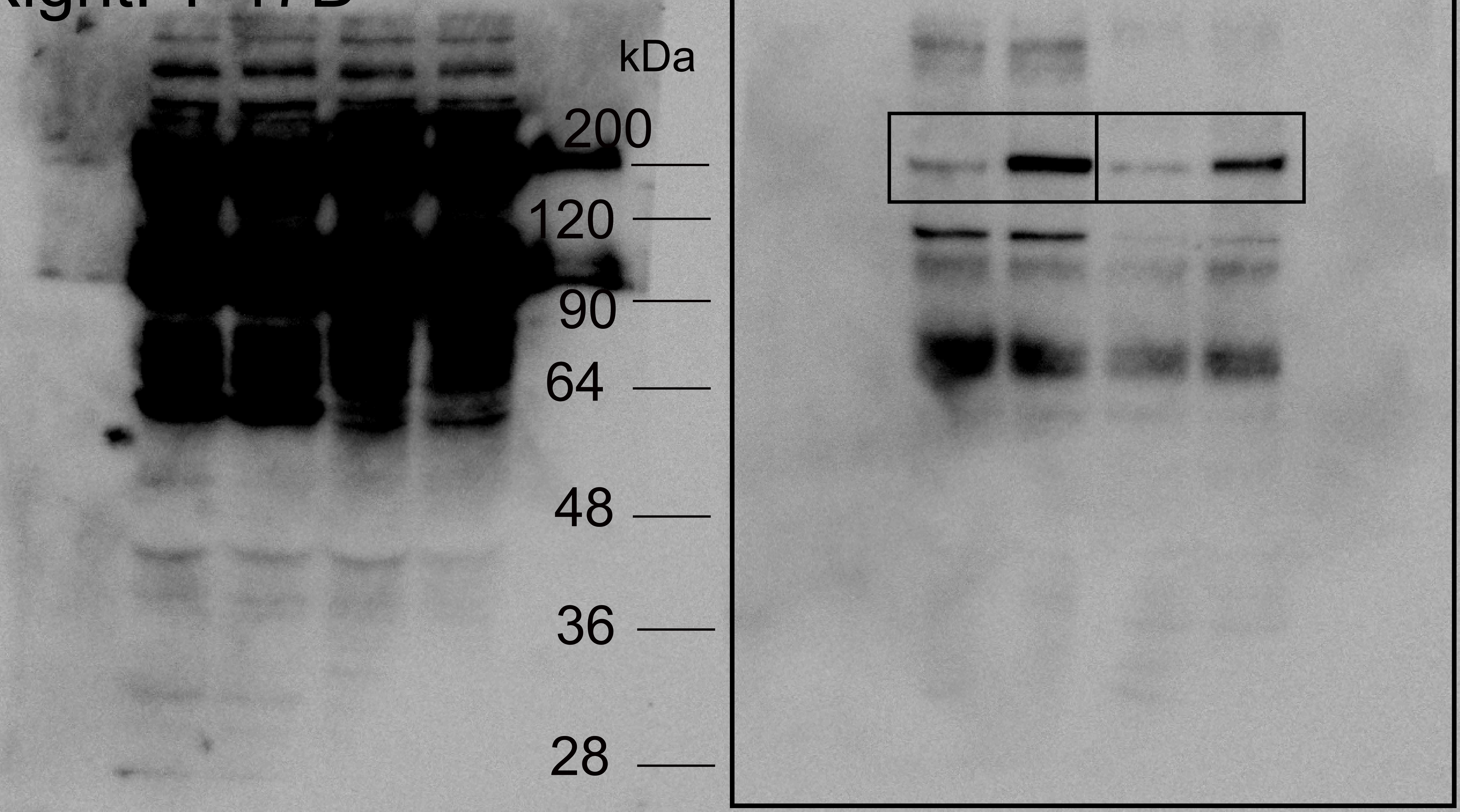

Figure 3B (GCN2)
Left: MDA-MB-231
Right: T-47D

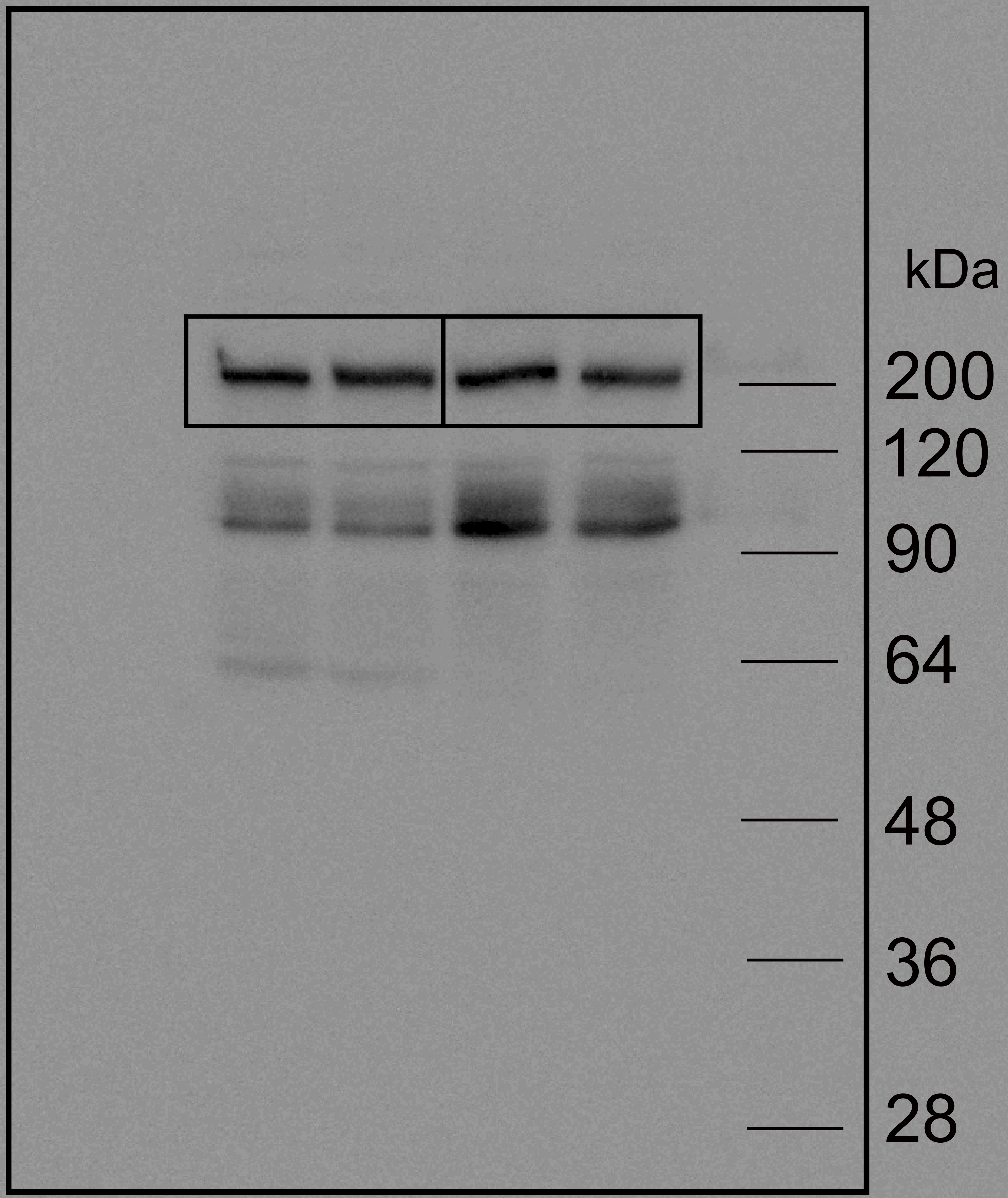

Figure 3B (p-EIF2α)

Left: MDA-MB-231

Right: T-47D

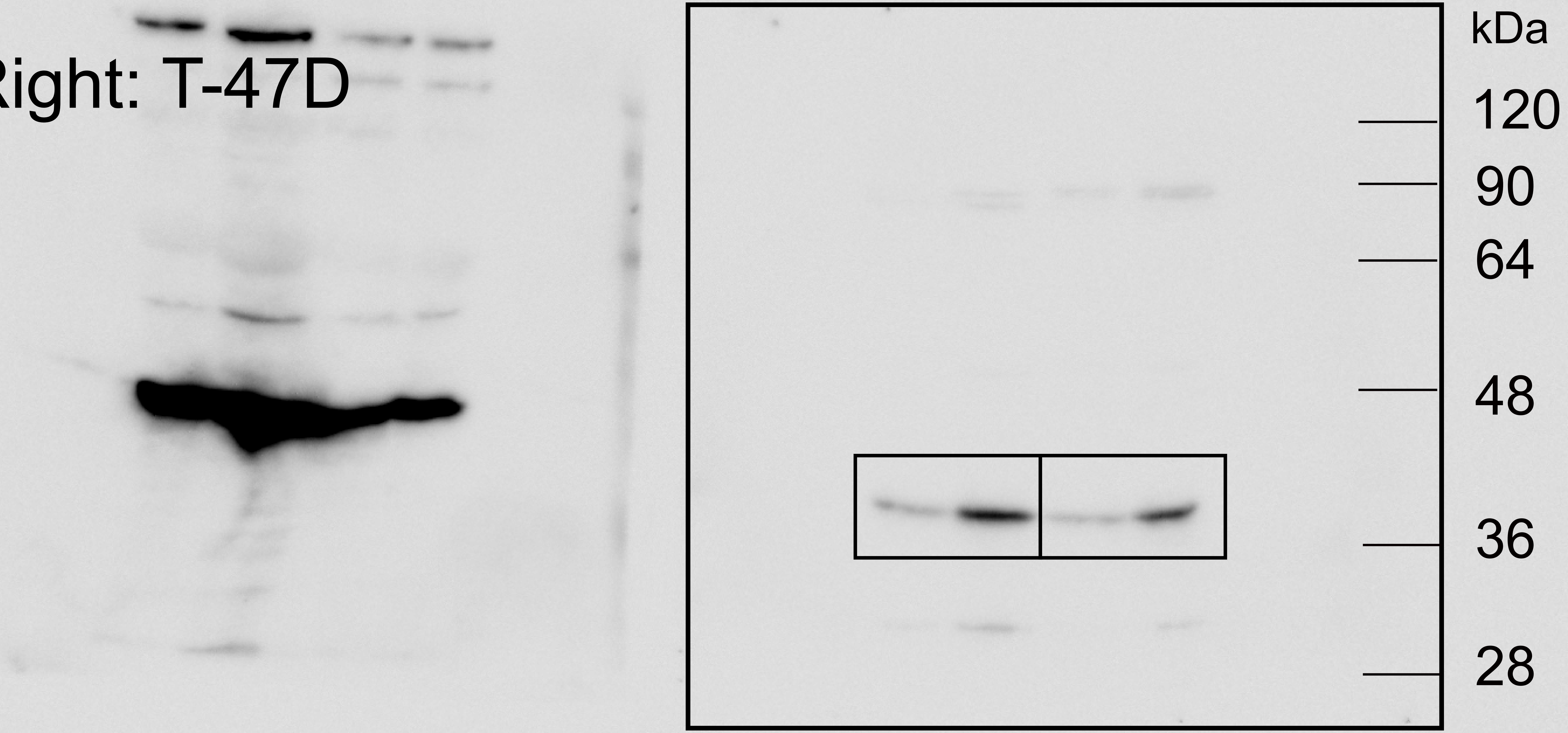

Figure 3B (EIF2α)

Left: MDA-MB-231

Right: T-47D

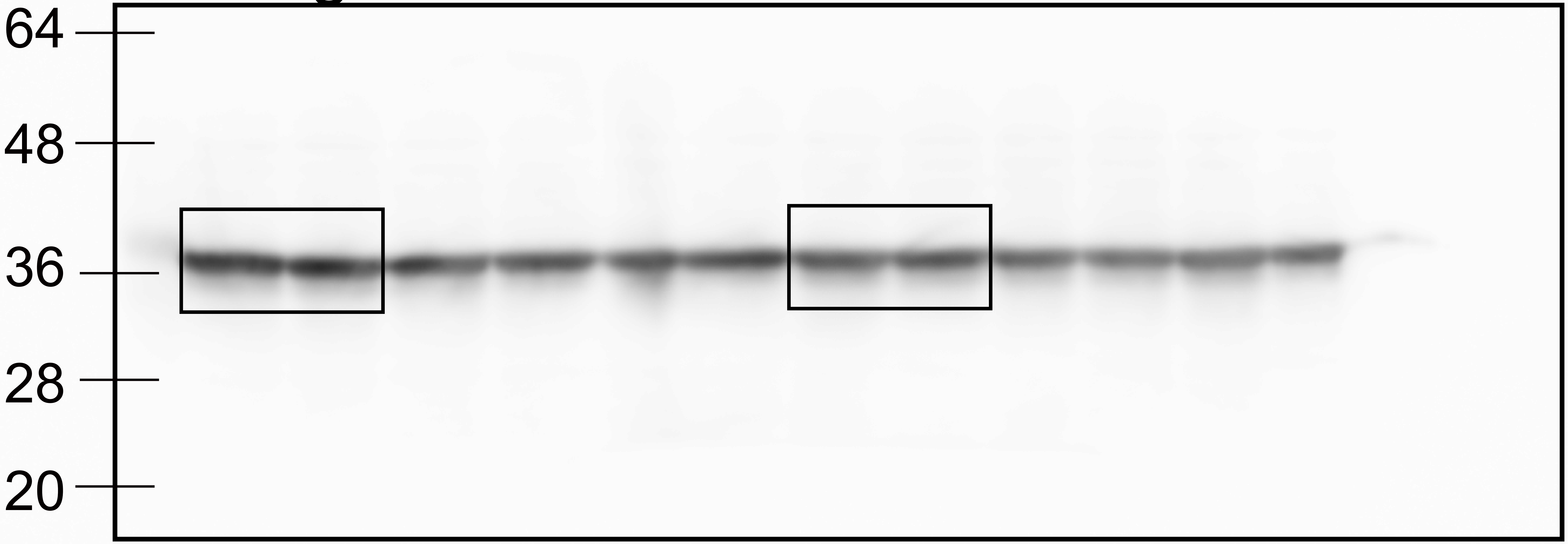

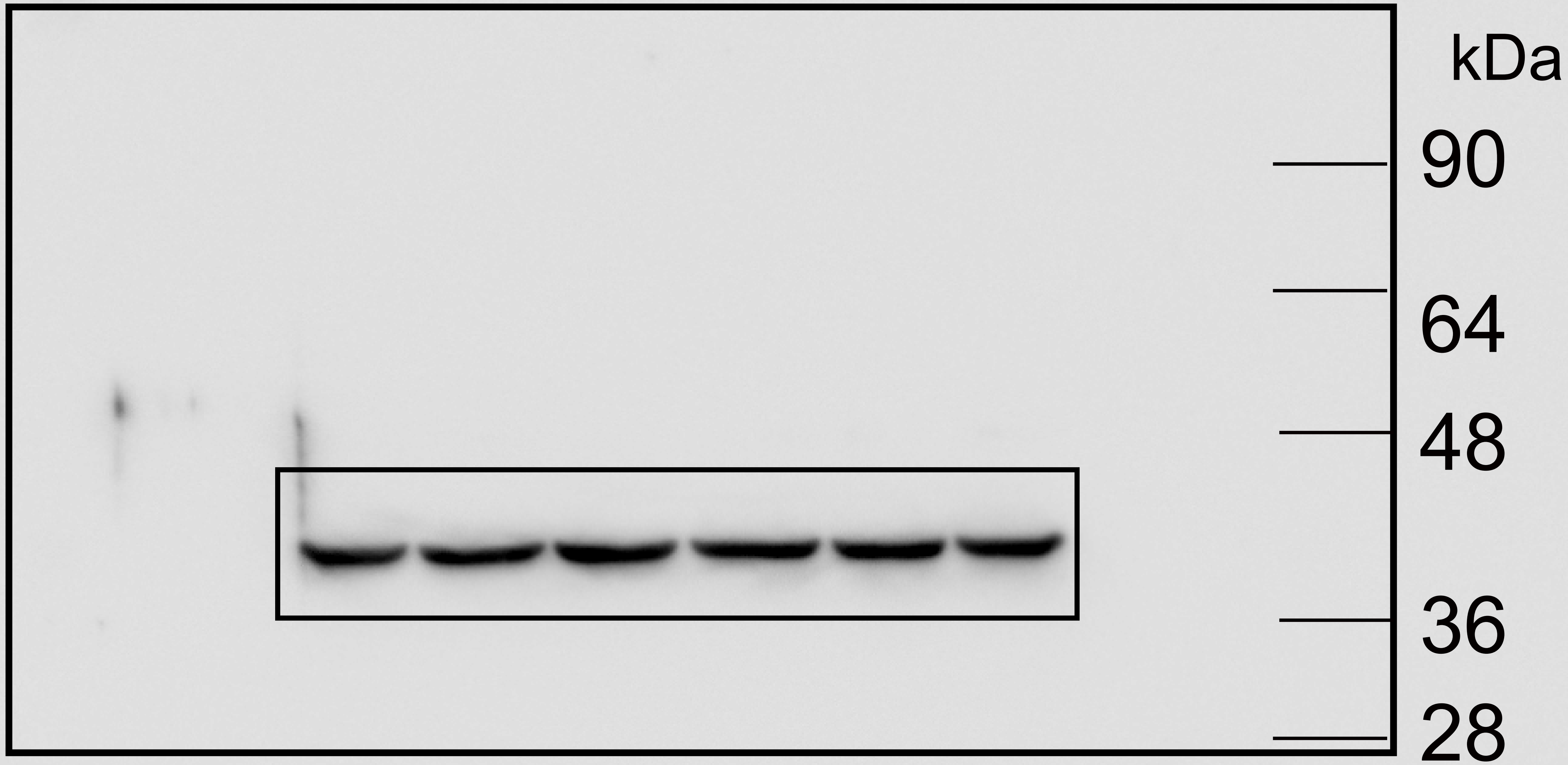

Figure 3C (β-actin)

Figure 3C (CTH)

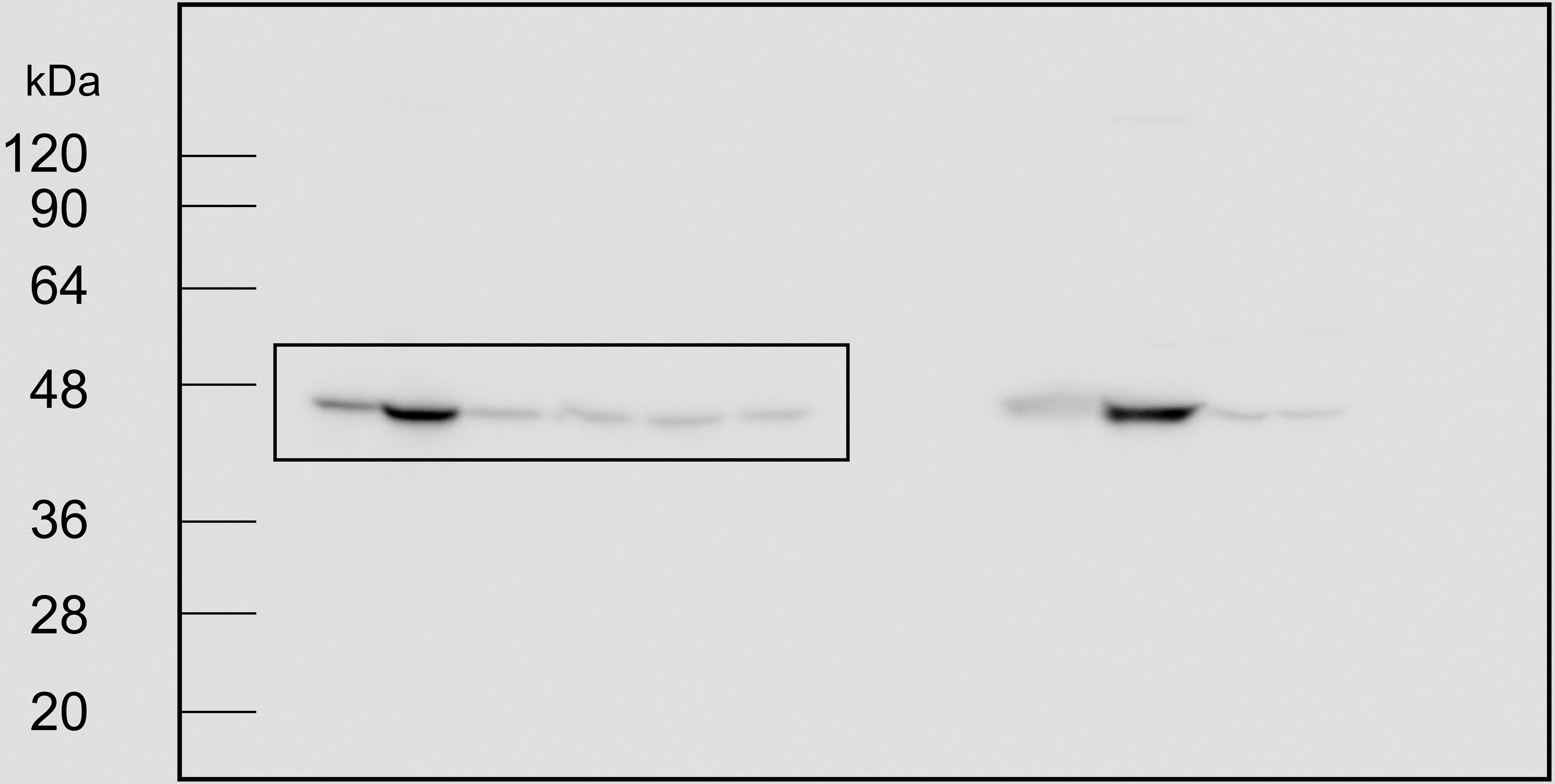

Figure 3C (ATF4)

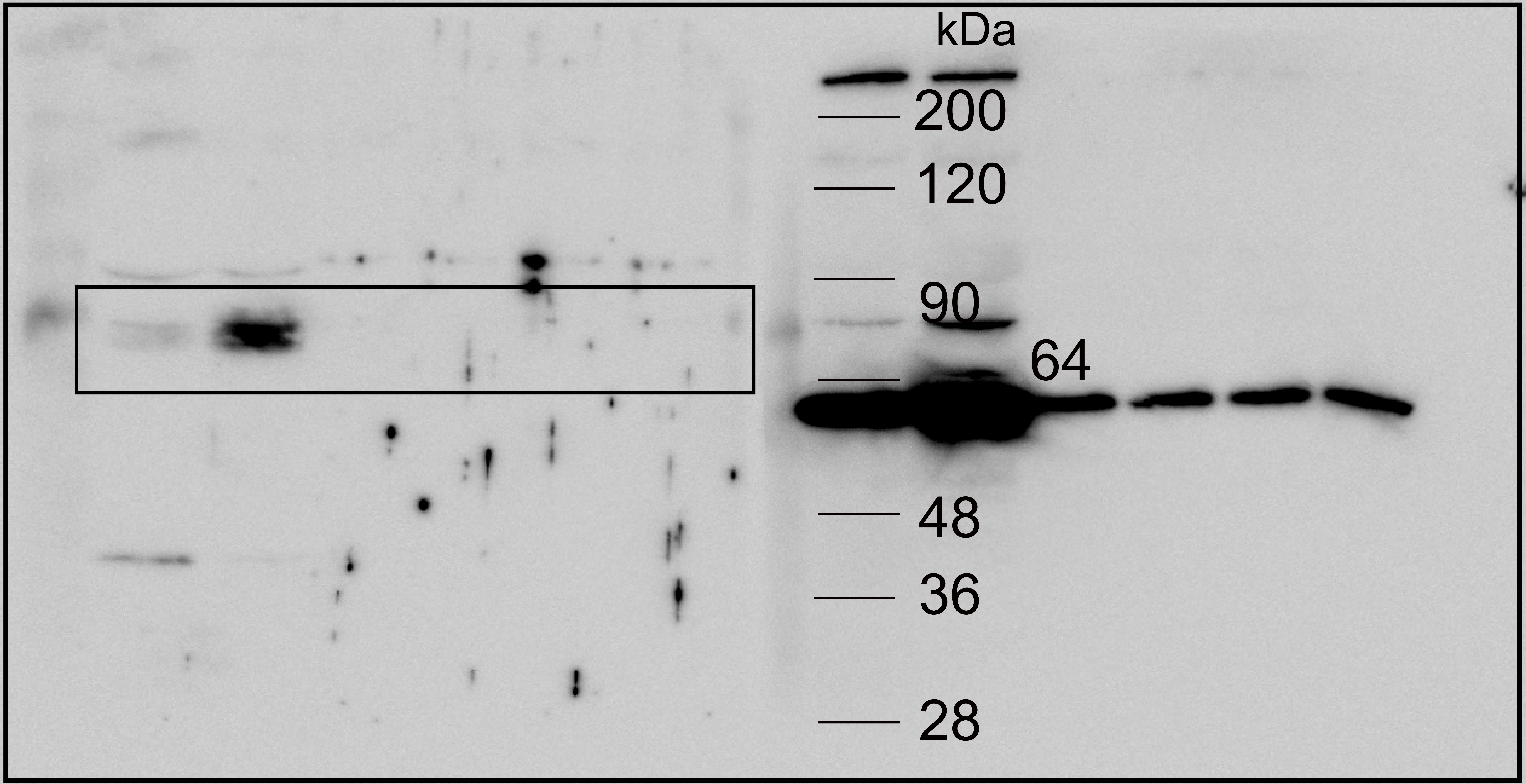

Figure 3D (β-actin)

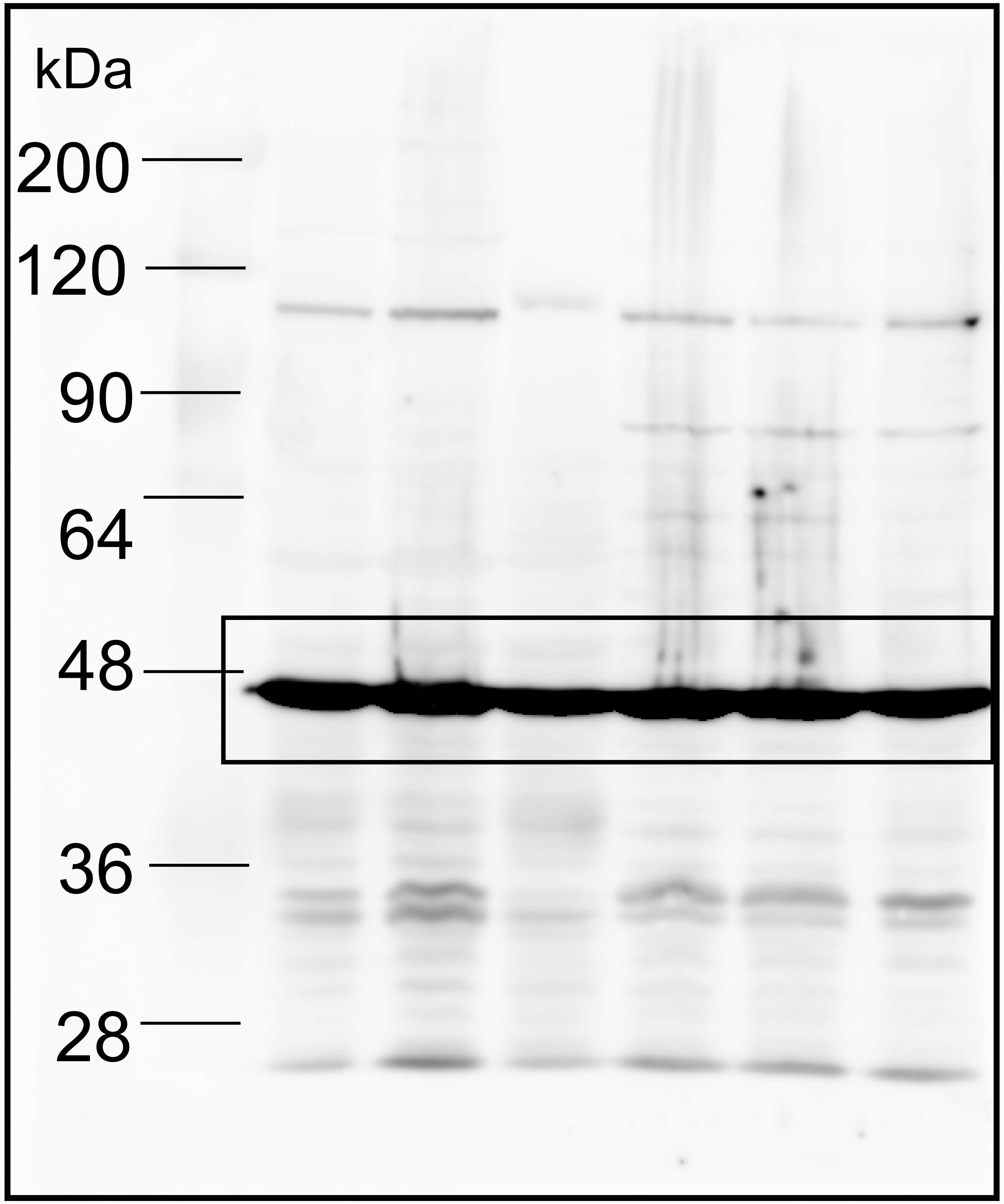

Figure 3D (CTH)

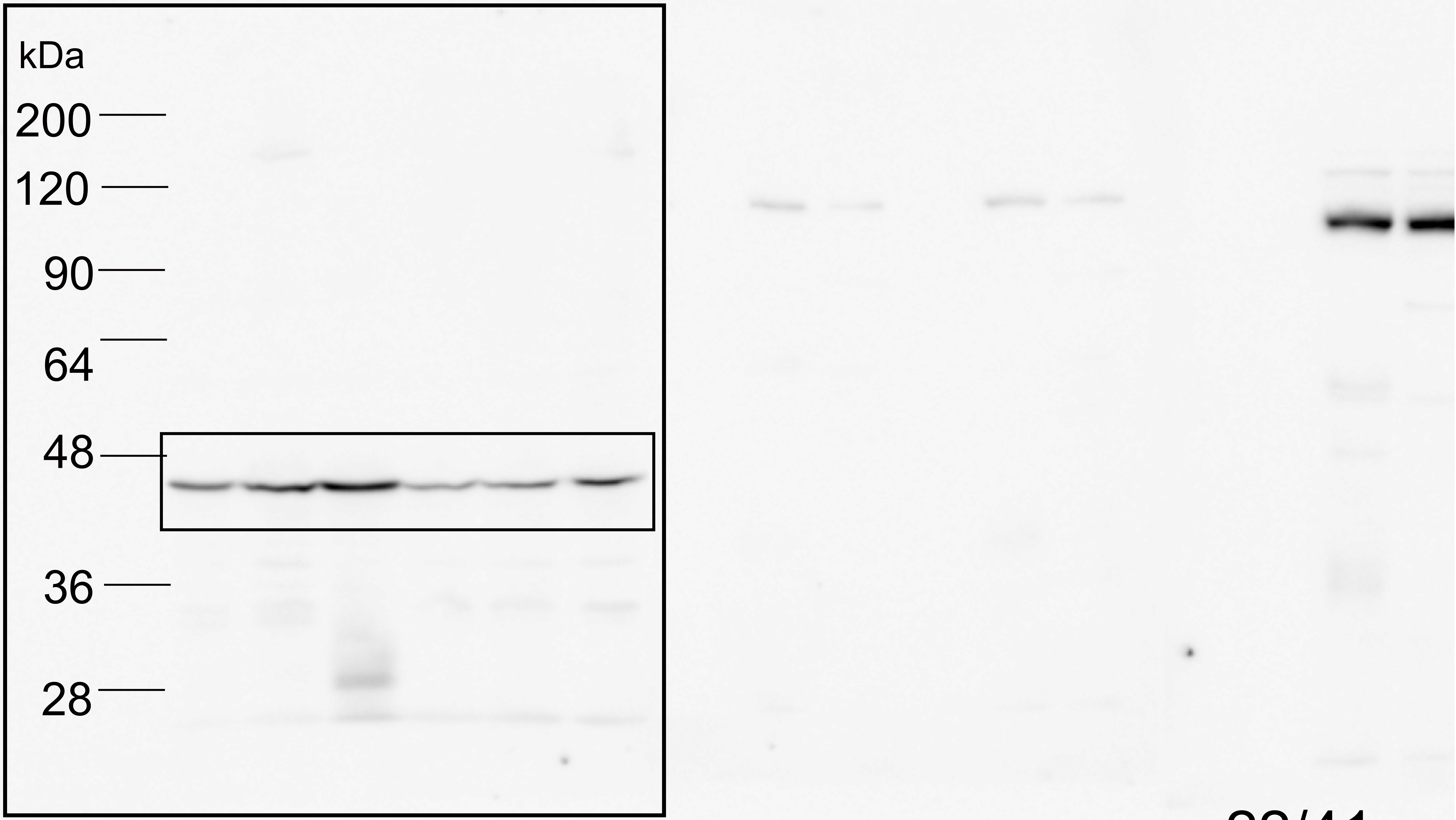

Figure 3E (CTH)

short exposure

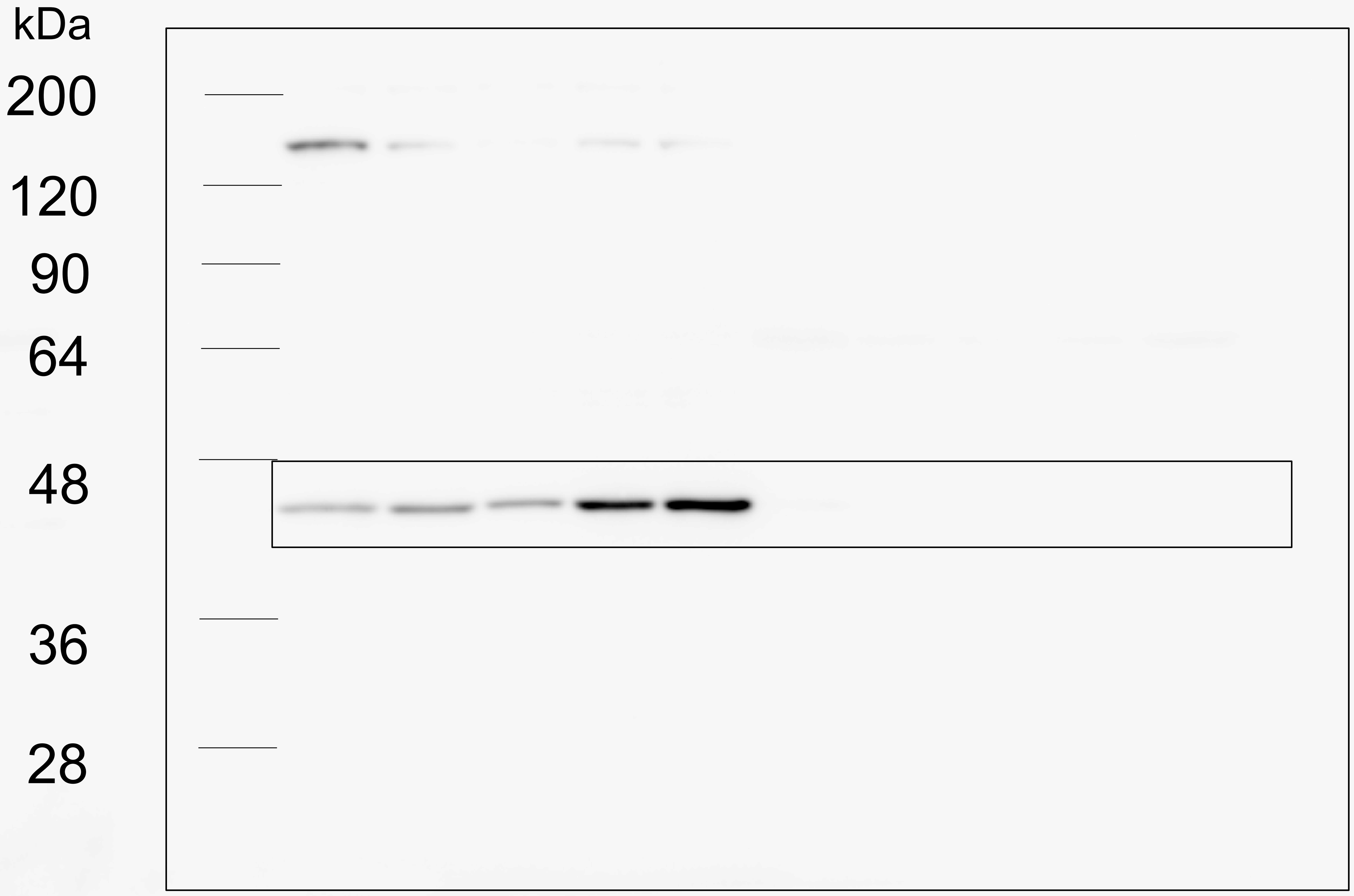

long exposure

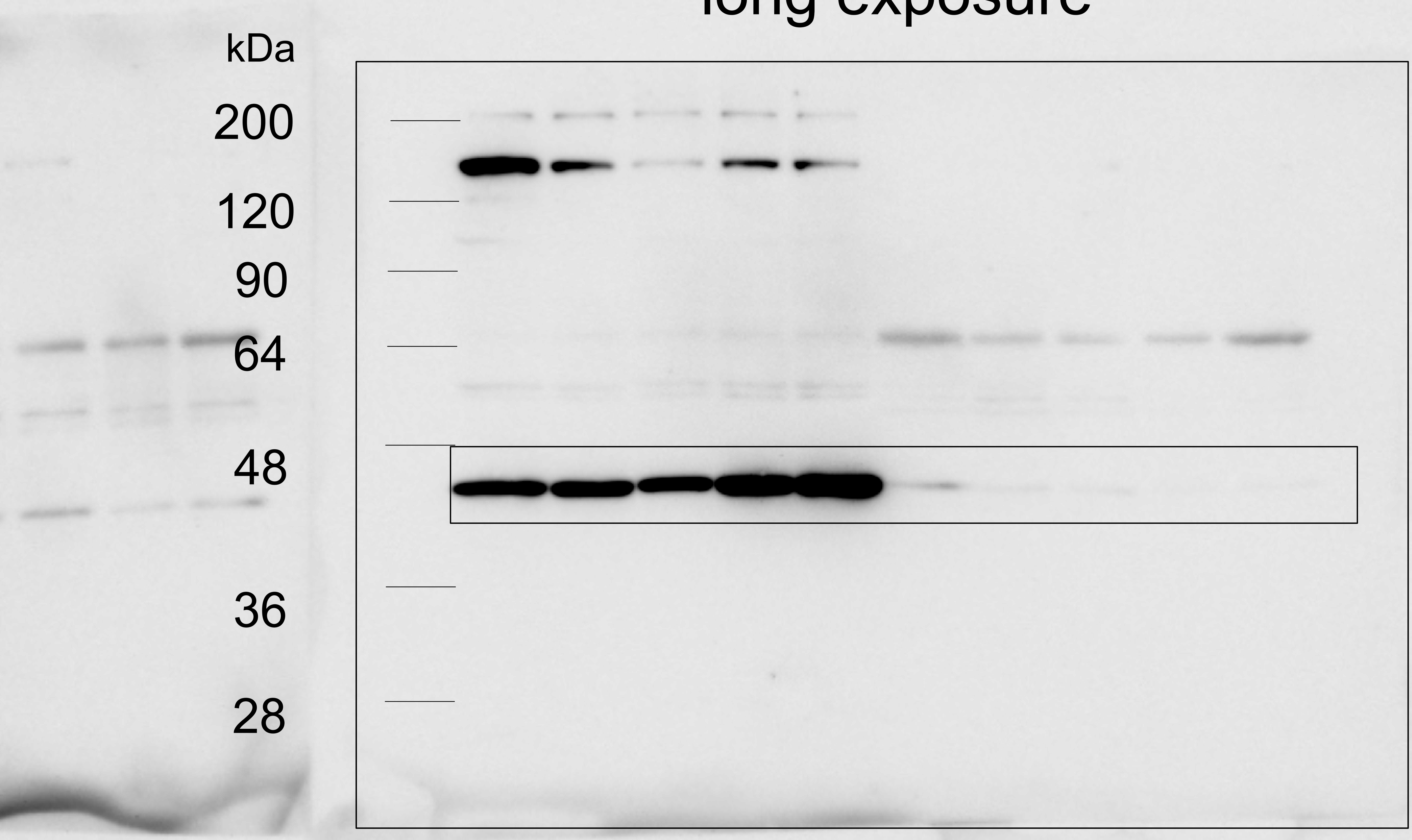

Figure 3E (β-actin)

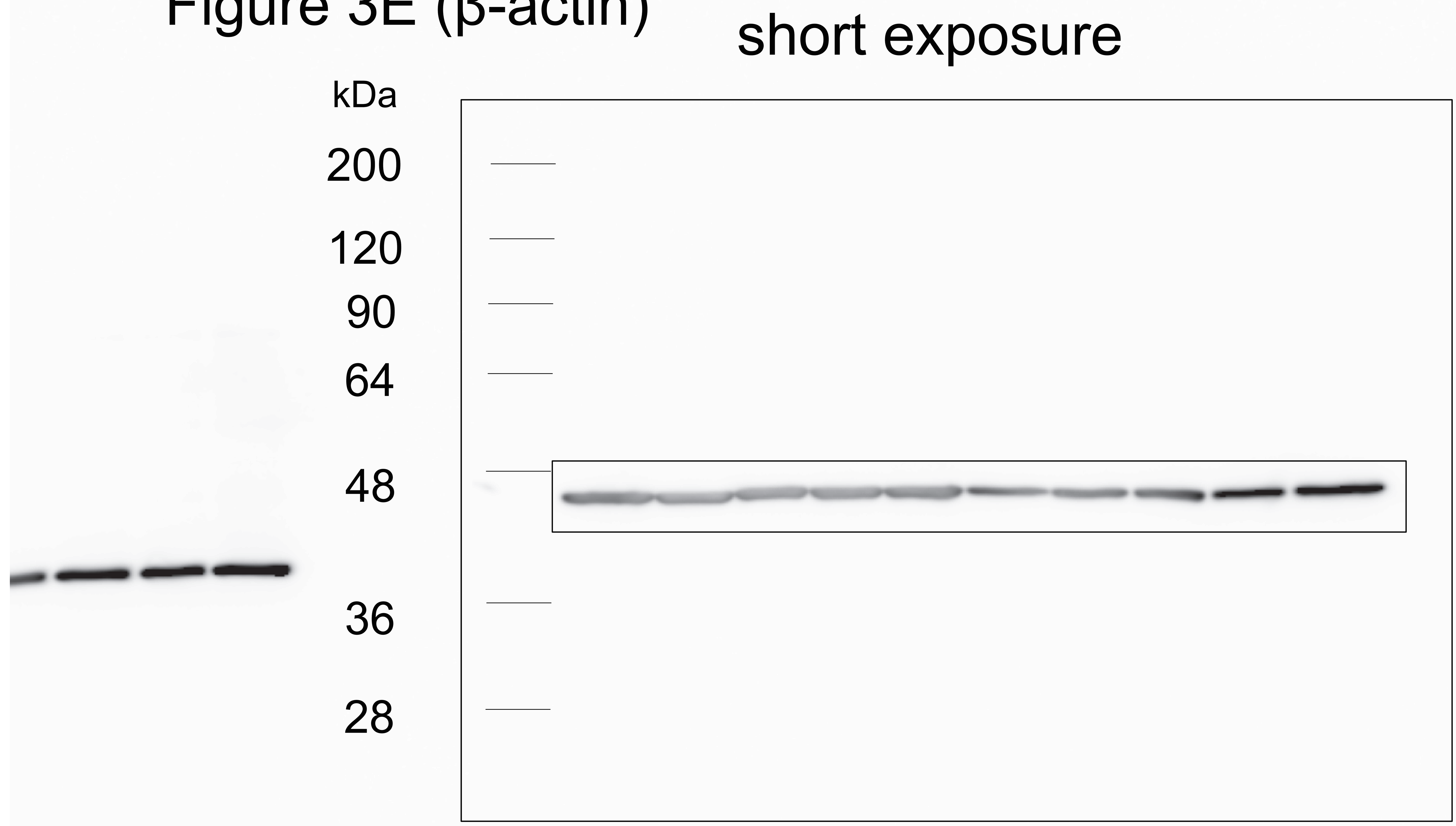

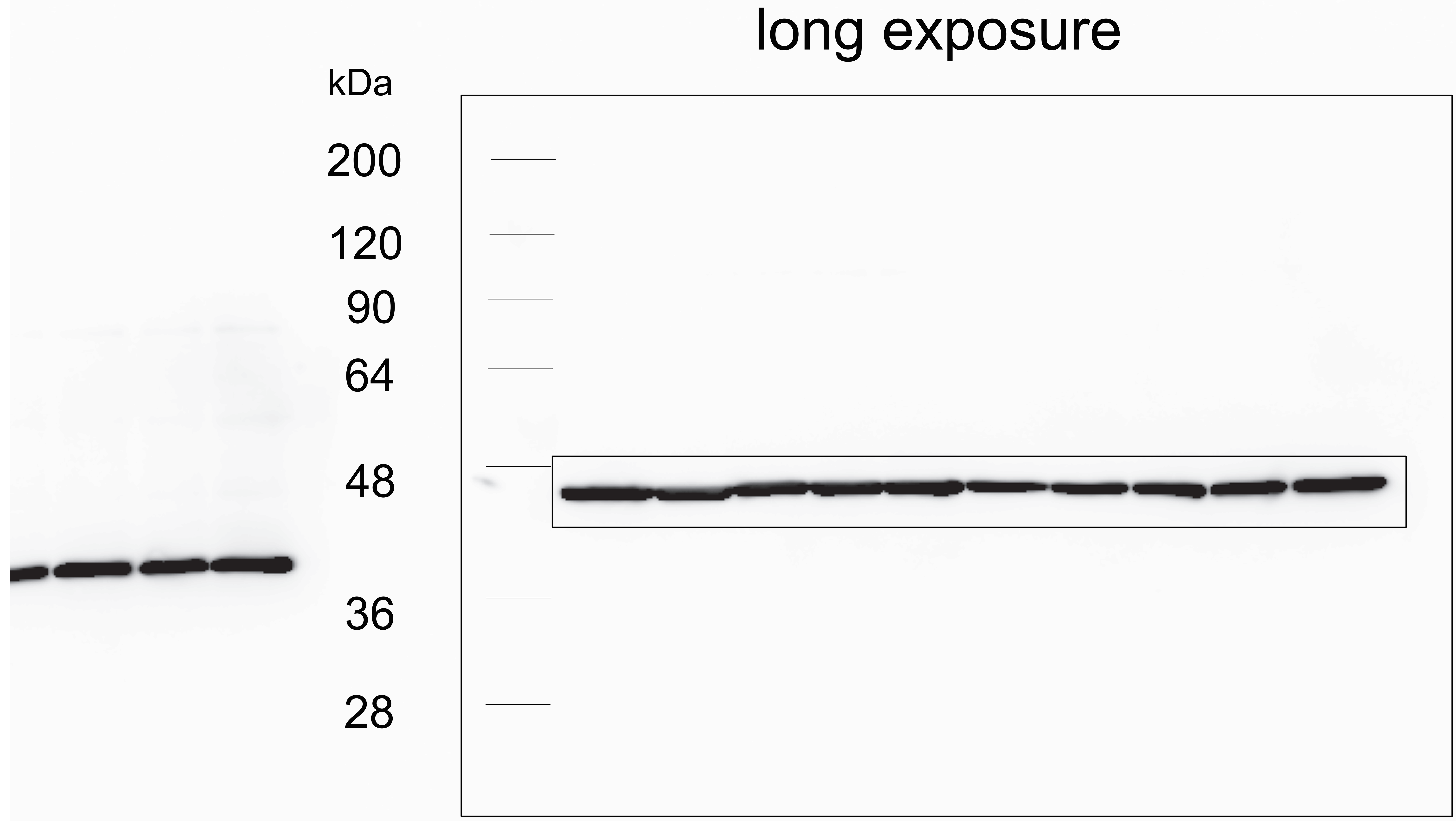

Figure 4A (β-actin)
MDA-MB-231

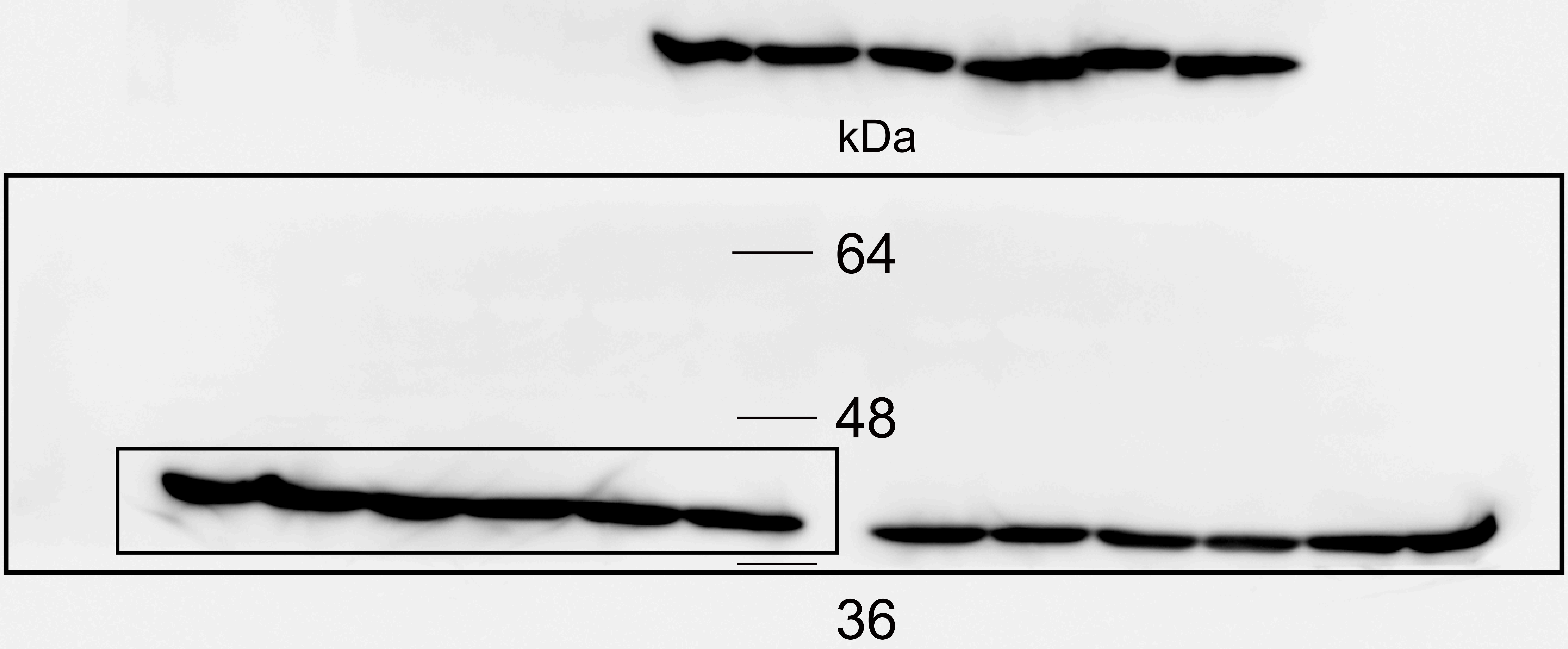

Figure 4A (β-actin)

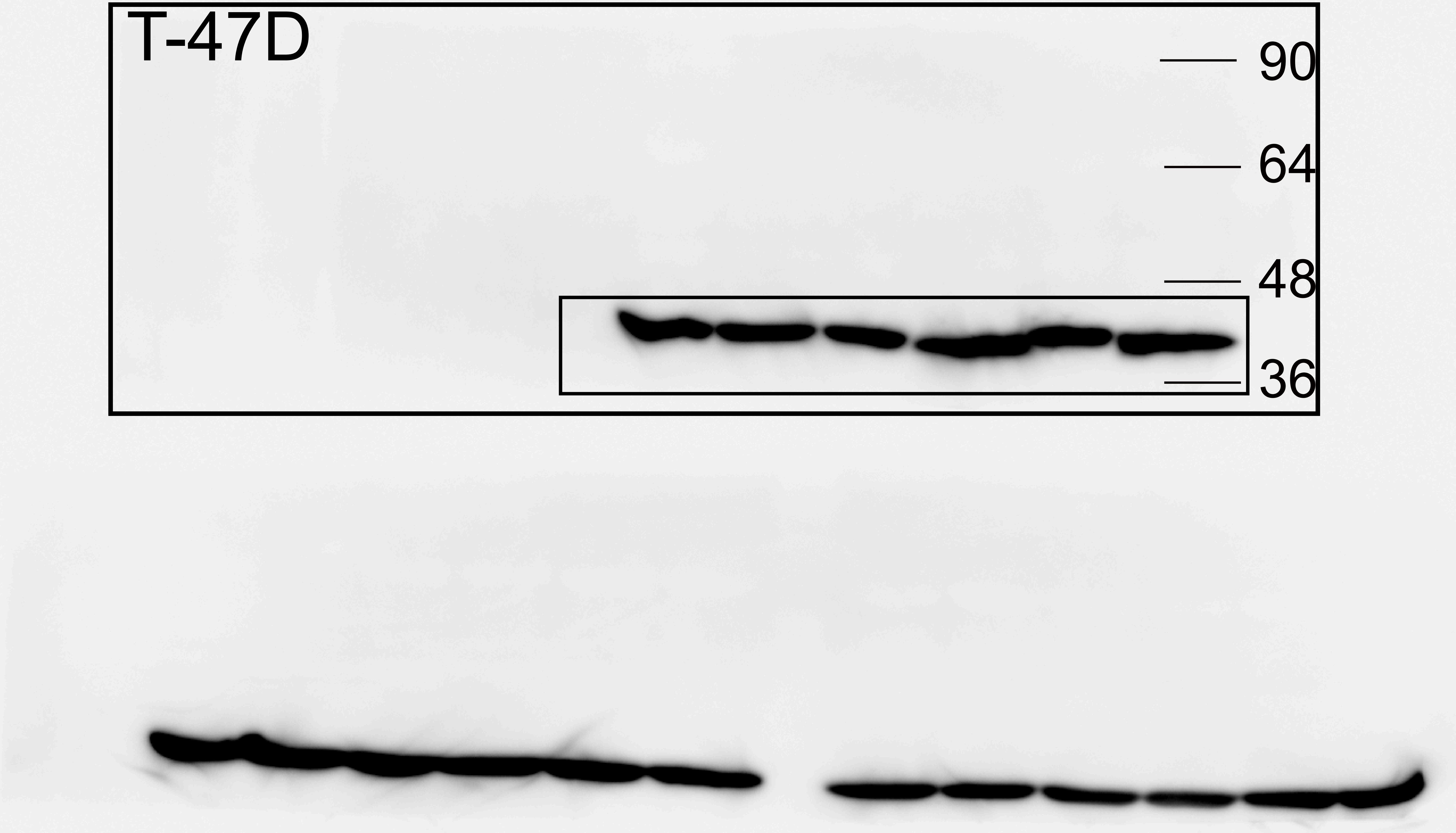

Figure 4A (GCN2)
MDA-MB-231

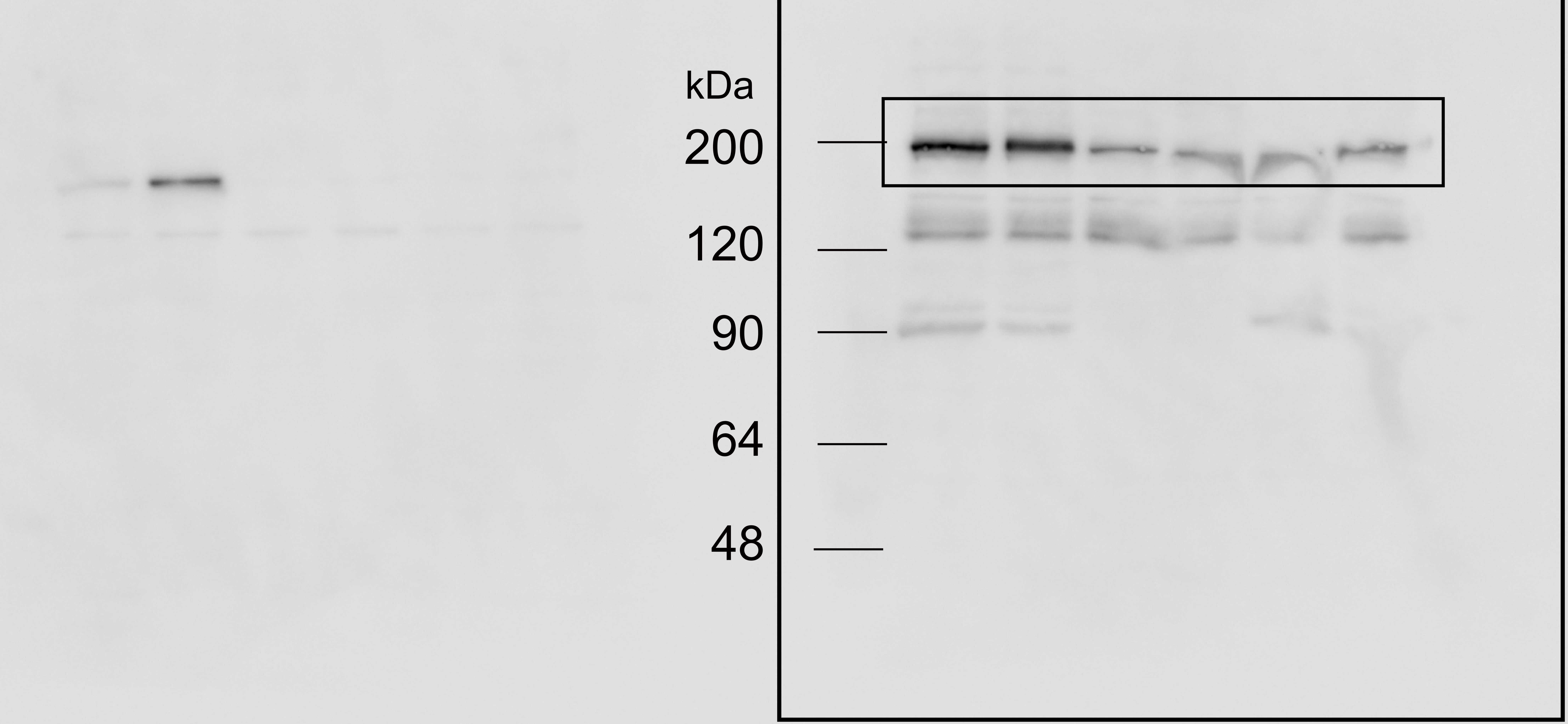

Figure 4A (GCN2)
T-47D

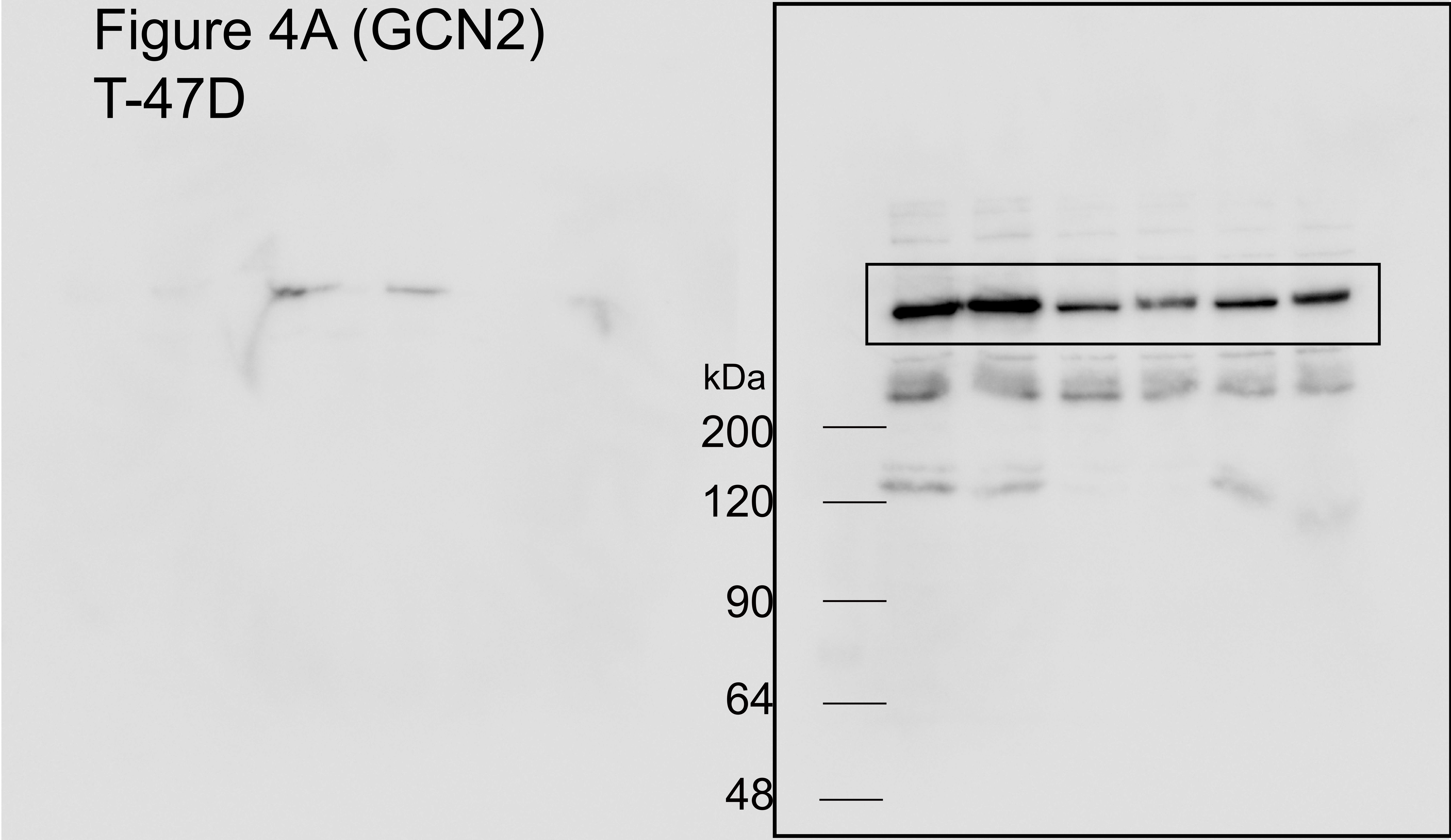

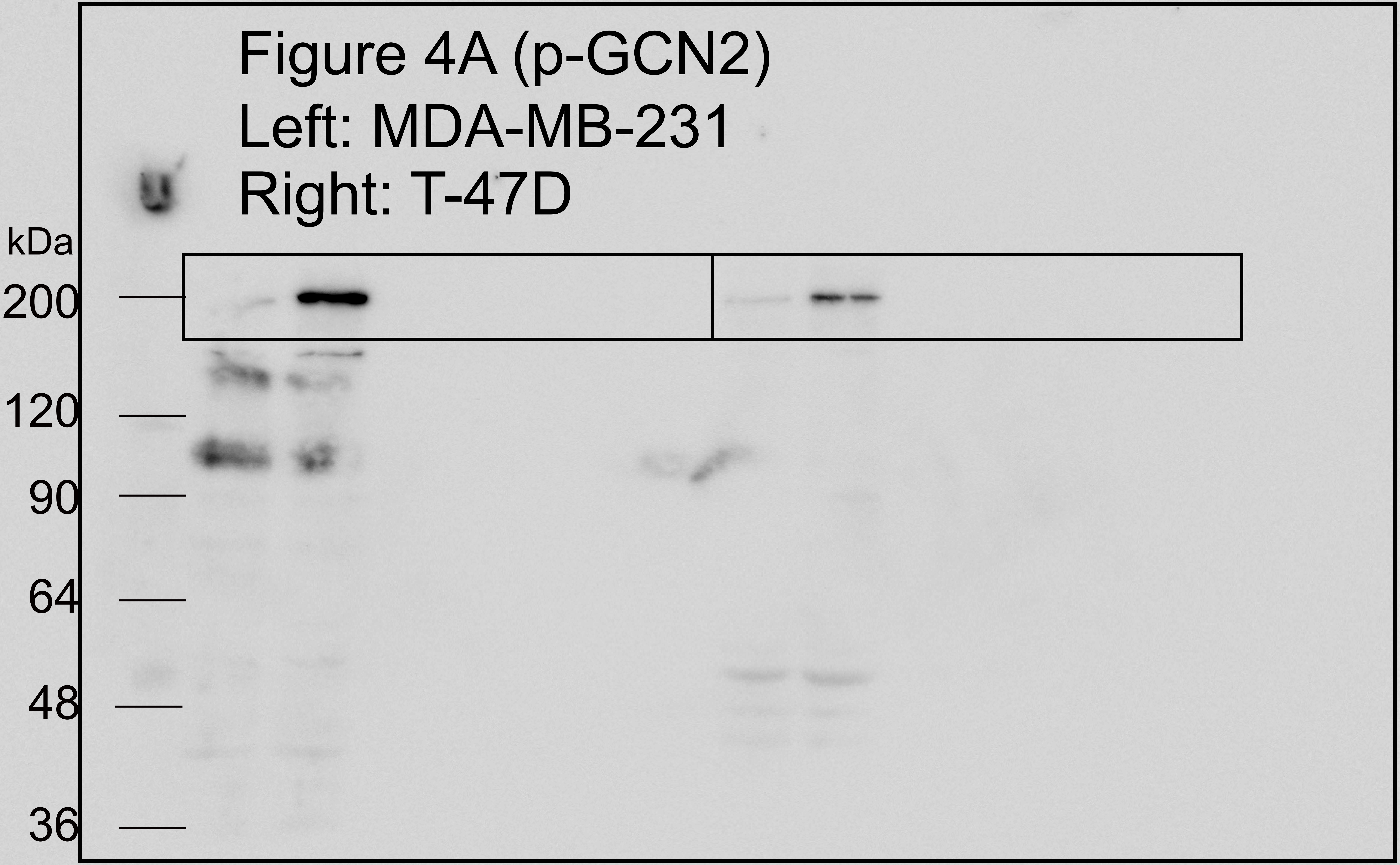
Figure 4A (p-GCN2)
Left: MDA-MB-231
Right: T-47D
kDa
200
120
90
64
48
36

Figure 4C (β-actin)
Left: MDA-MB-231
Right: T-47D

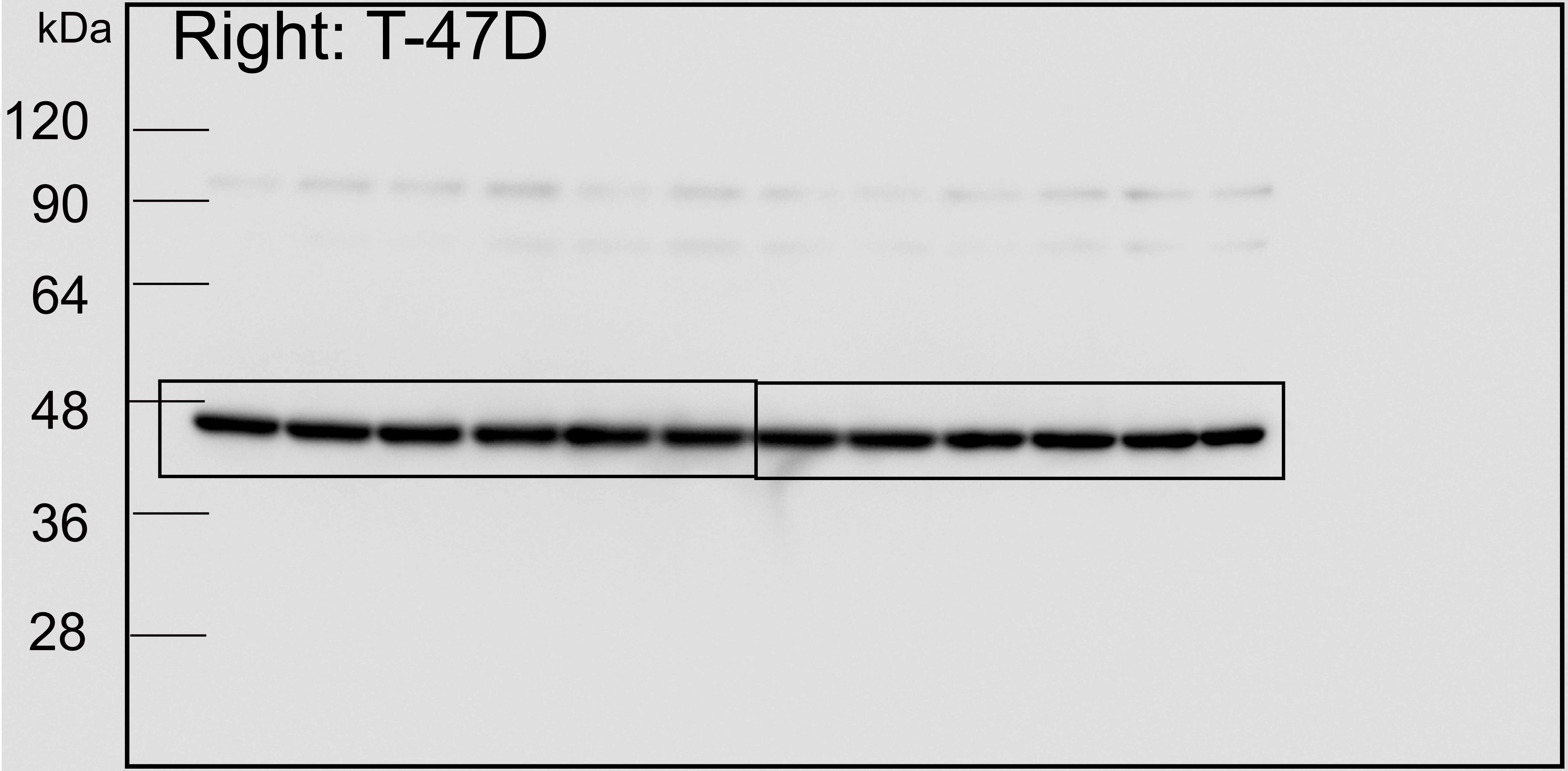

Figure 4C (ATF4)
MDA-MB-231

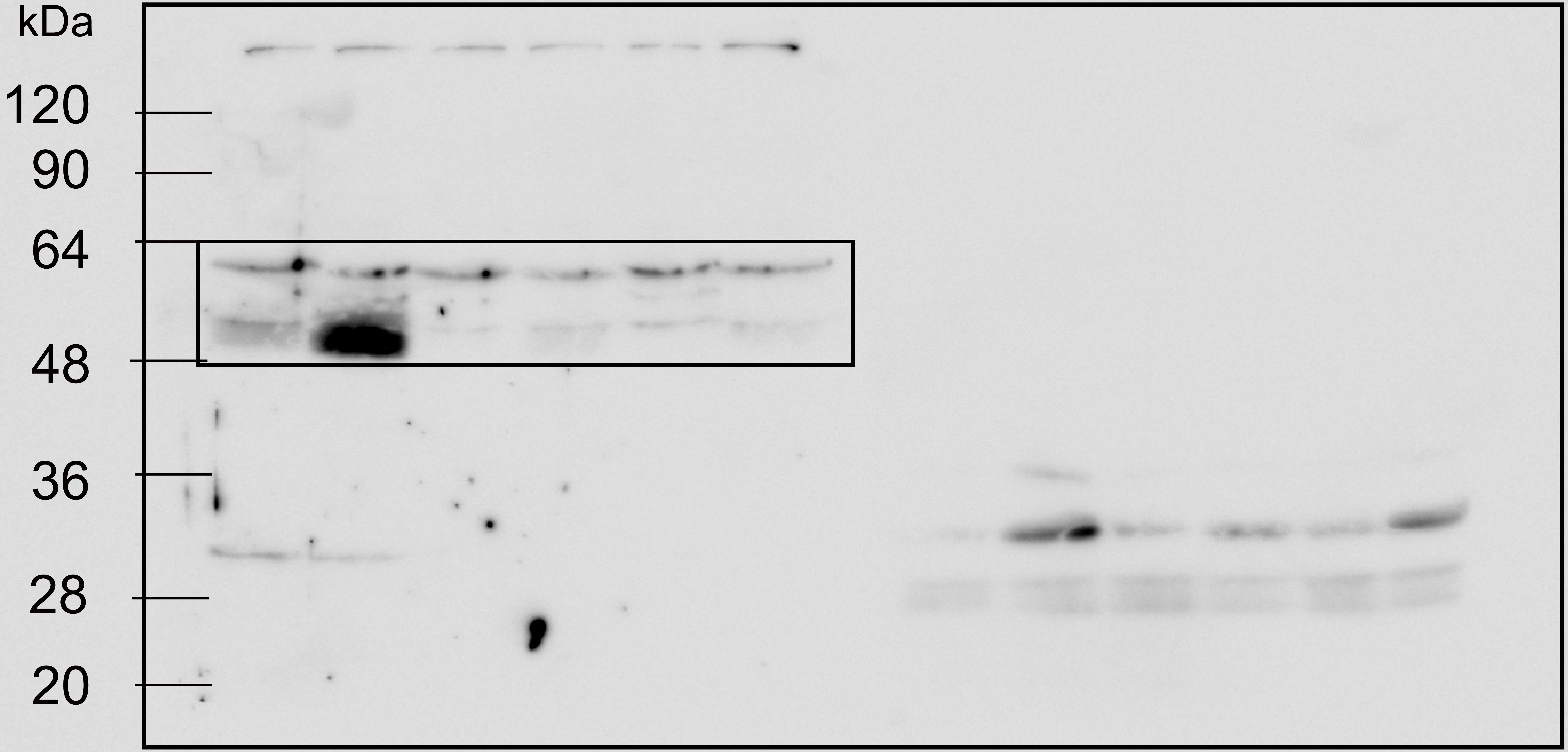

Figure 4C (ATF4)
T-47D

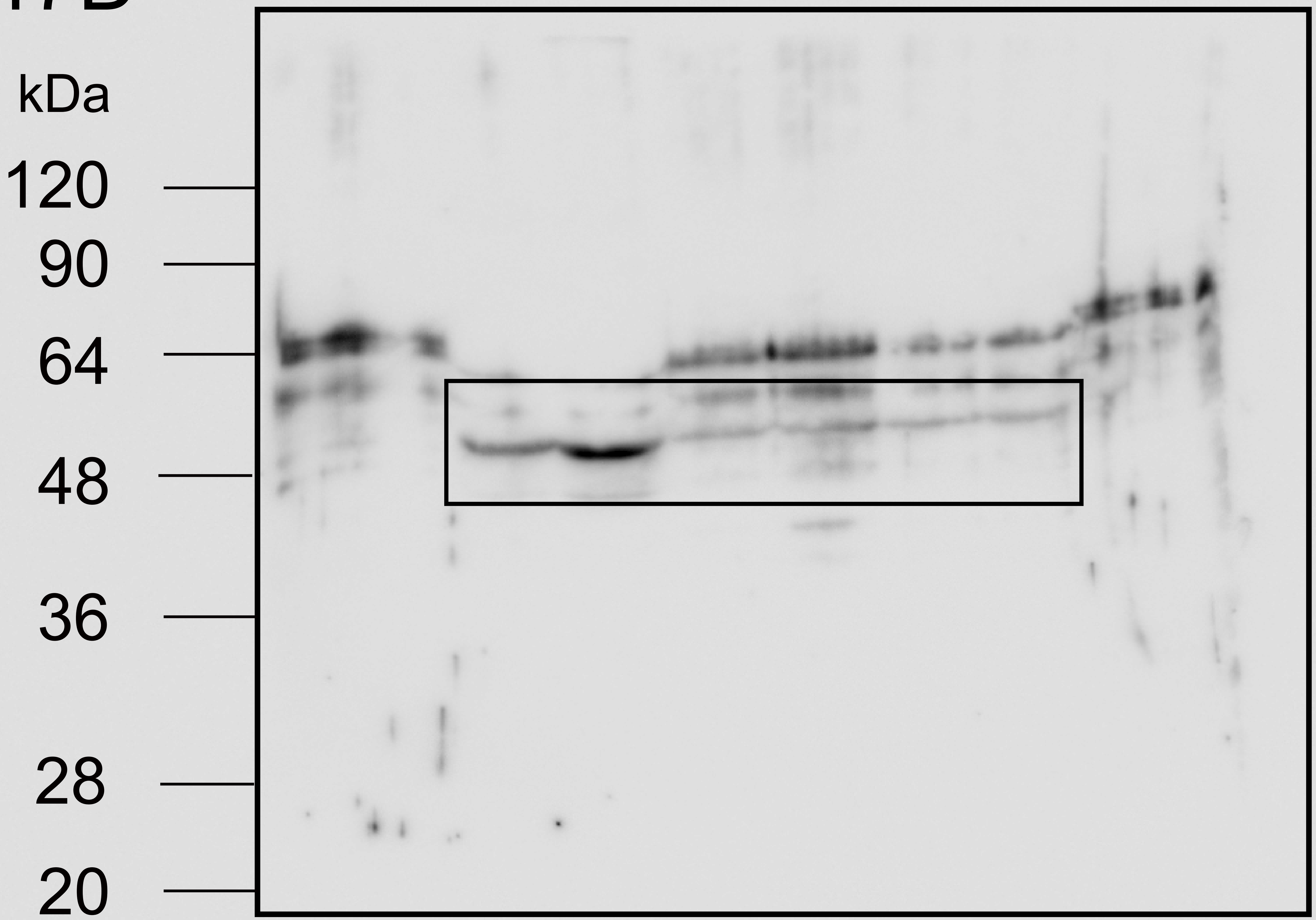

Figure 4E (β-actin)
MDA-MB-231

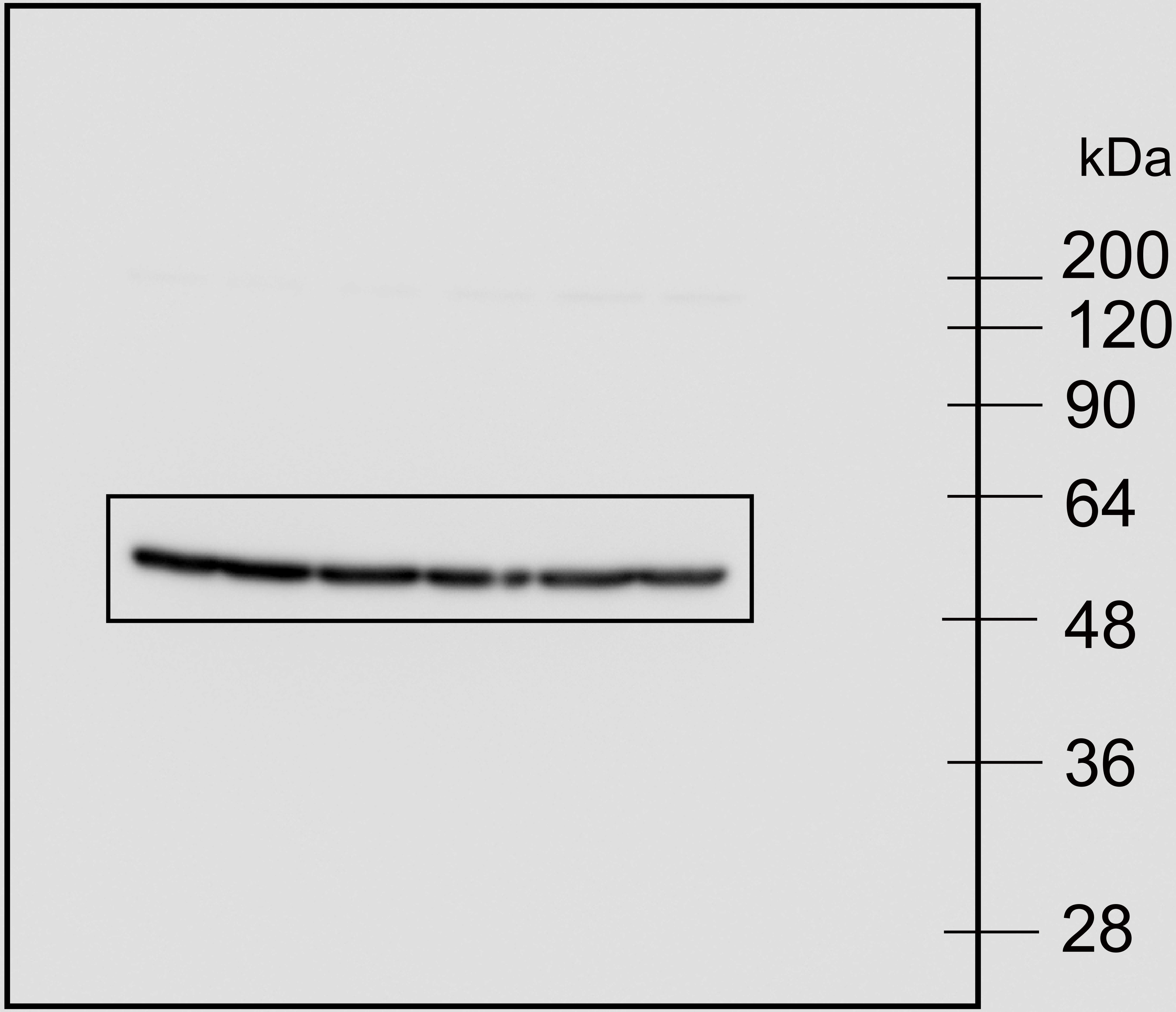

Figure 4E (β-actin)
T-47D

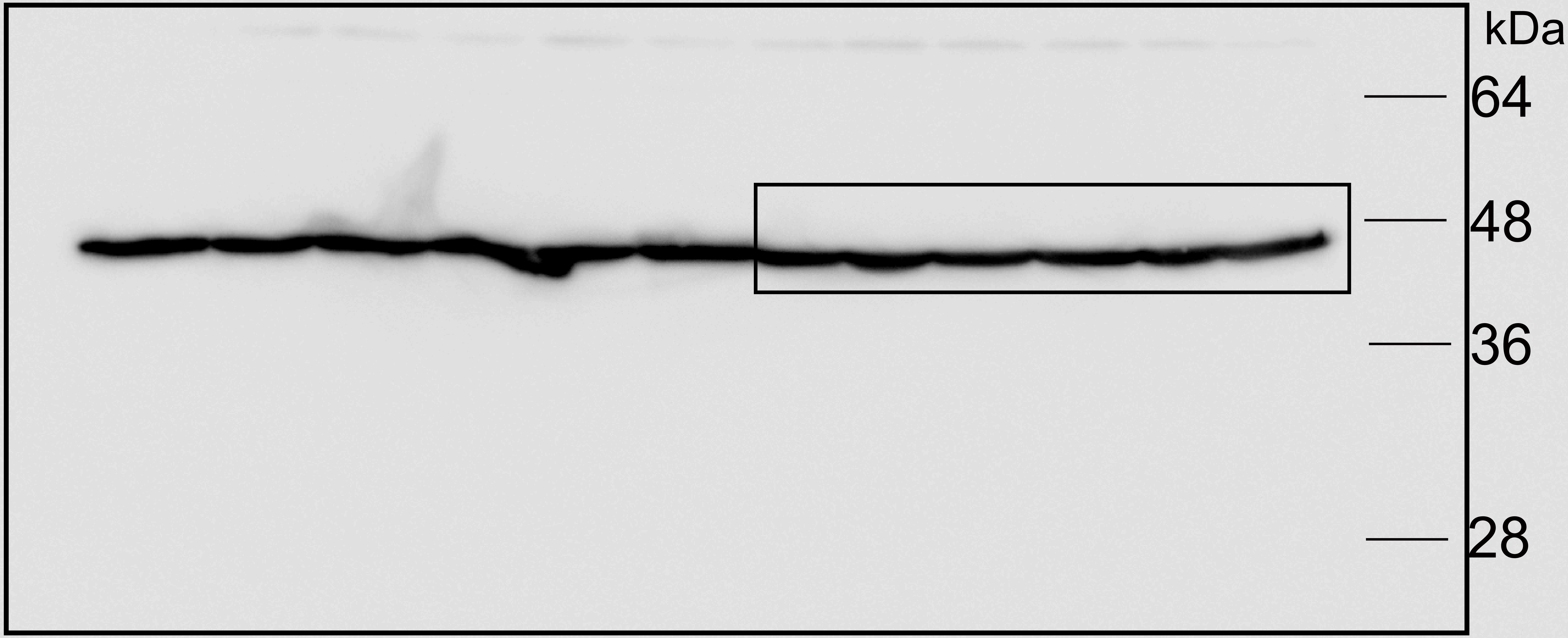

Figure 4E (CTH)
MDA-MB-231

short exposure

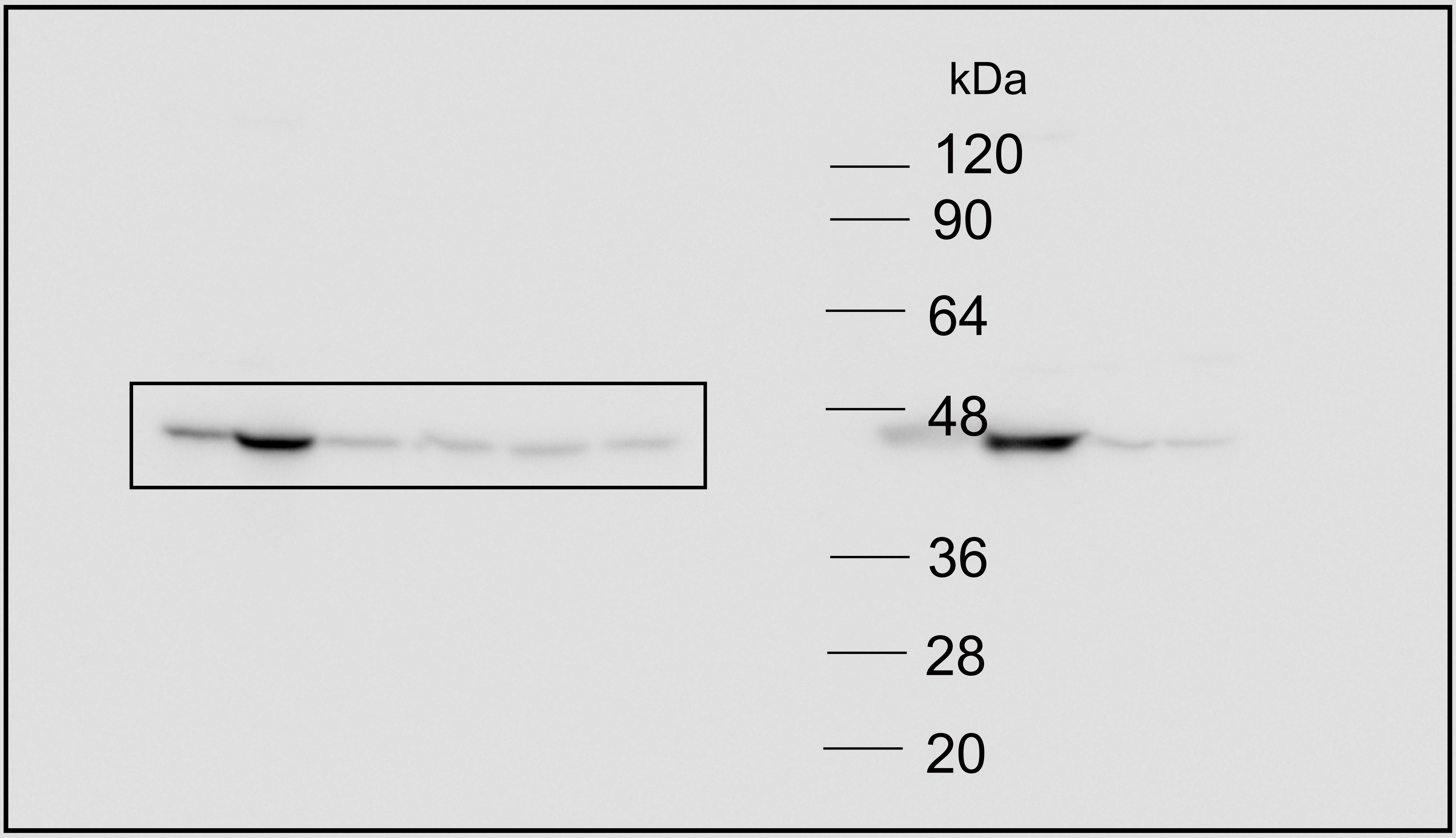

Figure 4E (CTH)
MDA-MB-231

long exposure

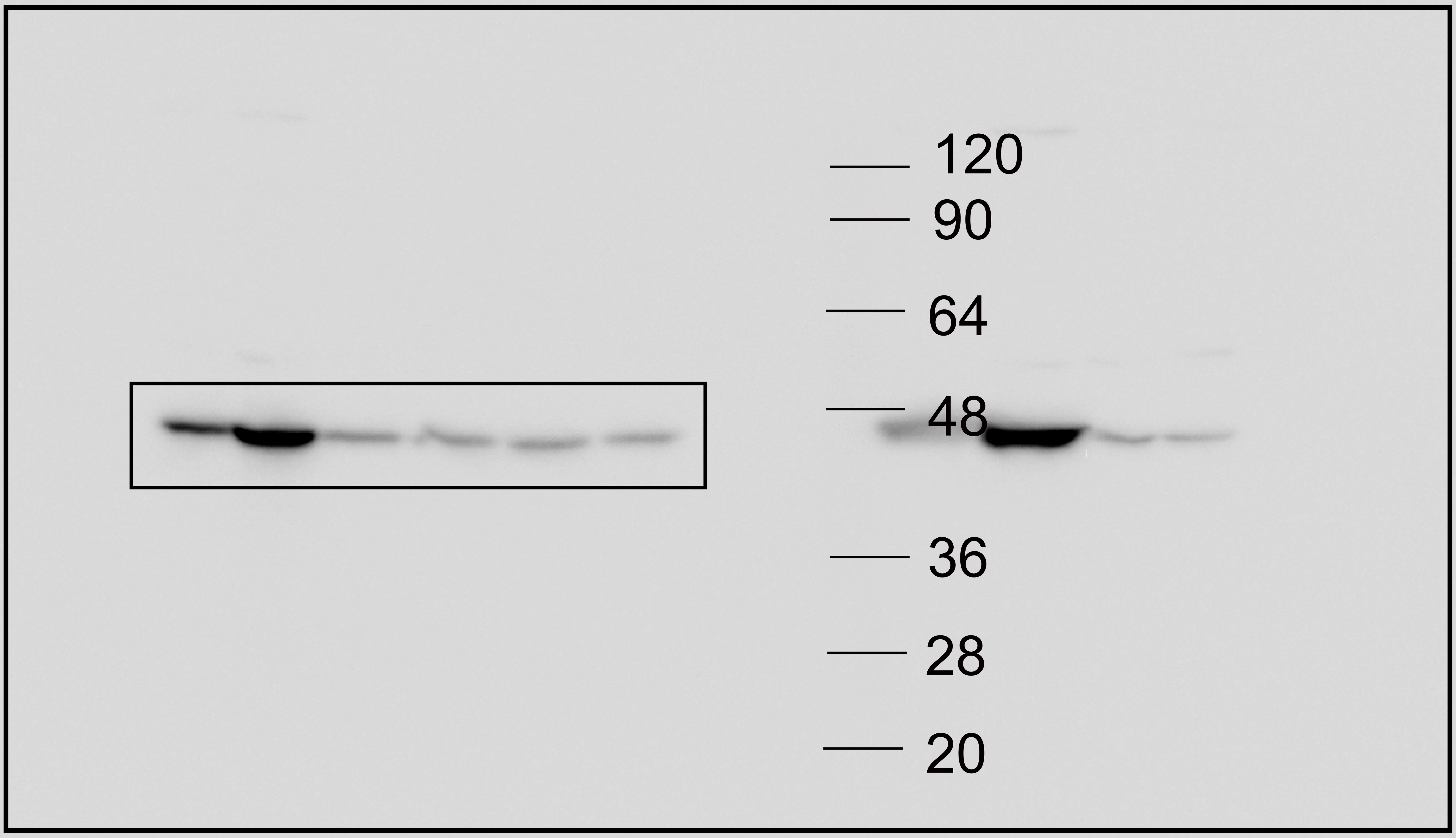

Figure 4E (CTH)
T-47D

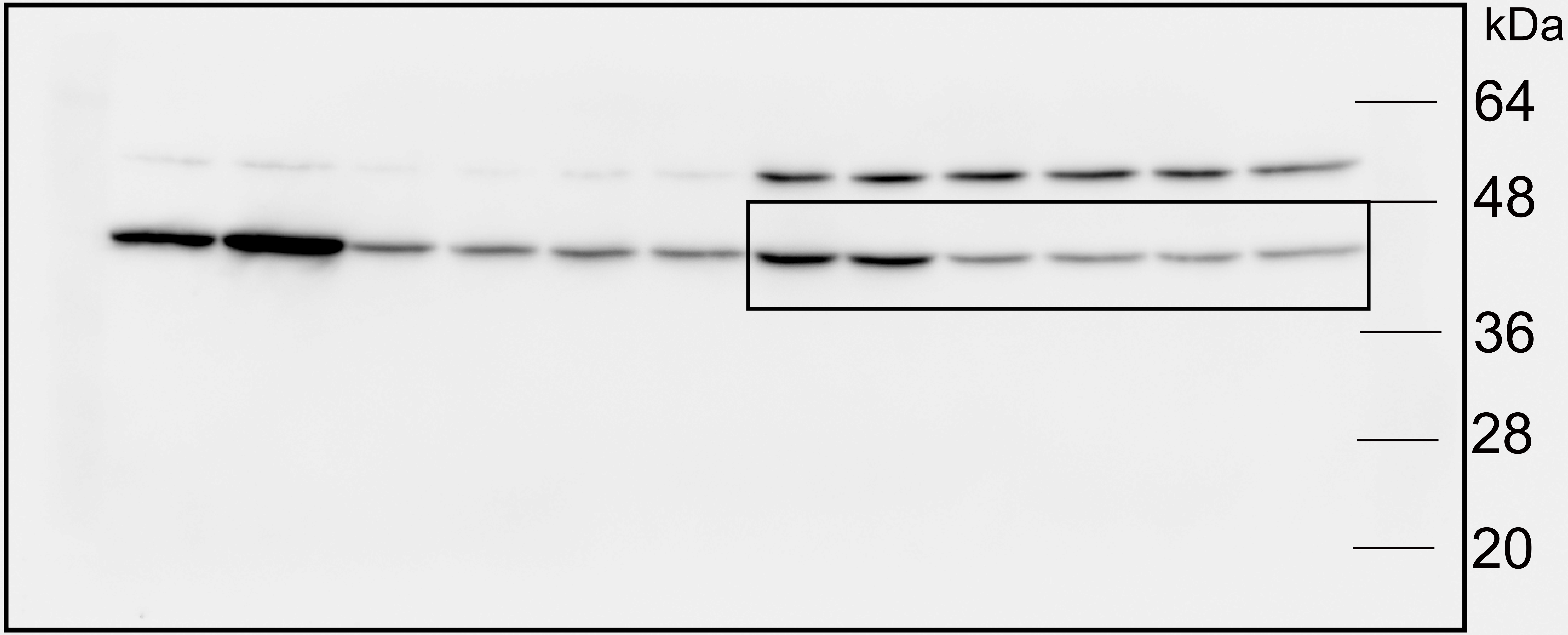

Figure 6A (β-actin)

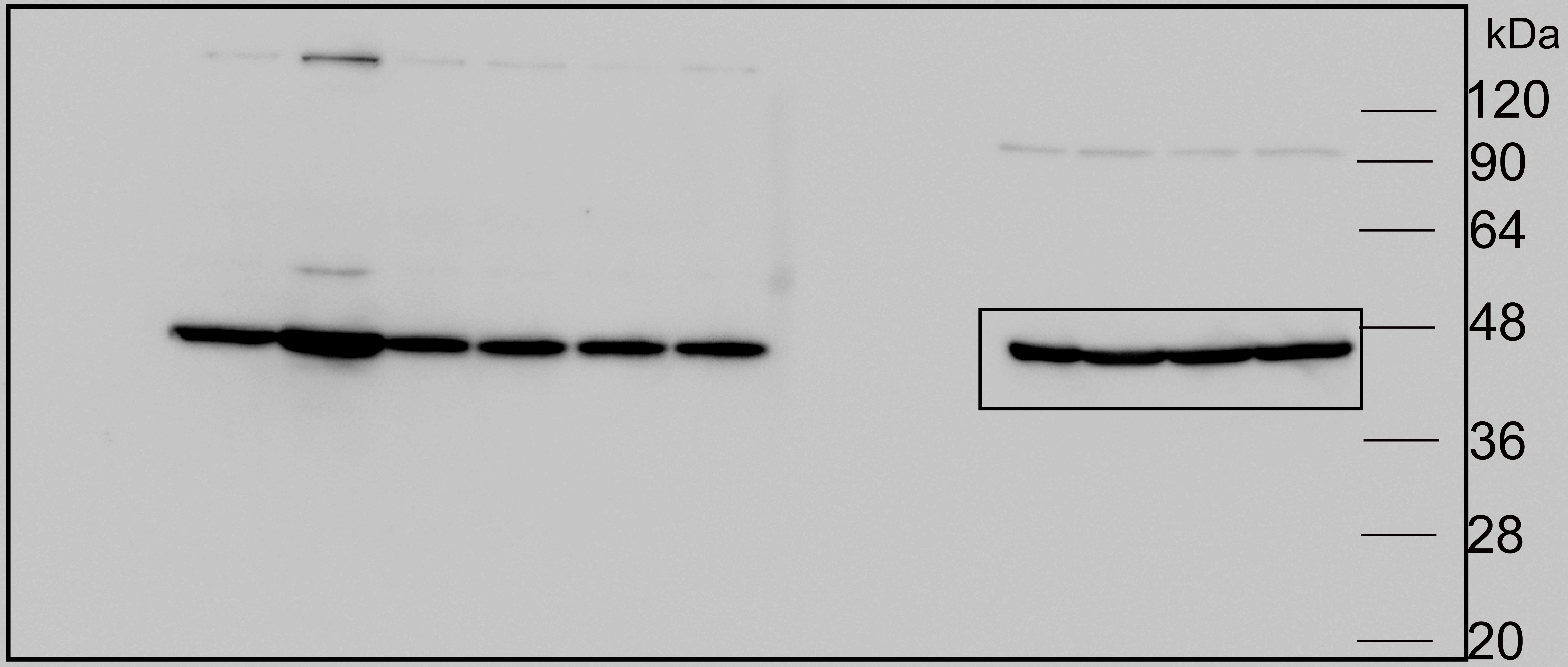

Figure 6A (FLAG)

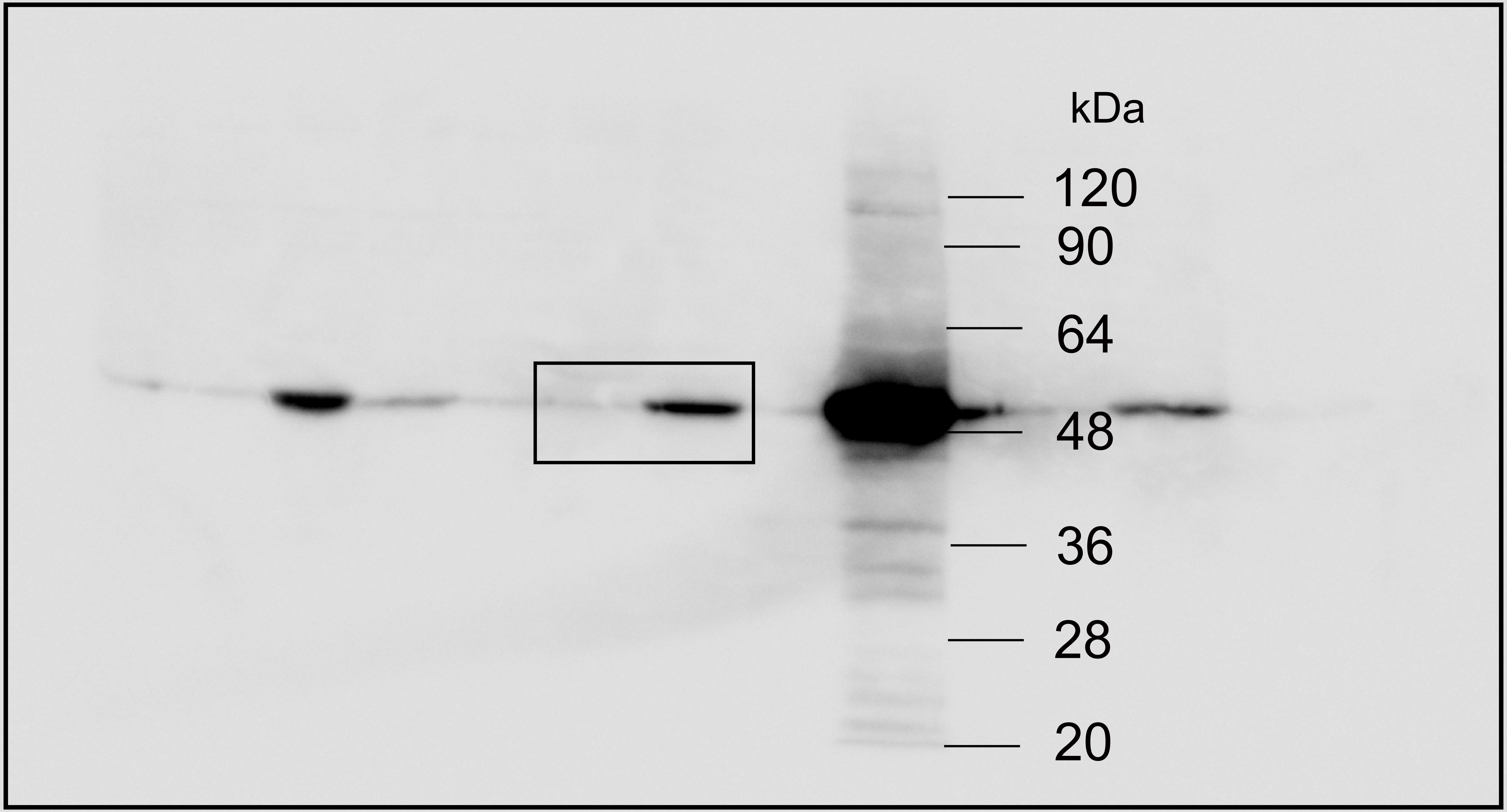

Figure 6A (CTH)

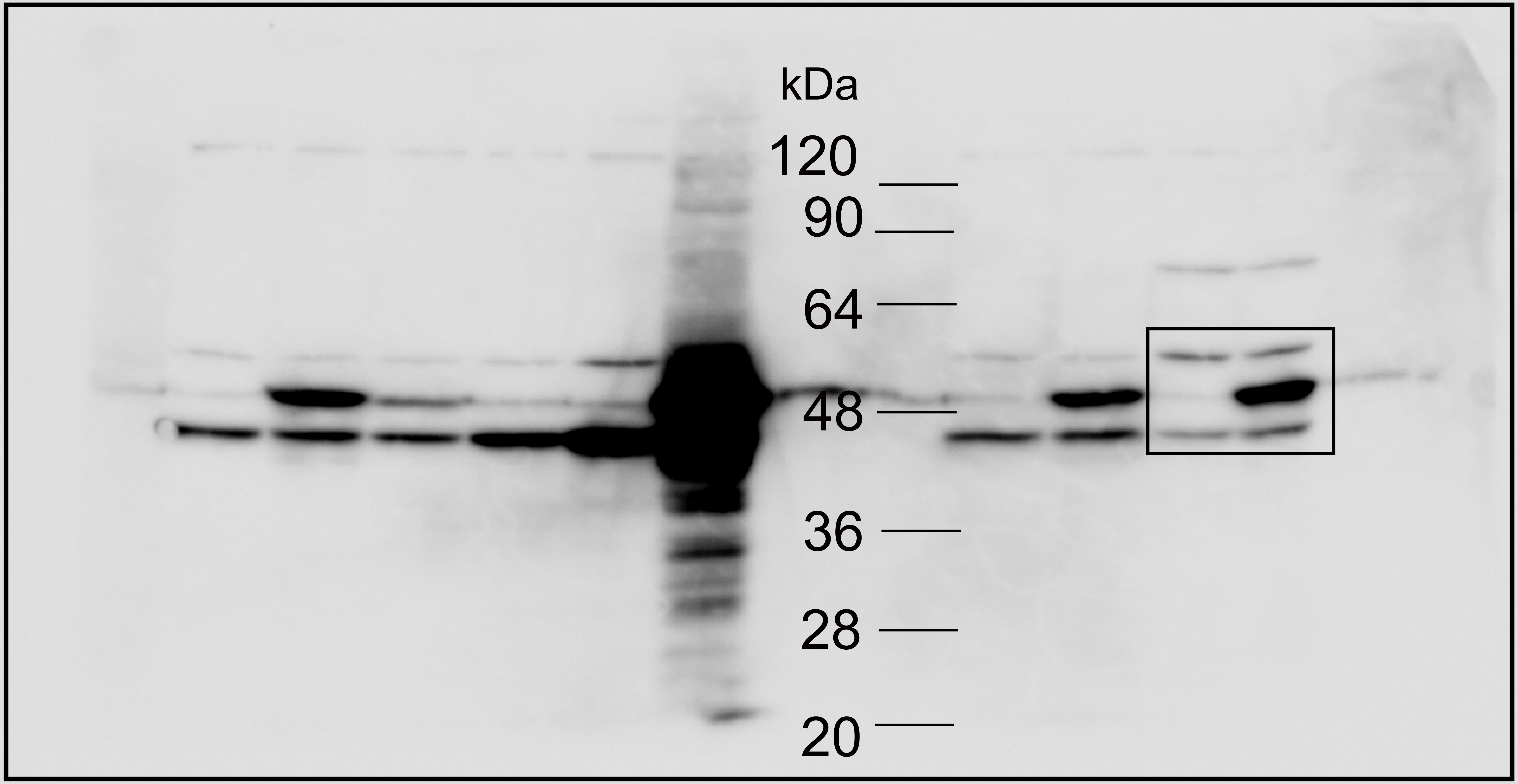

Figure 7B (xCT)

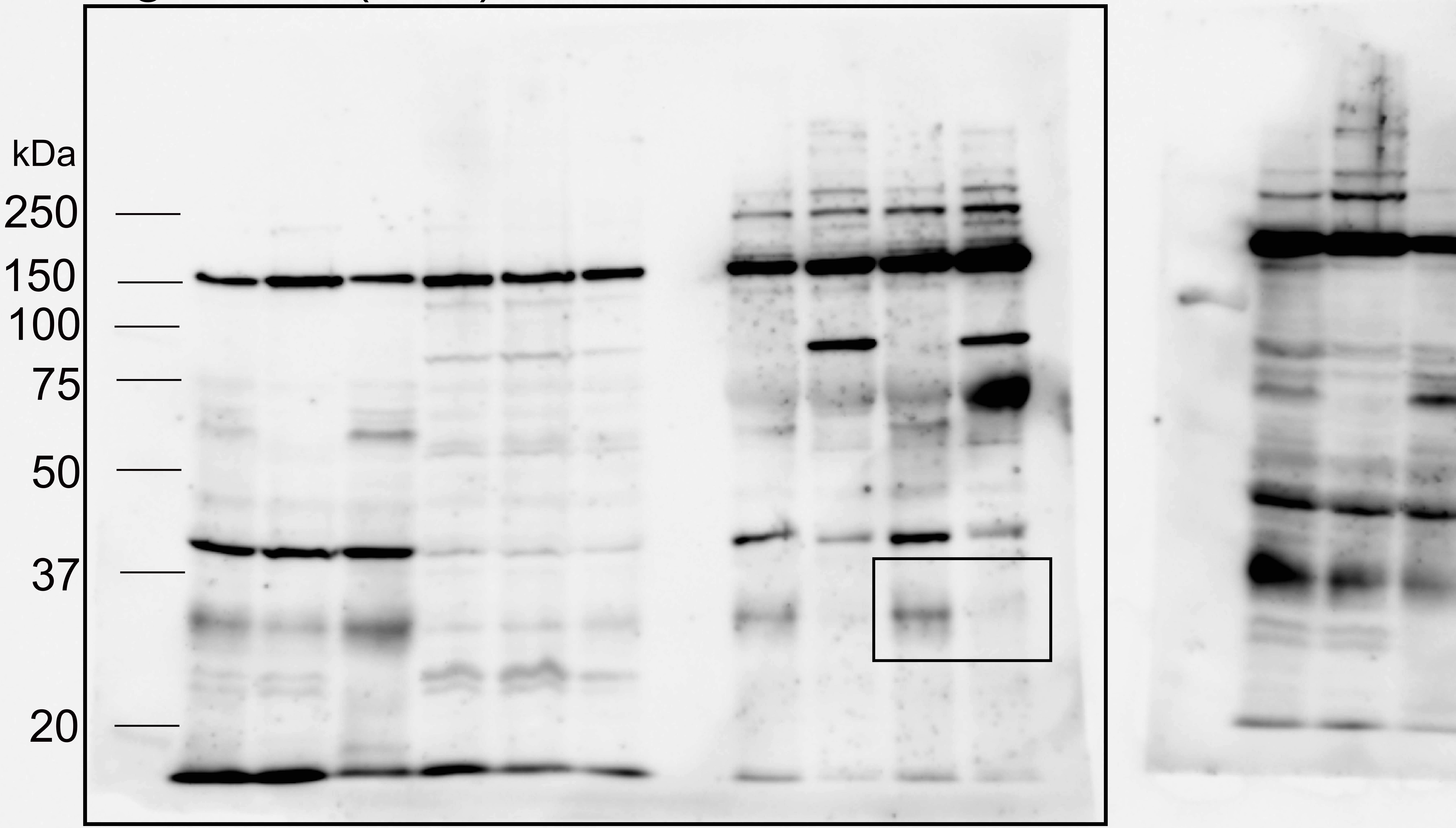

Figure 7B (β-actin)

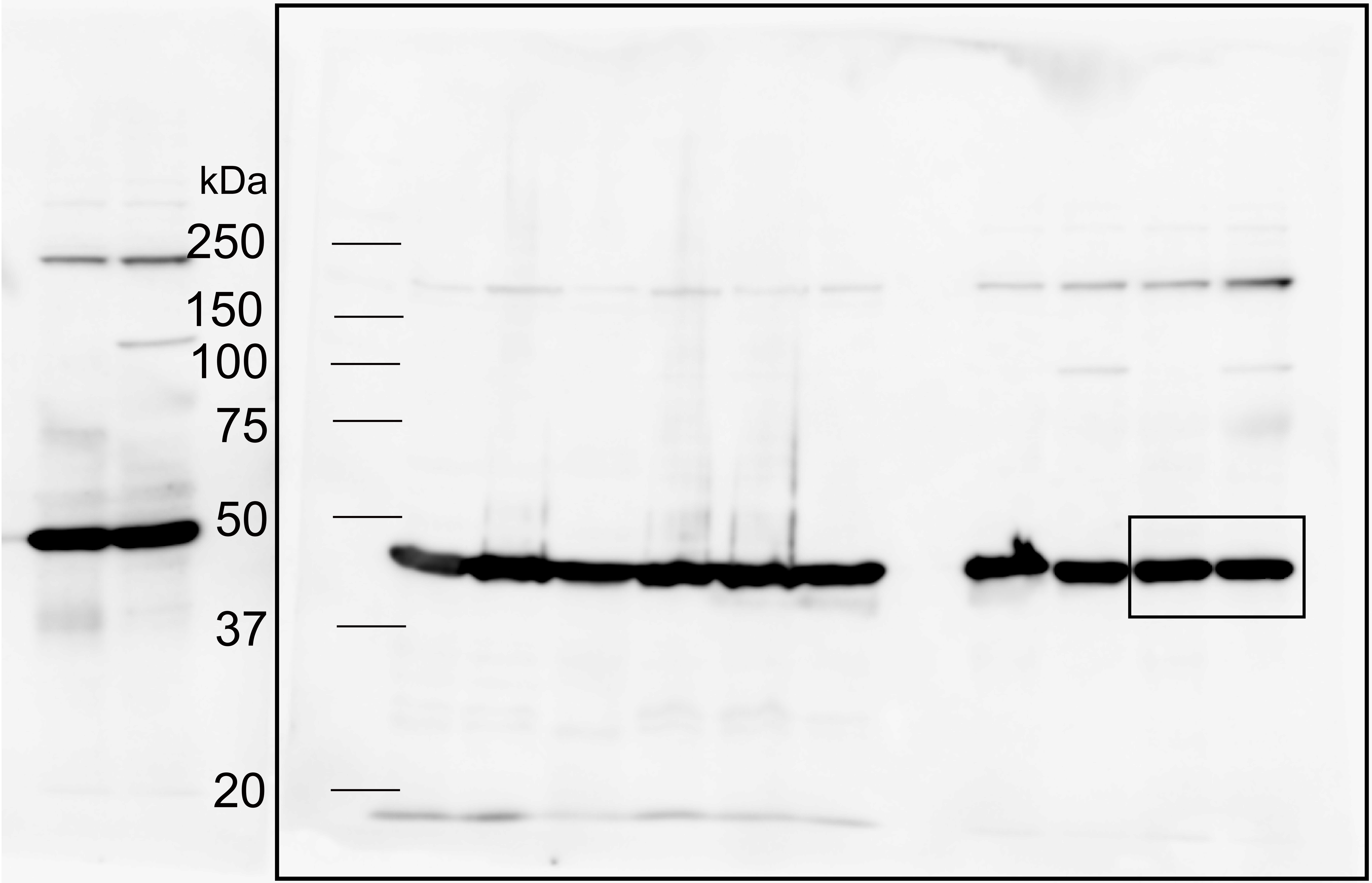

Supplement: Supplementary file 1 — Supplementary Figures. [file 41598_2022_4987_MOESM1_ESM.pdf]
