# Supplementary material for: Induction of CTH expression in response to amino acid starvation confers resistance to anti-LAT1 therapy in MDA-MB-231 cells
Source: Sci Rep. 2022 Jan 19;12:1021. doi: 10.1038/s41598-022-04987-5 (PMC8770514; doi:10.1038/s41598-022-04987-5)
Supplement: Supplementary file 2 — Supplementary Table S1. [file 41598_2022_4987_MOESM2_ESM.pdf]

Supplementary Table S1

RPMI1640 based amino acid restriction medium

		1X	0.5X	0X
**Inorganic Salts:**		mg/L	mg/L	mg/L
Calcium Nitrate・4H2O	$Ca(NO_3)_2 \cdot 4H_2O$	100	100	100
Potassium Chloride	KCl	400	400	400
Magnesium Sulfate	$MgSO_4$	48.84	48.84	48.84
Sodium Chloride	NaCl	6000	6000	6000
Sodium Phosphate,Dibasic	Na_2HPO_4	800	800	800
Sodium bicarbonate	$NaHCO_3$	2000	2000	2000
**Amino Acids:**				
L-Arginine		200	200	200
L-Asparagine		50	50	50
L-Aspartic Acid		20	20	20
L-Cystine・2HCl		65.2	65.2	65.2
L-Glutamic Acid		20	20	20
L-Glutamine		300	300	300
Glycine		10	10	10
L-Histidine		15	7.5	0
L-Isoleucine		50	25	0
L-Leucine		50	25	0
L-Lysine・HCl		40	40	40
L-Methionine		15	7.5	0
L-Phenylalanine		15	7.5	0
L-Proline		20	20	20
Hydroxy-L-Proline		20	20	20
L-Serine		30	30	30
L-Threonine		20	20	20
L-Tryptophan		5	2.5	0
L-Tyrosine・2Na・2H2O		28.83	14.415	0
L-Valine		20	10	0
